# Structure and spectroscopy of propane and propene in the solid state from neutron powder diffraction and inelastic neutron scattering

**DOI:** 10.1107/S2052252526007797

**Published:** 2026-09-11

**Authors:** A. Dominic Fortes, Christopher M. Howard, Stewart F. Parker

**Affiliations:** a ISIS Neutron and Muon Facility STFC Rutherford Appleton Laboratory Harwell Science and Innovation Campus Chilton OxfordshireOX11 0QX United Kingdom; b Forschungs-Neutronenquelle Heinz Maier-Leibnitz (FRM II), Lichtenbergstrasse 1, Garching85748, Germany; ESRF, France

**Keywords:** propane, propene, propyl­ene, neutron diffraction, inelastic neutron spectroscopy, thermal expansion, polymorphism, extraterrestrial compounds

## Abstract

Using high-resolution neutron powder diffraction data, we have refined the previously determined monoclinic structure of the stable β-phase of perdeuterated propane at 10 K and characterized the anisotropic thermal expansion from 10 to 80 K. Using the same methods, we have determined the structure of the stable orthorhombic β-phase of perdeuterated propene for the first time and similarly characterized its thermal expansion between 10 and 85 K. Combining these results with published thermodynamic data, we estimate the magnitude of the volume isotope effect (VIE) in propane and propene to provide parameterizations of the density of each substance in the natural protiated form from limiting low temperature up to their respective melting points. We have been able to establish, using force-field-based structure prediction methods, that the metastable α-phase of propene forms a body-centred tetragonal crystal with a nearly identical packing arrangement to the stable β-phase. This represents an original use of structure prediction methods to obtain results retrospectively from a 28-year-old powder diffraction study with data of low quality, rather than the more usual application of pro-active materials discovery, suggesting promising computational – and perhaps AI or machine learning driven – routes to extracting value from archived historical data. Inelastic neutron scattering spectroscopy, combined with density functional theory calculations, have yielded vibrational mode assignments for β-propane, and for both α- and β-propene, in protiated and deuterated isotopologues. This work provides astronomers with the data required to interpret accurately the infrared spectra of interstellar ice grains and cloud condensates in gas giant planet atmospheres, and provides planetary scientists with information on the temperature-dependent densities necessary to model accurately the behaviour of hydro­carbon sediment grains in various extraterrestrial fluid environments, such as Titan’s ethane rivers and seas.

## Abstract 2

We have carried out a variable-temperature high-resolution neutron powder diffraction study of perdeuterated propane and propene, as well as an inelastic neutron spectroscopy study of both the protiated and deuterated isotopologues of each substance. Coupled with first-principles computer simulations, these provide new insights into the structure and properties of these cosmically abundant molecular solids.

## 1. Introduction

Surprisingly little work has been done on the structures and properties of low molecular weight hydro­carbons, and the literature that exists contains numerous contradictions and outstanding questions. These materials are produced in abundance in interstellar and cold planetary environments, and occur as condensed crystalline and amorphous ‘ices’ in stellar nurseries, planetary nebulae, gas giant planet cloud layers, and the atmospheres of smaller solid icy bodies like Pluto and Saturn’s moon Titan, by the action of high-energy photons and subatomic particles on methane (Roe *et al.*, 2003 ▸; Marcelino *et al.*, 2007 ▸; Nixon *et al.*, 2013 ▸; Andron *et al.*, 2018 ▸). Along with other simple hydro­carbons and nitro­gen-bearing species they form the building blocks of more complex prebiotic molecules such as are observed in the interstellar medium and cometary ices (Öberg, 2016 ▸; Oba *et al.*, 2019 ▸). Accurate detection by remote sensing methods (typically vibrational spectroscopy) and modelling of the behaviour of these ‘ices’ in their astronomical or geological context requires a good foundational knowledge of their structure and properties.

The focus of this work is the C_3_H*_x_* hydro­carbons, which consist of propane (C_3_H_8_), propene (C_3_H_6_) and propyne (C_3_H_4_), which melt at 85.5 K, 88.0 K and 170.5 K, respectively. Propyne also has one isomer, propadiene, that melts at 137.0 K.

The crystal structure of propane was first determined by Boese *et al.* (1999 ▸) from single-crystal X-ray diffraction carried out at 30 K. Those authors reported a monoclinic unit cell (space group *P*2_1_/*n*, *Z* = 4, ρ = 802.7 kg m^−3^).^1^ This structure most likely corresponds with the stable β-phase of propane identified by Pavese & Besley (1981 ▸); the metastable form, α-propane, transforms to β-propane between 79.6 and 81.2 K. Ghosh *et al.* (2018 ▸), using variable-temperature infrared (IR) spectroscopy, similarly observed two phases of propane following vapour deposition at 10 K and subsequent warming, with the first appearing at 50 K and the second at 60 K. More recently, Hudson *et al.* (2021 ▸) confirmed that propane exists in two modifications. ‘Phase I’ crystallized from an amorphous solid between 40 and 50 K, with an irreversible transition to ‘phase II’ occurring on warming of phase I above 60 K. The structure of β-propane was characterized up to 6 GPa at room temperature using laboratory X-ray diffraction methods with a sample contained in a diamond anvil cell (Podsiadło *et al.*, 2013 ▸).

The only effort to determine the structure of propene that we are aware of was made by Lennon *et al.* (2000 ▸). In that work, neutron powder diffraction data were collected from vapour-deposited material at 10 K and 65 K using the same instrument as employed in our study, the High-Resolution Powder Diffractometer (HRPD) at the ISIS Neutron and Muon Facility. Their data, measured at high resolution only in the *d*-spacing range between 2.2 and 4.0 Å, revealed comparatively few Bragg peaks (Fig. S1 in the supporting information), which were substantially broader than the instrumental resolution and which were noted to be superimposed on a significant diffuse background, attributed by the authors to incoherent scattering as a result of inadequate deuteration of their sample. Lennon *et al.* obtained a monoclinic indexing of the powder data but were unable to proceed with structure solution. Inelastic neutron spectroscopy data collected on the ISIS instrument TXFA (now TOSCA, also used in our work) by Lennon *et al.* were used to conclude that propene crystallized in a non-centrosymmetric space group. Of the available primitive non-centrosymmetric monoclinic space groups, *Pc* is ruled out by the systematic absence of the 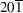 Bragg peak, which is quite clearly observed in the measured diffraction pattern. Thus, on the basis of the measurements by Lennon *et al.*, propene must crystallize in either *P*2, *P*2_1_ or *Pm* (which are indistinguishable given the available data).

Prior to carrying out our own experimental study, we re-examined their data and obtained an alternative body-centred tetragonal (b.c.t.) indexing that superficially appears superior; the figure of merit is higher, there are fewer unobserved peaks, and some of the weak features are matched by the new unit cell that were missed by the originally published indexing. Furthermore, the molar volume obtained from the b.c.t. indexing is in much better agreement with the density reported by Hudson *et al.* (2021 ▸) than is the molar volume resulting from the monoclinic indexing by Lennon *et al.* (2000 ▸) (Table S1 in the supporting information); indeed, the inferred densities their result yields are lower than the density of *liquid* propene (Glos *et al.*, 2004 ▸). As with propane, Hudson *et al.* (2021 ▸) carried out a variable-*T* IR spectroscopy study of propene and observed two phases, the first of which crystallized from the amorphous solid at 70 K and the second at 80 K.

The structure of the perfluorinated analogue of propene, C_3_F_6_, was determined by single-crystal X-ray diffraction at 95 K (Bach *et al.*, 2000 ▸). Hexa­fluoro­propene crystallizes in the triclinic space group 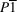 with a layered arrangement of mol­ecules, similar to the layered structure of β-propane.

The lattice parameters between 85 and 160 K, and probable space group, of crystalline propyne were reported recently by Marlin *et al.* (2024 ▸) based on synchrotron X-ray powder diffraction. The authors of that work stated that their data were of too poor quality to carry out a structure solution, although the dataset included in their work appears to us to be of excellent quality. Hudson *et al.* (2021 ▸) reported the density of propyne at 80 K and observed only a single-crystalline phase in their variable-*T* IR measurements, appearing between 70 and 80 K on warming of the amorphous precursor.

No crystallographic work appears to have been done on propadiene thus far.

The vibrational spectroscopy of various C_3_H*_x_* isotopo­logues, namely propane-H8, propane-D8, propene-H6 and propene-D6, has been comprehensively investigated in the gas phase and complete mode assignments are available (Herzberg, 1945 ▸; Shimanouchi, 1972 ▸). In contrast, the solid state has been largely neglected. As noted earlier, solid propane-H8 has been studied by IR spectroscopy over the temperature range 8–80 K under ultra-high vacuum conditions (Ghosh *et al.*, 2018 ▸; Hudson *et al.*, 2021 ▸). Additionally, the pressure dependence of the vibrational spectrum has been studied at ambient temperature up to 40 GPa (Kudryavtsev *et al.*, 2017 ▸) and from 3 to 22 GPa at temperatures from 900 to 3000 K (Kudryavtsev *et al.*, 2020 ▸). The only study of solid propene-H6 we are aware of is the work by Hudson *et al.* (2021 ▸) and there are no reports on the deuterated solid forms of either material.

To date, the published studies have examined the range from 400 to 4000 cm^−1^, covering only a portion of the intramolecular modes. However, the low-energy region (0–400 cm^−1^) includes the lattice modes, the methyl torsion(s) and the C—C—C bending mode. This region is experimentally difficult to study by IR and Raman spectroscopy, and the modes are typically weak, compounding the problem. Inelastic neutron scattering (INS, neutron vibrational spectroscopy) is readily able to access this lower-energy region and because the intensity (in part) depends on the vibrational amplitude of the atoms in the mode, these modes are often the most intense features in the INS spectrum. Since there are also no selection rules in INS spectroscopy, all the modes are allowed (Mitchell *et al.*, 2005 ▸). A low-resolution INS measurement (Grant *et al.*, 1970 ▸) was able to observe the torsional modes in propane and a later measurement provided a spectrum with moderate resolution up to 1000 cm^−1^ (Nelligan *et al.*, 1987 ▸). The INS spectra of propane and propene have been published several times as part of studies of mixed/composite systems: the interaction of propene with carbon catalysts (Lennon *et al.*, 2000 ▸), the oligomerization of propene in ZSM-5 (Hawkins *et al.*, 2019 ▸; Hawkins *et al.*, 2020 ▸), the synthesis of propene from methanol (Lin *et al.*, 2021 ▸), and the separation of propane and propene in metal–organic framework materials (Li *et al.*, 2021 ▸). However, the assignments in these studies have focused on the internal modes of propane and propene; the external modes (translations and librations) have not been characterized. To date, only the fully protiated molecules have been described and there are no INS spectra of the deuterated materials.

## 2. Experimental method

### 2.1. Sample preparation

Deuterated propane (C_3_D_8_, Cambridge Isotope Laboratories, 98 atom % D) and deuterated propene (C_3_D_6_, Cambridge Isotope Laboratories, 96 atom % D) were each condensed as liquids into evacuated glass bulbs using a bath of dry ice. The condensed liquids were decanted directly into an aluminium foil dish of liquid nitro­gen to form solid spherules. The solid spherules were transferred to a nitro­gen-cooled steel cryomortar and ground to a coarse powder with a nitro­gen-chilled steel pestle. For both specimens, the sample holder consisted of an aluminium alloy frame with a central cuboid cavity of dimensions 18 × 23 mm perpendicular to the incident neutron beam and either 15 mm depth parallel to the beam (for the propane sample) or 5 mm depth (propene sample), closed on the beam-in and beam-out sides by 125 µm thick vanadium foil windows held in place with steel frames and sealed to the aluminium body by indium wire. The beam-in sides of the sample holders were masked with a sandwich of Gd and Cd foil to ensure that no parasitic Bragg scattering from steel or Al appeared in the diffraction pattern of the sample. In operation, temperatures are controlled by heating the aluminium frame of the sample holder using a Watlow Firerod cartridge heater, balanced against the cooling power of cold He exchange gas in the sample environment; temperature measurement is achieved with an RhFe sensor inserted on the opposite side of the sample holder to the cartridge heater.

The fully assembled sample holders (front window, masks, sensor, heater) were pre-mounted on sample environment centre sticks ready for immediate insertion into a cold environment. For loading purposes, the sample holders were immersed in liquid nitro­gen with the rear vanadium window removed and the powder samples were rapidly transferred with a nitro­gen-chilled spoon. After filling the sample cavity on each sample holder, the rear window was screwed into place, and the sealed sample holder was carried in a dewar of liquid nitro­gen to the HRPD beamline.

For the INS experiments, each gas was condensed from a known volume attached to a gas manifold into an indium-wire sealed aluminium cell, immersed in liquid nitro­gen. The sample holder’s internal dimensions were 48 × 60 mm (width × height) perpendicular to the incident neutron beam and 1.5 mm depth parallel to the beam, with 3.85 mm thick aluminium alloy on the beam entry and exit windows. When condensation ceased, the cell was immediately transferred into the closed-cycle refrigerator (CCR) on the TOSCA beamline (Parker *et al.*, 2014 ▸; Pinna *et al.*, 2018 ▸) and cooled to ∼10 K. The CCR was pre-cooled to ∼20 K, so the sample temperature was always at, or below, 77 K between loading and measurement. The same procedure was used for all four compounds: propane-H8 (CK Gas 99.95%), propane-D8 (Cambridge Isotope Laboratories 98 atom % D), propene-H6 (CK Gas 99.5%) and propene-D6 (Cambridge Isotope Laboratories 96 atom % D).

### 2.2. Measurements

A CCR was mounted in the sample vacuum vessel of the High-Resolution Powder Diffractometer (HRPD) at the ISIS Neutron and Muon Facility and the cold-head temperature reduced to 80 K. Once the centre stick with the sample holder was inserted into the CCR, the He exchange gas was rapidly flushed through and neutron powder diffraction data were acquired to evaluate the crystallinity and phase identification of the samples. An initial dataset from propane-D8 was obtained at 70 K and from propene-D6 at 80 K.

HRPD typically measures at 10 Hz with time-of-flight (TOF) frames of 100 ms duration; the chopper openings may be phased arbitrarily, but time frames of 30–130 ms and 100–200 ms are used most commonly. In the instrument’s highest resolution backscattering detectors, these TOF windows provide access to *d* spacings from 0.65–2.60 and 2.20–3.90 Å, respectively. The longer *d*-spacing range is particularly useful for measurement of well dispersed peaks with low Bragg indices for phase identification and unit-cell indexing purposes.

Data from propane-D8 were measured using the 30–130 ms TOF window in 5 K increments from 50 K down to 10 K (in practice the sample temperature bottomed out at 10.25 K), accumulating 20 µA of proton beam current at each datum, equivalent to ∼30 min of real time. Temperature changes were controlled on a ramp of 3 K min^−1^, with a dwell time of 10 min after reaching each set point to allow for adequate thermal equilibration of the sample. At 10.25 K, longer datasets (80 µA, ∼2 h) were obtained in both 30–130 ms and 100–200 ms TOF windows. Thereafter, 20 µA datasets were collected on warming (using the same ramp and equilibration times) in 5 K increments interleaved between the cooling measurements (*i.e.* 12.5 K, 17.5 K *etc.* up to 47.5 K) and then in 5 K increments from 55 to 80 K using only the 30–130 ms TOF window. At 80 K, data were collected for 40 µA (∼1 h).

The measurement strategy for propene-D6 was broadly similar, with the notable difference that the structure was unknown and the specimen was substantially smaller than the propane-D8 sample. Data were first obtained using the 30–130 ms and 100–200 ms TOF windows at 80 K, counting each for 160 µA (∼4.5 h). Thereafter, data were collected in 10 K increments from 70 K down to 10 K, accumulating 40 µA of proton beam current at each datum. As before, temperature changes were controlled on a ramp of 3 K min^−1^ with a dwell time of 10 min for thermal equilibration. At 10 K, high-quality datasets were obtained in both 30–130 ms and 100–200 ms TOF windows for 320 µA (9 h) and 160 µA (4.5 h), respectively. Thereafter, 40 µA datasets were collected on warming in 10 K increments interleaved between the cooling measurements (*i.e.* 15 K, 25 K *etc.* up to 85 K) using only the 30–130 ms TOF window. At 85 K, a sequence of three one-hour measurements were acquired. Despite the proximity of the sample’s melting point, no changes in the diffraction patterns obtained at 85 K were noticed and the three datasets were simply summed together.

A top-loading CCR was similarly mounted in the sample vacuum vessel of the TOSCA spectrometer at the ISIS Neutron and Muon Facility. For the INS measurements, the samples were cooled to base temperature, between 10–20 K, and measured with the instrument’s standard settings (Parker *et al.*, 2014 ▸). Both propane-H8 and -D8 were measured for ∼1100 µA (∼8 h), propene-H6 was measured for ∼680 µA (∼5 h) and propene-D8 was measured for ∼1600 µA (∼12 h).

### 2.3. Data processing

All HRPD data were processed using the *Mantid* suite of powder diffraction algorithms (Arnold *et al.*, 2014 ▸), subtracting empty instrument backgrounds, normalizing to the incident beam’s spectrum and correcting for instrument efficiency by reference to a V:Nb null-scattering sample, correcting for calibrated detector offsets, and finally time-focusing the spectra from each detector bank. The resulting 1D histograms were exported as multibank files for analysis with *GSAS-I* and *Expgui* (Larson & Von Dreele, 2004 ▸; Toby, 2001 ▸).

The INS data were converted from time of flight to energy transfer using the routines built into *Mantid*. The spectrum of the empty Al can was subtracted from all of the hydro­carbon INS spectra.

### 2.4. Diffraction analysis (propane-D8)

The diffraction patterns of propane-D8 were found to match the known monoclinic structure (the stable phase β-propane, or propane-II) and no phase transitions were observed on cooling or warming. Consequently, the analysis was straightforward. All data were subjected to profile refinement using the ‘F(Calc) Weighted’ method of intensity extraction in the *GSAS-I* analysis software, varying only the lattice parameters, peak-profile coefficients and background (modelled with a 12-term Shifted Chebyschev polynomial), to produce high-precision lattice parameters as a function of temperature (Table S2 and Fig. S2). The crystal structure of propane-D8 was refined using the Rietveld method at 10 K starting from the structural model of Boese *et al.* (1999 ▸), using a fully anisotropic description of the atomic displacement parameters (ADPs), and refining a wavelength-dependent absorption correction and overall scale parameter in addition to the previously mentioned terms. The final fit to the 10 K powder diffraction data is shown in Fig. S3 and the structural model has been deposited in the Cambridge Structural Database (CSD) (No. 2584087).

### 2.5. Diffraction analysis (propene-D6)

**Figure 1:**
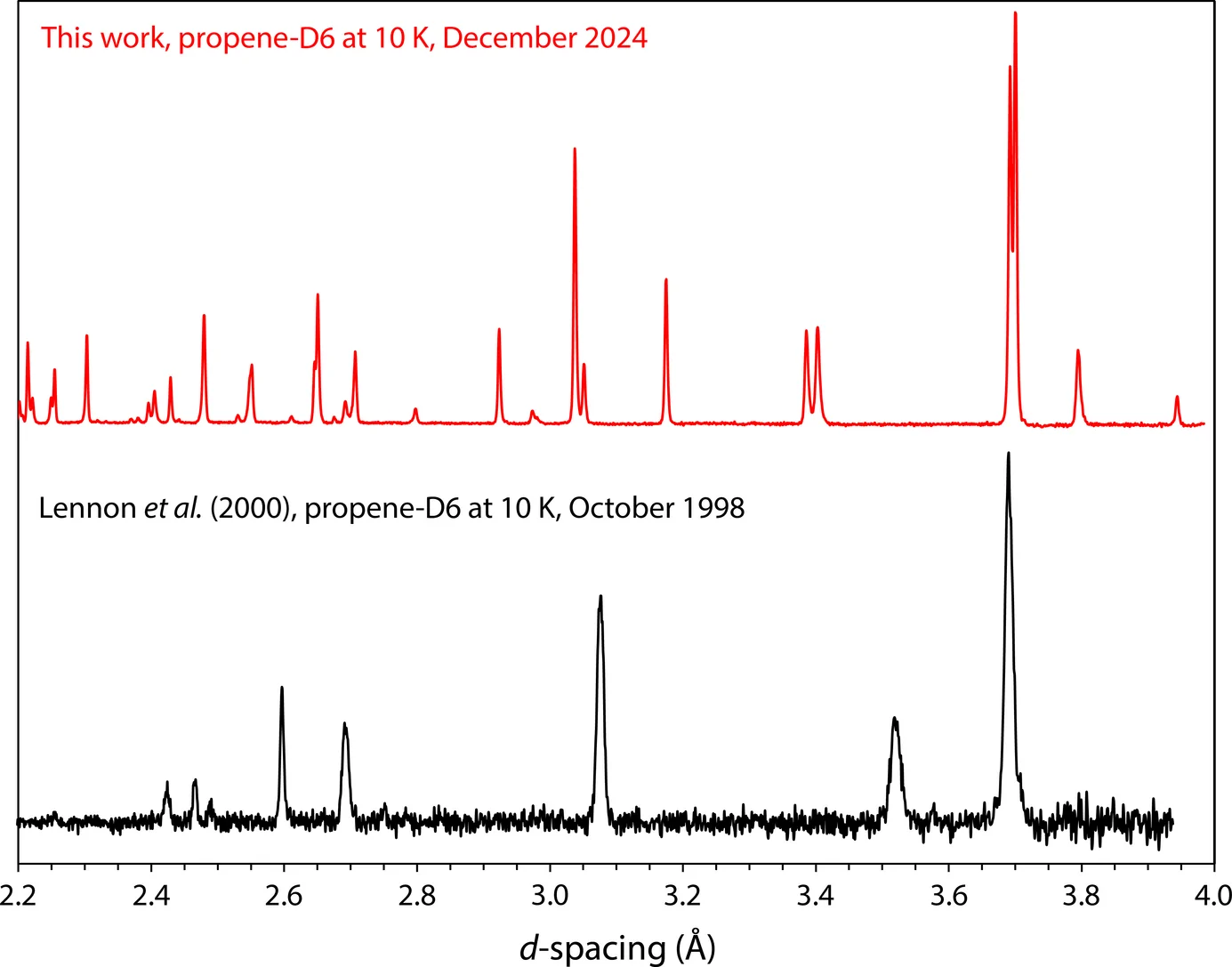
Comparison of neutron powder diffraction patterns measured from ostensibly perdeuterated propane in October 1998 (bottom, in black) and December 2024 (top, in red) on HRPD.

The diffraction patterns of propene-D6 did not match those observed previously on HRPD (Fig. 1 ▸), although there are some similarities. We therefore used the initial 80 K dataset measured in the 100–200 ms TOF window to extract 15 Bragg peak positions and index them using *DICVOL06* (Louër & Boultif, 2007 ▸). An orthorhombic solution with very high figures of merit was obtained: *a* = 10.8834 (5) Å, *b* = 10.8148 (6) Å, *c* = 5.8029 (4) Å, *V* = 683.01 Å^3^, *M*(15) = 158.6 and *F*(15) = 239.0 (0.0018, 35) (de Wolff, 1968 ▸; Smith & Snyder, 1979 ▸). The obtained unit-cell volume is consistent with *Z* = 8. Analysis of the systematic absences revealed the space group to be *P*2_1_2_1_2_1_. No further changes in the diffraction pattern, other than peak shifts attributable to anisotropic thermal expansion, occurred on cooling to 10 K. Indexing of the well counted 100–200 ms data at 10 K yielded essentially the same orthorhombic solution with similarly high figures of merit. The obtained unit cell is strikingly similar to our tetragonal indexing of the 1998 HRPD data cited in the *Introduction*[Sec sec1], both the orthorhombic and tetragonal unit cells being approximately 11 × 11 × 5.5 Å. It is also worth observing that the b.c.t. unit-cell dimensions we indexed at 10 K and 65 K are both 2.3% larger than the primitive orthorhombic unit cell at the same temperatures, and so the thermal expansion of the sample studied on HRPD in 1998 was the same as for our 2024 sample. The tetragonal crystal expanded by 1.485% on heating from 10 to 65 K and our orthorhombic crystal expanded by 1.533% over the same range. Contrast this with the expansion obtained using the primitive monoclinic unit cell from Lennon *et al.* (2000 ▸), 2.194%. The similarity in overall dimensions, axial ratios and thermal expansion supports our view that the tetragonal indexing is correct, and we further infer that the previous study on HRPD observed a distinct metastable phase of propene that probably exhibits some underlying structural relationship with the stable phase obtained in our study. Both the high symmetry and the differences in molar volume, and perhaps also the diffuse background attributed by Lennon *et al.* to partial protiation of their sample, might result instead from orientational disorder of the tetragonal phase.

Following indexing of the 80 K dataset, it was possible to carry out structure-less ‘F(Calc) Weighted’ profile refinements on all the available datasets, varying lattice parameters, peak-profile coefficients and the background (again, using a 12-term shifted Chebyschev polynomial), to obtain high-precision unit-cell parameters as a function of temperature (Table S3 and Fig. S4).

The structure of orthorhombic propene-D6 was then solved using the simulated annealing algorithm implemented in *FOX* (Version 1.8.1.2-R1117; Favre-Nicolin & Černý, 2002 ▸; Černý *et al.*, 2017 ▸). A description of the propene molecule in the form of a *Z*-matrix was used as the basis for the structure solution, using two symmetry-inequivalent copies of the molecule to yield eight molecules per unit cell once the *P*2_1_2_1_2_1_ symmetry of the crystal was accounted for. These molecules were forced to behave as rigid units during the optimization. Twenty runs of two million trials each produced four outcomes where the cost function dropped an order of magnitude lower than the others, in which the calculated diffraction pattern matched the observed diffraction pattern extremely well. The result with the lowest cost function was then used as the basis for Rietveld refinements of the high-quality datasets measured at 10 K and 80 K.

The newly solved crystal structure of propene-D6 was thus refined at 10 K and 80 K using a fully anisotropic description of the ADPs, refining a wavelength-dependent absorption correction and overall scale parameter in addition to the previously mentioned terms. The final fits to the 10 K and 80 K powder diffraction data are shown in Figs. S5 and S6 and the structural models have been deposited in the CSD (Nos. 2584086 and 2584085, respectively.)

## 3. Computational methods

To aid in our analysis, a variety of computer simulations of propane and propene structures were carried out. Density functional theory (DFT)-based geometry optimizations were done with *CASTEP* (Version 23; Payne *et al.*, 1992 ▸; Clark *et al.*, 2005 ▸) in order to obtain the ground-state structure of the propane and propene molecules in the periodic crystal in the athermal zero-pressure limit (in experimental practice, ‘zero’ pressure is zero applied pressure, *i.e.* atmospheric pressure, 100 kPa). Calculations were started from the refined experimental structures using the PBE generalized gradient approximation (Perdew *et al.*, 1996 ▸) and the MBD* many-body dispersion correction to account for van der Waals forces (Tkatchenko *et al.*, 2012 ▸), on-the-fly generated (OTFG) ultrasoft pseudopotentials, and an energy cutoff of 1000 eV combined with moderately fine Monkhorst–Pack grids of *k*-points having reciprocal-space intervals of ∼0.04 Å^−1^. Criteria for convergence were total energies <5 × 10^−6^ eV atom^−1^, forces <0.01 eV Å^−1^, stress tensor components <0.01 GPa and displacements <5 × 10^−4^ Å. The resulting DFT-derived structures for propane and propene are included in the supporting information and various geometric properties are discussed in the following sections.

We also used computational methods to derive candidate structures that could match the experimental data of Lennon *et al.* (2000 ▸) for propene. This was done with the *Polymorph* module in *Materials Studio* (Shankar *et al.*, 2022 ▸), which takes a force-field based approach to exploring the packing parameter space of atoms and molecules, subject to user-imposed symmetry constraints, and computing the energies of each structure for ranking purposes. In the first instance, a propene molecule was created in *Materials Studio* and geometry optimized with DFT methods using *DMol^3^* (Delley, 2000 ▸) working inside the *Materials Studio* environment. The PBE GGA (Perdew *et al.*, 1996 ▸) was employed with the Tkatchenko–Scheffler dispersion correction (Tkatchenko & Scheffler, 2009 ▸), an all-electron core treatment using the double numerical polarized (DNP+) basis set and ‘Medium’ levels of integration accuracy, self-consistent-field tolerance and orbital plane-wave cutoff quality. The *DMol^3^* calculations provided accurate molecular geometries and electrostatic potentials, which were applied to the atoms before proceeding to the structure-prediction stage.

In the *Polymorph* module of *Materials Studio*, the ‘Prediction’ task was customized to provide ‘Ultra-fine’ quality settings for the Packing, Clustering and Geometry Optimization steps. Energies were computed with the consistent valence force field (CVFF) (Maple *et al.*, 1988 ▸), which we know from experience to work well with small organic mol­ecules, and atomic charges derived from the* DMol^3^* electrostatic potentials. We focused on b.c.t. symmetry for the parameter-space searches and carried out tests using only the eight space groups consistent with the systematic absences in the observed data collected in 1998, specifically *I*4, 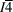, *I*4/*m*, *I*422, *I*4*mm*, 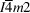, 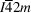 and *I*4/*mmm*. The results of this work pointed to one very clearly preferred candidate structure in space group 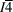, with lattice parameters closely matching those we obtained from indexing the powder data of Lennon *et al.* (2000 ▸), which was then subjected to a cycle of DFT geometry optimization using *CASTEP* and which will be discussed briefly later.

*CASTEP* was similarly employed for calculation of the vibrational spectra with essentially the same computational recipe as was used for the geometry optimizations: PBE GGA functional, Tkatchenko–Scheffler dispersion correction scheme and OTFG norm-conserving pseudopotentials. The plane-wave cut-off was 720 eV in all cases. The Brillouin-zone sampling of electronic states for propane used a 7 × 7 × 11 Monkhorst–Pack grid (156 *k*-points), for the *P*2_1_2_1_2_1_ phase of propene a 7 × 7 × 11 Monkhorst–Pack grid (96 *k*-points) was used, and for the 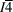 phase an 8 × 8 × 6 Monkhorst–Pack grid (180 *k*-points) was used. Phonon energies were then obtained by diagonalization of dynamic matrices computed using density-functional perturbation theory (Refson *et al.*, 2006 ▸). Phonon dispersion was calculated along various high-symmetry directions throughout the Brillouin zones of each crystal simulated. Dynamic matrices were computed on a regular grid of wavevectors throughout the Brillouin zone and Fourier interpolation was used to extend the computed grid to the desired fine set of points along the high-symmetry paths (Gonze *et al.*, 1994 ▸). The spectra of the isotopically substituted species were calculated within the harmonic approximation using the *CASTEP* utility *Phonons* (Refson, 2013 ▸) and the INS spectra were generated from the *CASTEP* output with the *AbINS* code (Dymkowski *et al.*, 2018 ▸), which is part of the *Mantid* package.

## 4. Results

### 4.1. The structure of propane-D8

The stable β-phase of propane has a layered structure with approximately close-packed rafts of propane molecules lying in the (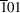) plane, having an undulation wavevector along the [010] direction with an amplitude of ∼1 Å and a wavelength equal to the length of the *b* axis (∼12.5 Å), being stacked with an *A*–*B*–*A*–*B* repeat sequence along the (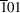) plane normal. Molecular interactions (intermolecular contacts and inter­action energies) were computed using *CrystalExplorer* (Version 21.5; Spackman *et al.*, 2021 ▸). From a determination of the Hirshfeld surface and related fingerprint plots (Fig. S7), we note that all of the interactions are H⋯H contacts, quite narrowly spatially clustered in terms of length, which differs from the structure of propene described below. Nearest-neighbour molecules in the plane of the corrugated sheets vary in distance from one another by 4.76 to 6.34 Å with total interaction energies of −2.6 to −5.9 kJ mol^−1^, these energies being principally a mix of strongly negative dispersive energies and positive repulsive energies. The strongest interactions are with near neighbours in adjacent sheets (at radial distances of 4.12 Å) with total energies of −7.8 kJ mol^−1^. As we will see later, these stronger inter-sheet interactions are what determine the distribution of the thermal expansion anisotropy.

**Figure 2:**
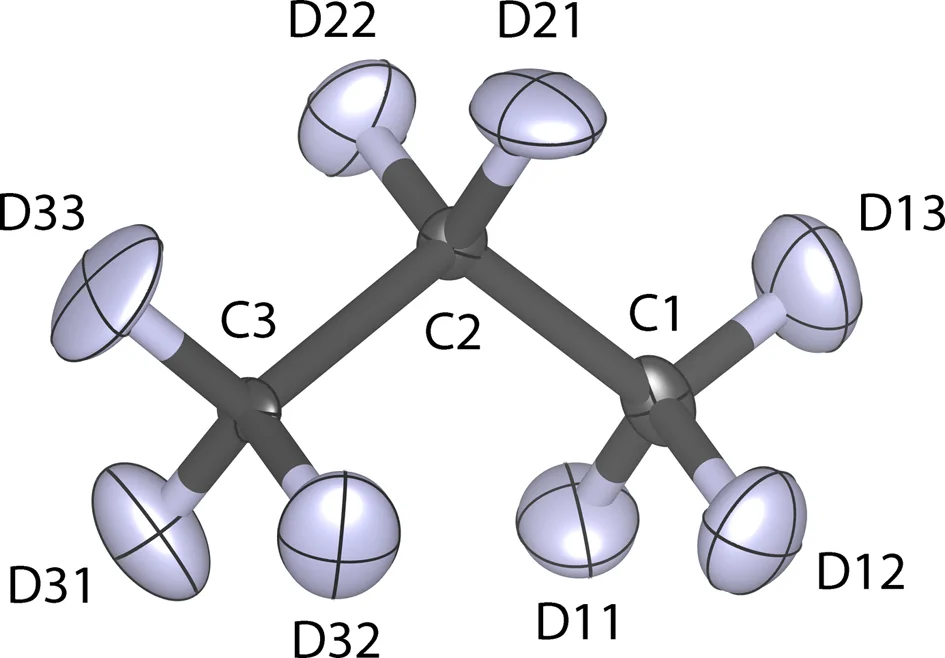
Refined geometry and anisotropic displacement parameter ellipsoids (drawn at the 75% probability level) for propane-D8 in the solid state at 10 K (see Table S4 for bond lengths and angles).

Table S4 compares the geometry of the propane molecule (Fig. 2 ▸) found experimentally [C_3_D_8_ in this work, C_3_H_8_ in Boese *et al.* (1999 ▸)] with that obtained from a DFT geometry optimization of the crystal structure. Although the unit-cell parameters are significantly under-estimated by DFT, the molecular geometry appears to be accurate. Note that our PBE + MBD* result differs from that of Boese & Sauer (2017 ▸), who found good agreement with experimental molar volumes and lattice energies with this combination of GGA and dispersion correction. Whilst our 10.25 K refinement yields two significantly different C—C distances, the average of 1.526 Å closely matches the C—C bond lengths in the DFT calculations (1.5273 Å). It is entirely unsurprising that the C—H bond lengths reported by Boese *et al.* (1999 ▸) are inaccurate, since the weak X-ray scattering density associated with the hydrogen atom corresponds with a peak in electron density displaced along the C—H bond away from the hydrogen nucleus itself. Clearly, their lower precision on the C—H bond lengths and angles is also a feature of the tiny X-ray scattering factor of hydrogen, which does not apply in our neutron scattering study.

### 4.2. The structure of orthorhombic propene-D6

**Figure 3:**
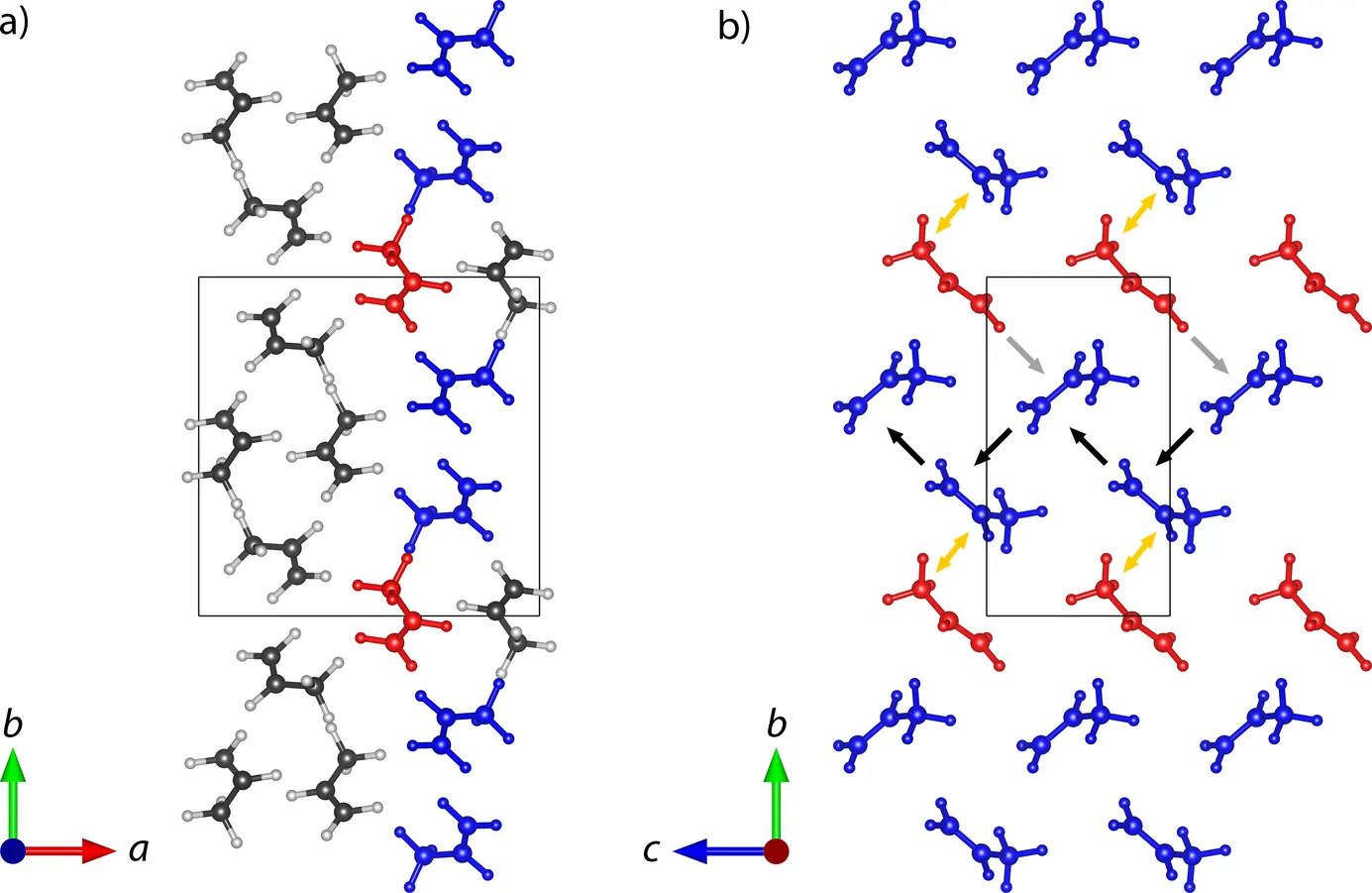
The refined structure of propene at 10 K viewed (*a*) along the *c* axis and (*b*) along the *a* axis. Symmetry-inequivalent molecules are colour coded, those in blue being the P1 molecules with the C1—C2—C3 backbone, and those in red being the P2 molecules with the C4—C5—C6 backbone. The illustration on the right also indicates the more direct T-shaped interactions with black arrows, the less direct T-shaped interactions with grey arrows and the planar interactions with double-ended orange arrows.

Unlike propane, and unlike hexa­fluoro­propene, the structure of propene-D6 consists of a three-dimensional arrangement of interlocking canted molecules. As shown in Fig. 3 ▸, the P1 propene molecules (defined by the C1—C2—C3 backbone) form chains with relatively direct T-shaped interactions where the methyl­ene group of one P1 molecule points towards the carbon backbone of the nearest neighbour along the *c* axis, more specifically towards the π-bond in the C=C double bond between C1 and C2. The P2 molecules (defined by the C4—C5—C6 backbone) form somewhat oblique T-shaped interactions with adjacent P1 molecules, as well as planar interactions, where the carbon backbones of neighbouring P1 and P2 molecules are approximately parallel. These differences are manifested in the fingerprint plots derived from Hirshfeld surfaces computed with *CrystalExplorer* (Fig. S8), where we observe that the interactions are no longer purely H⋯H, as in propane, but now include ∼12% of the Hirshfeld surface area devoted to H⋯C interactions. This is to be expected as the hydro­carbon becomes less saturated, and we anticipate the fraction of H⋯C interactions being even larger in propyne and propadiene. The interaction energies, also computed in *CrystalExplorer*, are broadly similar to those found in propane. The more directional T-shaped C—H⋯π interactions between adjacent P1 molecules have energies of −4.9 kJ mol^−1^, whilst the less direct T-shaped interactions between P1 and P2 molecules have energies of −3.2 kJ mol^−1^. The strongest interactions are between parallel P1–P2 pairs at radial separations of 3.98 Å, with energies of −8.1 kJ mol^−1^. The distinction in packing between propene and hexa­fluoro­propene probably reflects the lower polarizability of C—F versus C—H bonds, the more anisotropic molecular quadrupole moment of C_3_F_6_ and stronger F⋯F repulsion, all of which favour planar interactions over T-shaped interactions.

**Figure 4:**
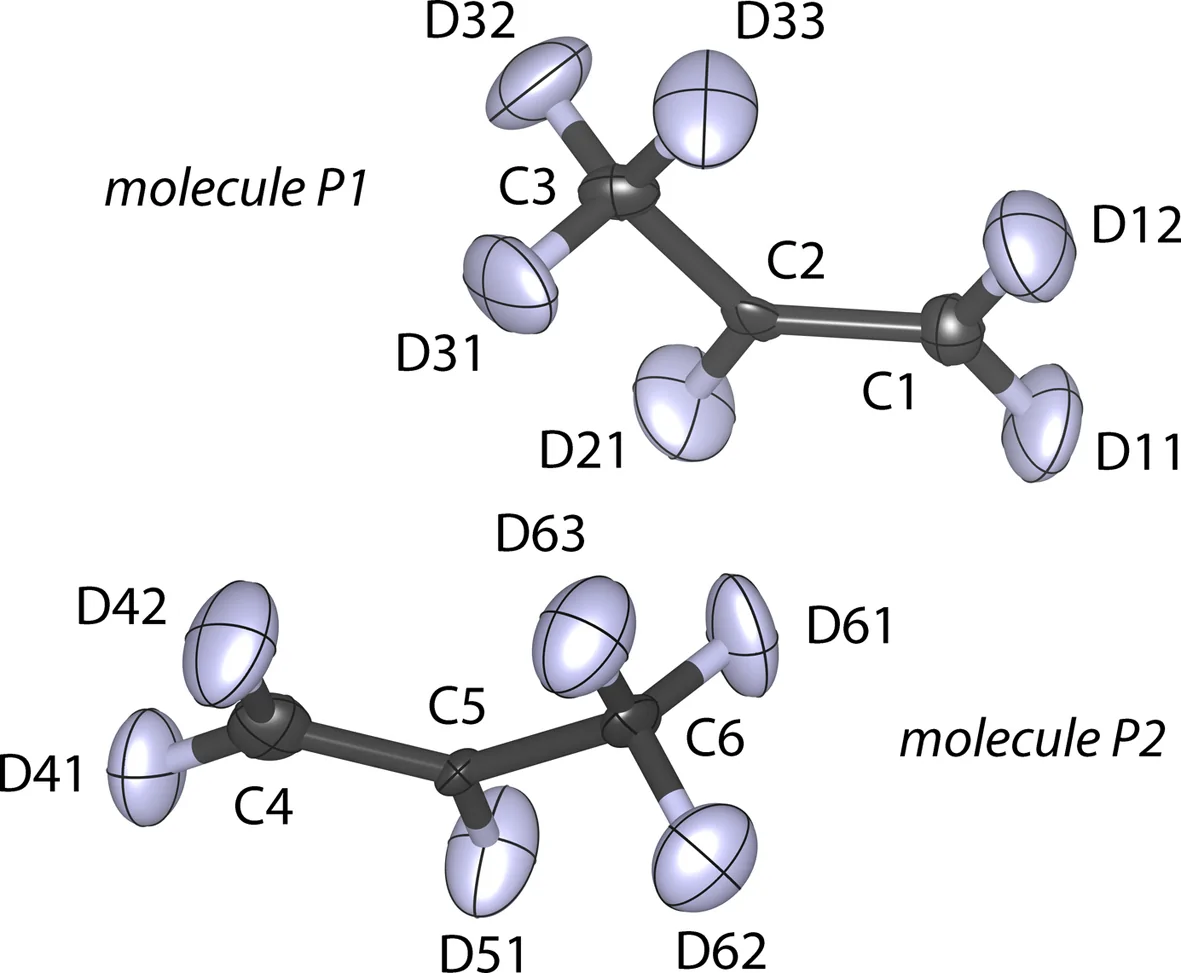
Refined geometry and anisotropic displacement parameter ellipsoids (drawn at the 75% probability level) for the two symmetry-inequivalent propene-D6 molecules in the solid state at 10 K (see Table S5 for bond lengths and angles).

The geometries of the two symmetry-inequivalent propene molecules obtained from our Rietveld refinements (Table S5, Fig. 4 ▸) agree well with the athermal DFT-based geometries, although – as with propane – the dimensions of the unit cell are substantially underestimated even by the dispersion-corrected DFT calculations.

### 4.3. A note on the possible structure of metastable propene

The *Polymorph* module in *Materials Studio* generated 638 candidate structures across the eight b.c.t. space groups searched; a plot of the total energy against density of these structures is shown in Fig. S9. There is a clear energetic preference for structures in space group 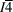, amongst which the ten lowest-energy candidates are each essentially identical in terms of their packing, with only small differences in tilt angles between neighbouring molecules, and with unit-cell parameters from 10.85 × 10.85 × 5.63 Å up to 11.38 × 11.38 × 5.20 Å. Importantly, the molecular packing in these favourable 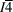 structures contains precisely the same motif of canted molecules as found in the experimental *P*2_1_2_1_2_1_ structure. After geometry optimization of the lowest-energy 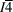 structure in *CASTEP*, an overlay of the *P*2_1_2_1_2_1_ and 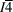 structural motifs (Fig. S10) shows remarkable agreement. The difference, as one might expect when transforming a structure with two symmetry-inequivalent molecules (*P*2_1_2_1_2_1_) to a structure with only one such molecule (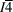), is a rotation of ∼120° around the long axis of one of the molecules (Fig. S11 shows the overlay after such a rotation has been applied).

We have used *GSAS-I* to simulate the neutron powder diffraction pattern of the 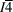 structure. For these purposes, we fixed the lattice parameters of the structure at the values obtained from our indexing of the 10 K dataset produced by Lennon *et al.* (2000 ▸) (Table S1) and carried out an optimization of the atomic coordinates in *CASTEP*. In *GSAS-I*, we used a modern HRPD instrument parameter file but adjusted the peak profile parameters σ1 and γ1 to match crudely by eye the broadened peaks in the 1998 dataset. It is apparent from this exercise that the 1998 data exhibit *hkl*-dependent line broadening, but for the purposes of our simulation we adopted a simple uniform broadening. In post-processing of the 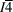 simulated diffraction pattern we also applied a degree of wavelength-dependent Gaussian noise to approximate the poor counting statistics in the 1998 dataset. The 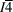 simulation is compared with the data of Lennon *et al.* (2000 ▸) in Fig. S12. While there are some significant differences in relative intensities, the overall impression is that the simulated diffraction pattern of the fully deuterated 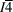 phase is a good match to the observed pattern.

The evidence we have presented strongly suggests that Lennon *et al.* (2000 ▸) observed a metastable b.c.t. form of propene with a structure either identical to or closely resembling the 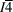 structure reported here, and that the transition from that structure to the stable *P*2_1_2_1_2_1_ structure involves nothing more than a rotation of one molecule to break the symmetry equivalence. It is interesting to note that the 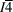 and *P*2_1_2_1_2_1_ structures relaxed in *CASTEP* differ in molar volume by only 0.337% (with the 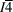 structure being the smaller of the two), whereas the experimental data show the 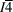 structure to be 2.3% larger in molar volume than the *P*2_1_2_1_2_1_ structure, at least up to 65 K. This difference, as shown in our discussion of density below, is within the realm of possibility for being attributable to differences in isotopic makeup. Whilst Lennon *et al.* thought their high background most likely to be due to contamination by protiated material, the large volume difference would appear to require their sample to have been fully protiated. In our experience of measuring fully protiated samples on HRPD, we consider it extremely unlikely that any Bragg peaks from the sample would have been visible in the wavelength range covered by the instrument’s 100–200 ms TOF window, simply due to attenuation from the incoherent scattering cross section of ordinary hydrogen, and the data would in any case be considerably noisier than they already are for the relatively short measurement times employed in the 1998 experiment. Furthermore, significant exchange of H for D leads to substantial differences in the structure factors, by virtue of the large contrast in coherent neutron scattering length between H (−3.739 fm) and D (6.671 fm). We have simulated the 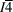 structure with varying degrees of uniformly distributed protiation (Fig. S13) and with protiation distributed non-uniformly, either on the methyl group hydrogen atoms alone, or on the combined methyl­ene and methine hydrogen atoms (Fig. S14). It is apparent that modest degrees of protiation result in large differences in the relative intensities of the Bragg peaks, most notably the intensity of the 310 reflection at 3.079 Å *d* spacing relative to the strong 211 reflection at 3.694 Å. Moreover, we can rule out substantial protium exchange concentrated on the CH/CH_2_ end of the molecule as this greatly enhances the intensity of the 130 peak (3.523 Å) and 330 peak (2.629 Å) relative to the 211 peak, whilst also suppressing the 240 peak (2.491 Å) in a fashion that is inconsistent with the observed diffraction pattern. Working on the basis that the 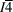 structure is correct, as other lines of evidence indicate, then these simulations show that the sample measured by Lennon *et al.* (2000 ▸) cannot have undergone a substantial degree of D/H exchange and certainly cannot have been a mis-labelled cylinder of C_3_H_6_ or a cylinder containing material with isotopically labelled functional groups.

The difference in volume might instead be attributable to rotational disorder of the propene molecules around their long axes, similar to what was observed in *n*-butane (Refson & Pawley, 1986 ▸). The predicted 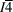 structure clearly shows that the packing is insensitive to rotations of 120° about the long axis of the P2 molecule, and the calculated energy difference between the fully relaxed *P*2_1_2_1_2_1_ and 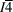 structures with these differing molecular rotations amounts to only 0.25 kJ mol^−1^. Either dynamic disorder or microscopic domains of static disorder might explain both the high background (*i.e.* diffuse scattering) and potential *hkl*-dependent size broadening of the Bragg peaks. However, the inference of rotational disorder remains to be experimentally confirmed, and it is worth tempering comparisons made between simulated and observed diffraction patterns, as well as computed spectra, with the awareness that the simulations have been carried out on the basis of a fully ordered structural model.

### 4.4. Thermal expansion of propane and propene

The unit-cell parameters of propane as a function of temperature (10.25–80 K) are given in Table S2 and plotted in Fig. S2. Those of propene (10–85 K) are provided in Table S3 and Fig. S4. The behaviour of propene is noteworthy in that there is a small but significant hysteresis in the length of the *c* axis, with this dimension expanding slightly faster on heating than it contracted on cooling, followed by evidence of a turnover or structural ‘relaxation’ on approaching the melting point.

In order to interpolate smoothly between these data and obtain some insight into the underlying vibrational characteristics of the material for comparison with the spectroscopic study, the lattice parameters of both solids were fitted with a second-order Grüneisen approximation to the zero-pressure equation of state [equation (1)]. In this approximation, the thermal expansion is considered equivalent to elastic strain such that
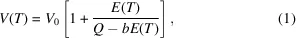
where *V*_0_ is the unit-cell volume at zero applied pressure (*i.e.* atmospheric pressure, 100 kPa), *b* = ½ (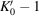) and *Q* = (*V*_0_*K*_0_/γ). *K*_0_ is the zero-pressure isothermal bulk modulus, 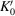 is its first derivative with respect to pressure and γ is the thermal Grüneisen parameter. The internal energy due to lattice vibrations, *E*(*T*), is then determined via a Debye model,
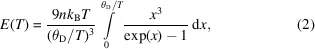
where θ_D_ is the Debye temperature, *n* is the number of atoms per molecule and *k*_B_ is the Boltzmann constant; the integral term is evaluated numerically.

So as to be dimensionally correct, the lengths of the unit-cell edges were fitted as *a*^3^, *b*^3^ and *c*^3^. The values of *K*_0_/γ reported for each axis therefore correspond with *e.g.K_a_*/γ = −*a*^3^ (d*P*/d*a*^3^). For propane, we fitted both *a*^3^ and (*a*sinβ)^3^, from which the fitted temperature dependence of β was derived. The fitted parameters are given in Tables S6 and S7. For the *c* axis of propene, where we were obliged to fit the cooling and warming curves separately and to exclude the points nearest the melting temperature, the uncertainties on the fitted parameters are inevitably quite poor, but the fits provide an adequate interpolation for the purposes of understanding the behavioural trends.

Both the refined lattice parameter values and the fitted Debye models were used to calculate unit-strain tensors (*i.e.* thermal expansion tensors) as a function of temperature with the *STAVE* program (Fortes, 2025 ▸). The resulting tensor coefficients were used to derive the eigenvalues and eigenvectors; these constitute the principal linear thermal expansivities, α_1_, α_2_ and α_3_, along three orthogonal directions, and the angles between those principal directions and the crystallographic reference frame. The eigenvalues sum to α_*V*_, the volume thermal expansion. In propene, symmetry constrains the principal directions to coincide with the crystallographic axes, but in propane only one principal direction is obliged by symmetry to be co-aligned with the two-fold axis; the other two directions may adopt any orientation. The most concise method of indicating the orientation of the principal directions in lower-symmetry crystals is graphically, with a 3D tensor representation surface, or glyph, where the variation of strain with direction is indicated by colour coding of the representation surface (Hashash *et al.*, 2003 ▸).

**Figure 5:**
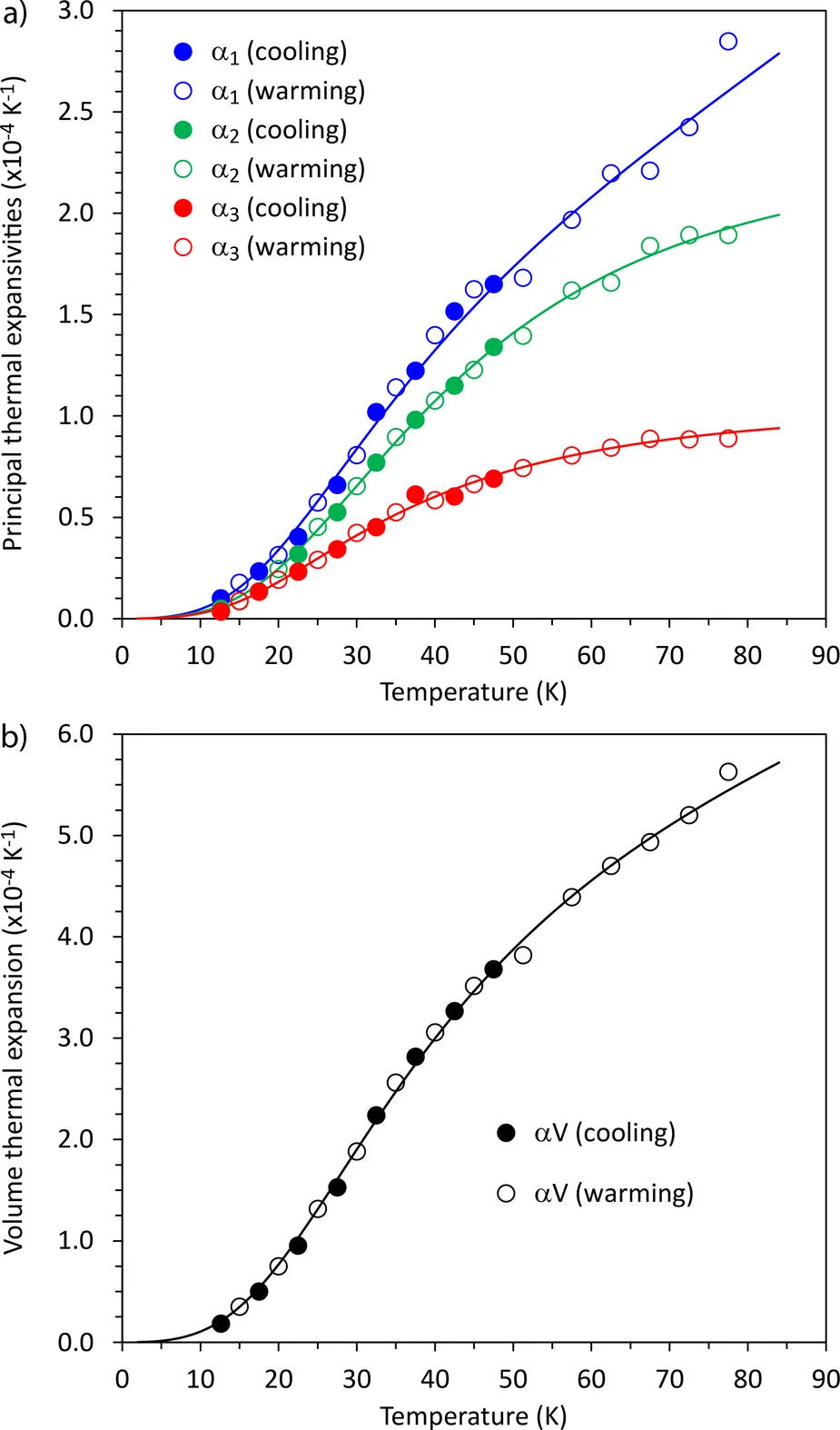
(*a*) Variation of the principal linear thermal expansivities in propane derived from the refined lattice parameter data and from the fitted Debye models (solid lines). (*b*) Variation of the volume thermal expansion coefficient of propane with temperature.

**Figure 6:**
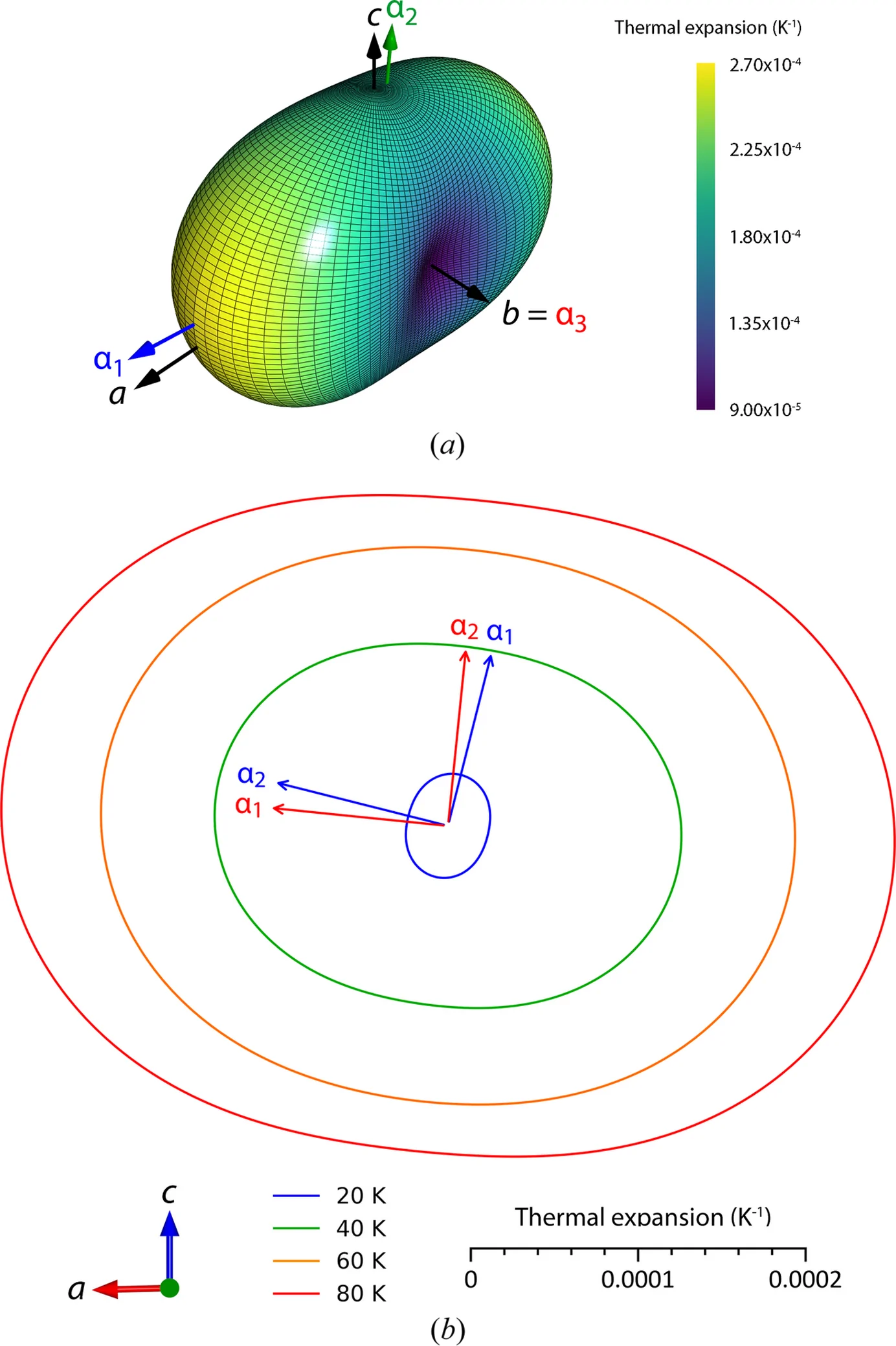
(*a*) Propane thermal expansion tensor representation surface at 80 K, indicating the principal directions α_1_ and α_2_ (α_3_ is parallel to the *b* axis by symmetry) and the magnitude of the thermal expansion with direction by colour coding of the surface. (*b*) Slices through the tensor representation surface at 20 K intervals, revealing the very slight changes in orientation of the principal directions. Note that the directions of greatest and intermediate expansion, α_1_ and α_2_, reverse at low temperature.

**Figure 7:**
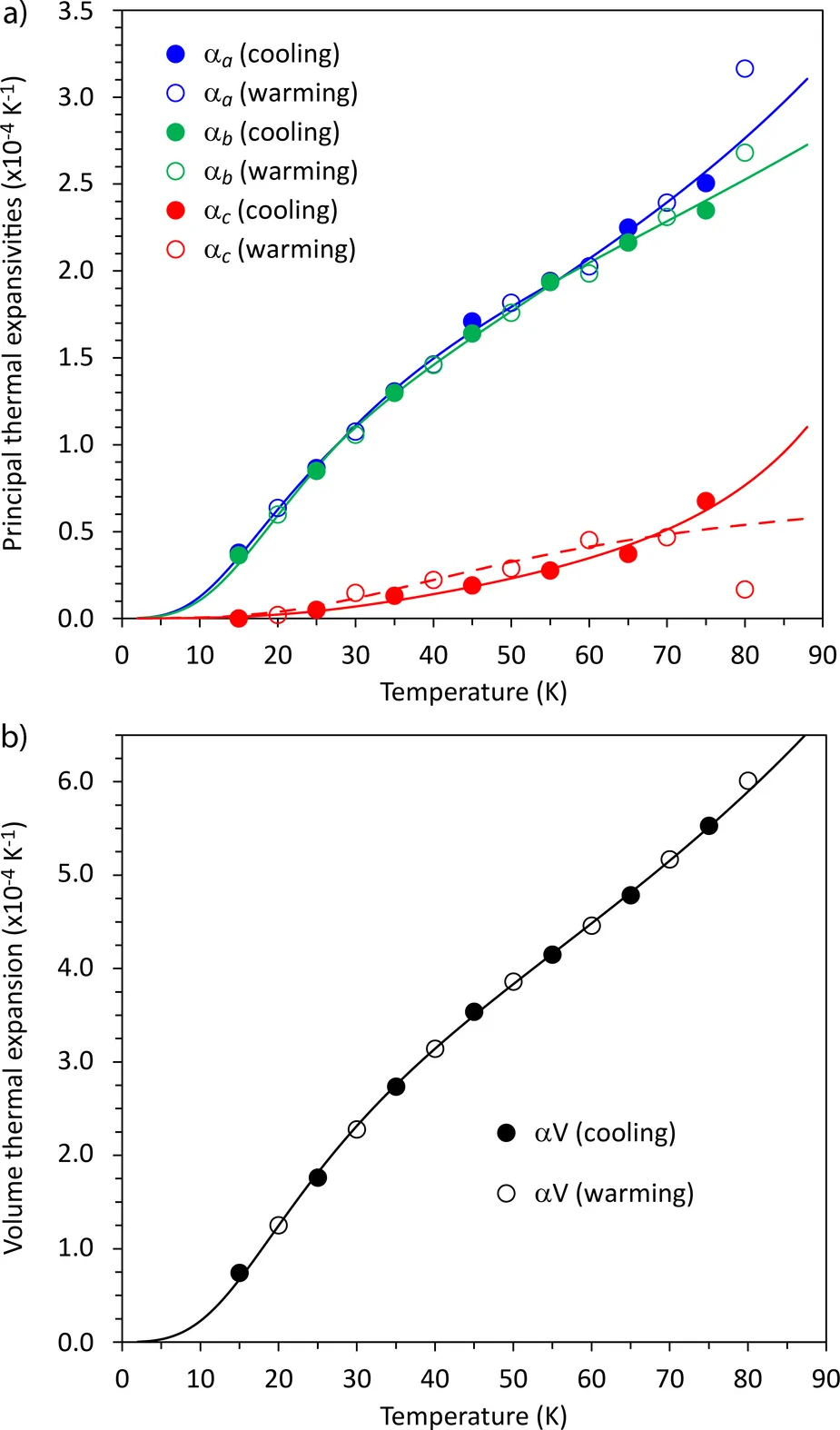
(*a*) Variation of the principal linear thermal expansivities in propene derived from the refined lattice parameter data (symbols) and from the fitted Debye models (solid/dashed lines). (*b*) Variation of the volume thermal expansion coefficient of propene with temperature.

**Figure 8:**
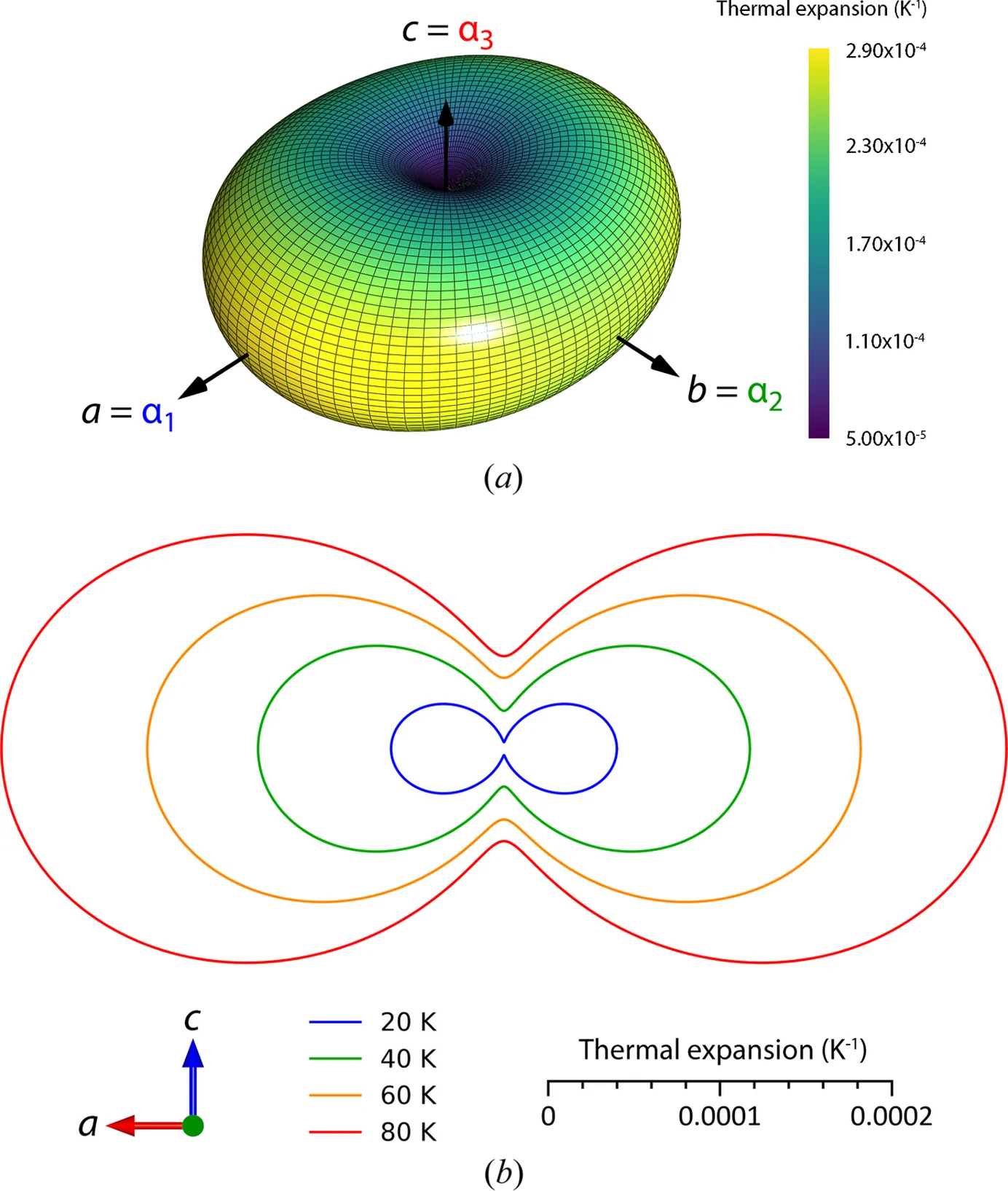
(*a*) Propene thermal expansion tensor representation surface at 80 K. The principal directions α_1_ and α_2_ are parallel to the *a* axis and *b* axis, respectively, and α_3_ is parallel to the *c* axis. The magnitude of the thermal expansion with direction is indicated by colour coding of the surface. (*b*) Slices through the tensor representation surface at 20 K intervals.

Fig. 5 ▸ reports the magnitudes of the principal thermal expansivities and volume thermal expansion in propane, and Fig. 6 ▸ depicts the thermal expansion tensor representation surface at 80 K, with discrete slices perpendicular to the two-fold axis at 20, 40, 60 and 80 K to indicate changes in the orientation of the principal directions. Similarly, Fig. 7 ▸ shows the principal and volumetric thermal expansivities of propene, whilst Fig. 8 ▸ depicts the thermal expansion tensor representation and slices in 20 K increments.

**Figure 9:**
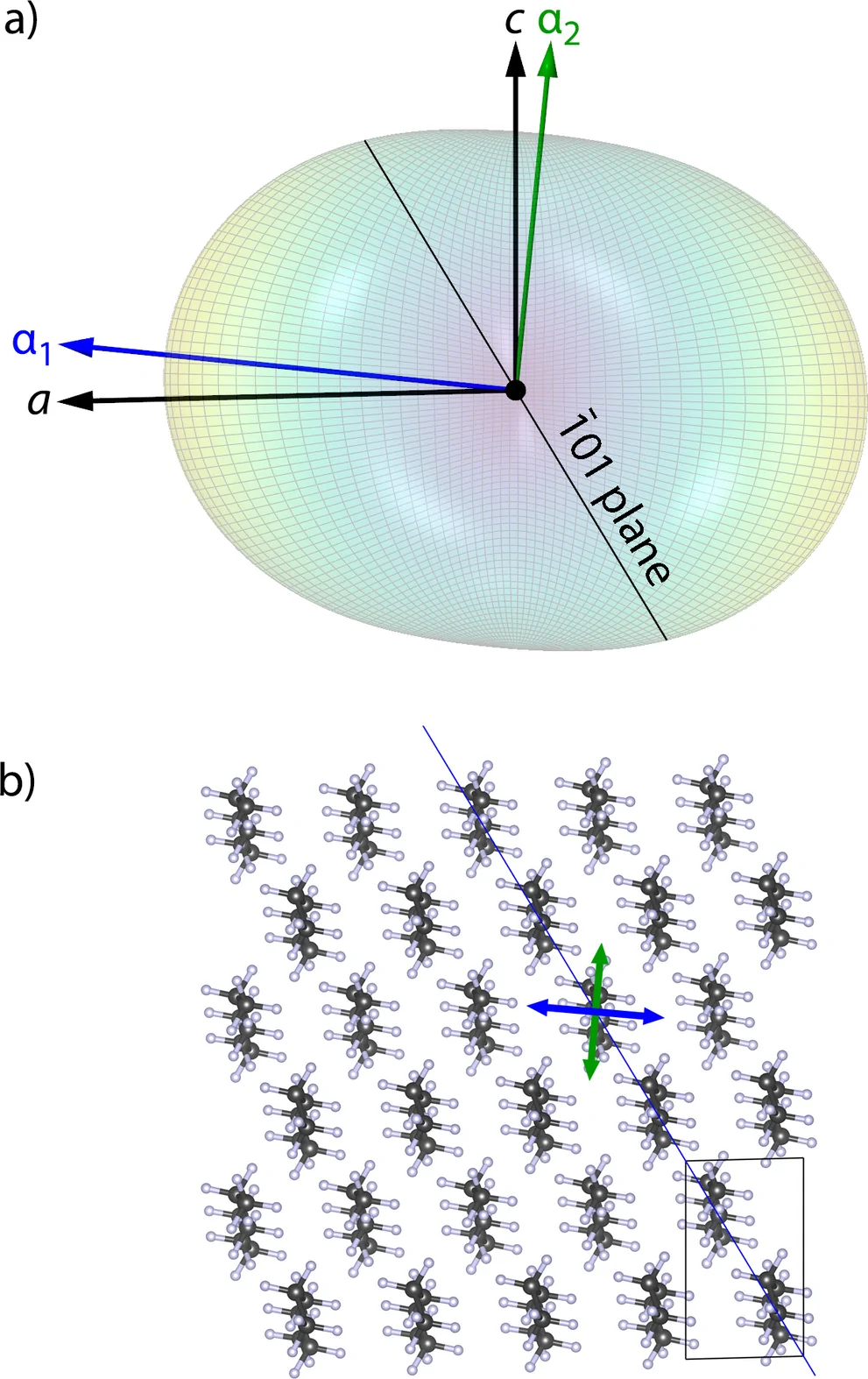
(*a*) View of the 80 K thermal expansion representation surface for propane, showing the alignment of the 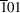 plane corresponding with the pseudo-close-packed sheets in the structure, shown in (*b*). Arrows indicate the orientation of α_1_ (blue) and α_2_ (green) overlaid on the molecular packing, highlighting the proximity of the nearest-neighbour molecules along α_1_.

Propane’s thermal expansion is moderately anisotropic. The direction of least thermal expansion is along the *b* axis, which as noted earlier corresponds to the wavevector of the undulations in the pseudo-close-packed sheets. However, the direction of greatest thermal expansion does not lie perpendicular to the sheets (*i.e.* perpendicular to the 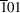 plane), as shown in Fig. 9 ▸(*a*). An examination of the sheet’s stacking shows that the direction of maximum thermal expansion is associated with the shortest interplane contact with the lowest interaction energy [Fig. 9 ▸(*b*)].

Propene’s thermal expansion is considerably more anisotropic, which seems surprising since it lacks a layered structure. The thermal expansions of the *a* and *b* axes are essentially identical, while being substantially greater than the expansion of the *c* axis, and this near uniaxial behaviour reflects what we believe to be the underlying high-symmetry tetragonal aristotype that appears to have been manifested in the earlier study of Lennon *et al.* (2000 ▸). The slices through the tensor representation surface [Fig. 8 ▸(*b*)] clearly show that there is no significant change in the expansion behaviour with temperature.

The maximum volume thermal expansion of both propane and propene is ∼6 × 10^−4^ K^−1^ close to their respective melting points. This is considerably smaller than the thermal expansions of both methane and ethane at 85 K, for which α_*V*_ ≃ 1.4 × 10^−3^ K^−1^ and 1.8 × 10^−3^ K^−1^, respectively (Aadsen, 1975 ▸; Klimenko *et al.*, 2008 ▸). It is noteworthy that the thermal expansion of propane saturates on cooling at a higher temperature than that of propene, with the consequence that the fitted Debye temperatures differ significantly. For propane, θ_D_ obtained from fitting the unit-cell volume is ∼166 K, and for propene it is ∼110 K, values that correspond to frequency cut-offs of ∼115 cm^−1^ and ∼80 cm^−1^, respectively. As shown in the later discussion of the vibrational spectra, the observed high-frequency edge of the external modes is found at ∼119 cm^−1^ in propane-D8 and at ∼108 cm^−1^ in propene-D6. While the absolute values obtained from the Debye model fit are thus in reasonable agreement with the INS results, and the difference between propane and propene is in the expected sense, the magnitude of the difference is much smaller than expected. This is probably due to the simplified nature of the Debye model.

### 4.5. The densities of propane and propene

As noted in the *Introduction*[Sec sec1], we can (in principle) use the lattice parameters of the perdeuterated crystals to estimate the densities of the protiated solids, either for comparison with the work of others or to understand the physical properties of these solids in nature; for example, to determine the buoyancy of propane ice in Titan’s hydro­carbon rivers and seas. But these estimates ignore one critical factor, the effect of isotopic substitution on the molar volume, of which the most significant effect by far is the substitution of hydrogen by deuterium. Volume isotope effects (VIE) are extremely sparsely characterized in hydrogen-bearing molecular crystals, with much of the work being done on very simple and abundant species, such as water ice and solid methane, for example, with relatively few studies of organic crystals where deuteration has produced significant changes in structural packing or thermo­physical properties [see *e.g.* Kupka *et al.* (2012 ▸), Merz & Kupka (2015 ▸) and Rejnhardt *et al.* (2023 ▸)]. Volume isotope effects are usually rather small, can vary in sign and magnitude with temperature (and probably pressure), and require care to be measured with good accuracy and precision. When one is using neutron powder diffraction in particular, there is a pressing need to suppress the incoherent scattering from natural hydrogen by working with perdeuterated samples, as we did here, and it is not common to measure protiated analogues for comparison without a good reason to do so. The calculation of VIEs from first principles does not – yet – have a good track record of accuracy.

The VIE in the ambient pressure phase of water ice is famously ‘anomalous’, being of opposite sign to that expected on the basis of elementary theory, such that the unit-cell volume of D_2_O ice I*h* is larger than that of H_2_O ice I*h* (*i.e.* the density is lower) by ∼0.05% below 100 K, rising to ∼0.25% near the melting point (Fortes, 2018 ▸). By contrast, in benzene, the VIE is ‘normal’, with C_6_D_6_ denser than C_6_H_6_ at a given temperature by an almost temperature-invariant 0.5% (Fortes & Capelli, 2018 ▸). Perhaps the most appropriate comparison for the present study is methane, where there have been a number of crystallographic studies of both CH_4_ and CD_4_ (Aadsen, 1975 ▸; Baer *et al.*, 1978 ▸; Prokhvatilov & Isakina, 1983 ▸). These show that the VIE in methane is normal and that it is both large in magnitude and varies considerably with temperature, being ∼3% at limiting low temperatures and dropping to ∼0.5% near the melting point, although the differences at low temperatures appear to be magnified by a series of polymorphic phase transitions.

We can apply basic thermodynamic relations to estimate the sign and magnitude of the VIE in propane and propene and compare the results with available density data for the protiated solids of each species. The densities of both liquid propane and liquid propene have been determined as a function of temperature (Huang, 1966 ▸; Glos *et al.*, 2004 ▸). From these data we can obtain accurate values for the liquid densities at their respective melting points, ρ_M_(liq) = 732.45 kg m^−3^ at 85.5 K for propane and ρ_M_(liq) = 767.70 kg m^−3^ at 88.0 K for propene. The density of the solid at the melting point is then derived from the change in volume upon melting, Δ*V*_M_, via the Clausius–Clapeyron equation
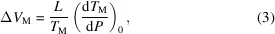
where *L* is the enthalpy of fusion and (d*T*_M_/d*P*)_0_ is the zero-pressure Clapeyron slope of the pressure melting curve.

The fusion enthalpies of propane and propene are 3500 J mol^−1^ and 3002 J mol^−1^, respectively (Pavese & Besley, 1981 ▸; Powell & Giauque, 1939 ▸). The high-pressure melting curves of both solids were reported by Reeves *et al.* (1964 ▸). Their work provides coefficients from the fitting of a Simon–Glatzel equation to the observations, but plotting the pressure melting temperatures using the tabulated values yields curves for propane and propene with very similar zero-pressure slopes, which differs markedly from what is illustrated in Fig. 2 of Reeves *et al.* (1964 ▸). We therefore extracted values from this figure by graphical methods and fitted Simon–Glatzel equations of our own to derive the most accurate possible Clapeyron slopes. We thus obtain (d*T*_M_/d*P*)_0_ = 9.78 × 10^−8^ K Pa^−1^ for propane and 6.36 × 10^−8^ K Pa^−1^ for propene.

**Figure 10:**
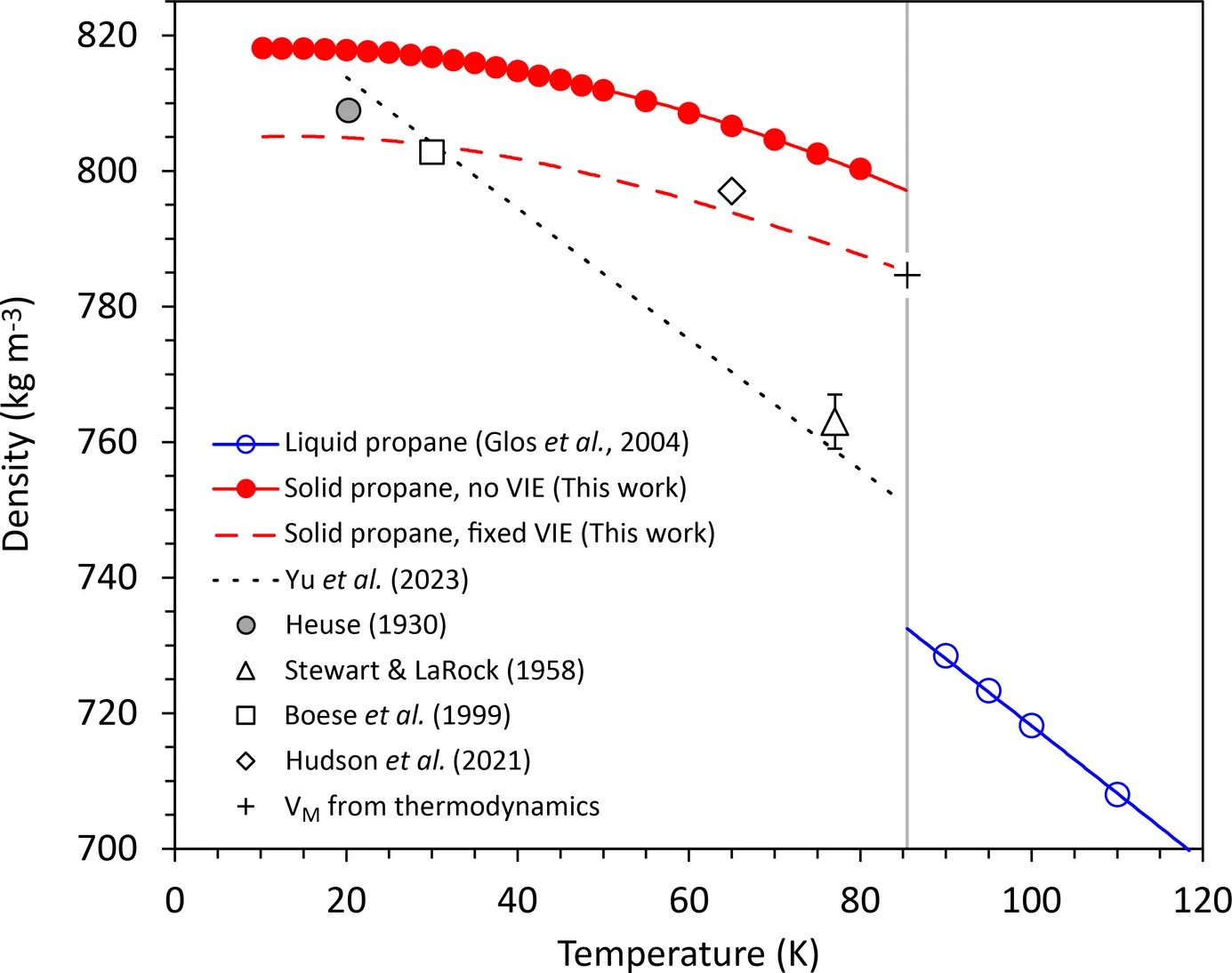
Temperature dependence of the density in ordinary propane.

From the available values, we therefore derive Δ*V*_M_ = 9.0789 × 10^−5^ m^3^ kg^−1^ and ρ_M_(cryst) = 784.62 kg m^−3^ for propane, yielding a ‘normal’ volume isotope effect of 1.585%. This is comparatively large, but well within the range exhibited by solid methane. If we simply use our C_3_D_8_ molar volumes and increase them by 1.585% to reduce the density, we find that our ‘corrected’ values now pass between the two relatively recently published data points for the density of solid propane [Boese *et al.* (1999 ▸) and Hudson *et al.* (2021 ▸)] and agree tolerably well with the results from ‘Preparation 2’ of Heuse (1930 ▸), which he observed had the highest degree of purity. By contrast, the agreement with measurements made at the boiling point of liquid nitro­gen by Stewart & La Rock (1958 ▸) is very poor. Yu *et al.* (2023 ▸) used the available measurements, excluding Hudson *et al.* (2021 ▸) as far as we can tell, to derive a linear parameterization of the density of solid propane for planetary applications, which yields a temperature dependence very similar to that of the liquid and a small Δ*V*_M_. They report a density value of 813.7 kg m^−3^ from Heuse (1930 ▸), which does not appear to be the mean of his four reported values (815.5 kg m^−3^) and ignores his stated confidence in the higher-purity samples with a lower density (808.4 ± 0.7 kg m^−3^) at the boiling point of hydrogen. As a result, the parameterization from Yu *et al.* stands in sharp disagreement with the highly accurate and precise thermal expansivity we obtain and with the thermodynamically obtained value of Δ*V*_M_. Our applied density correction is clearly accurate to some degree, as well as being thermodynamically self-consistent. In the absence of any other supporting data, which might be used to indicate some temperature dependence of the VIE, then the corrected curve shown in Fig. 10 ▸ should be used instead of the expression given by Yu *et al.* to estimate the density of solid C_3_H_6_ via a simple cubic polynomial fit, ρ(*T*) = 2.0496 × 10^−5^ *T*^3^ − 6.1779 × 10^−3^ *T*^2^ + 1.5744 × 10^−1^ *T* + 804.05, where *T* is in kelvin and ρ(*T*) is in units of kg m^−3^.

**Figure 11:**
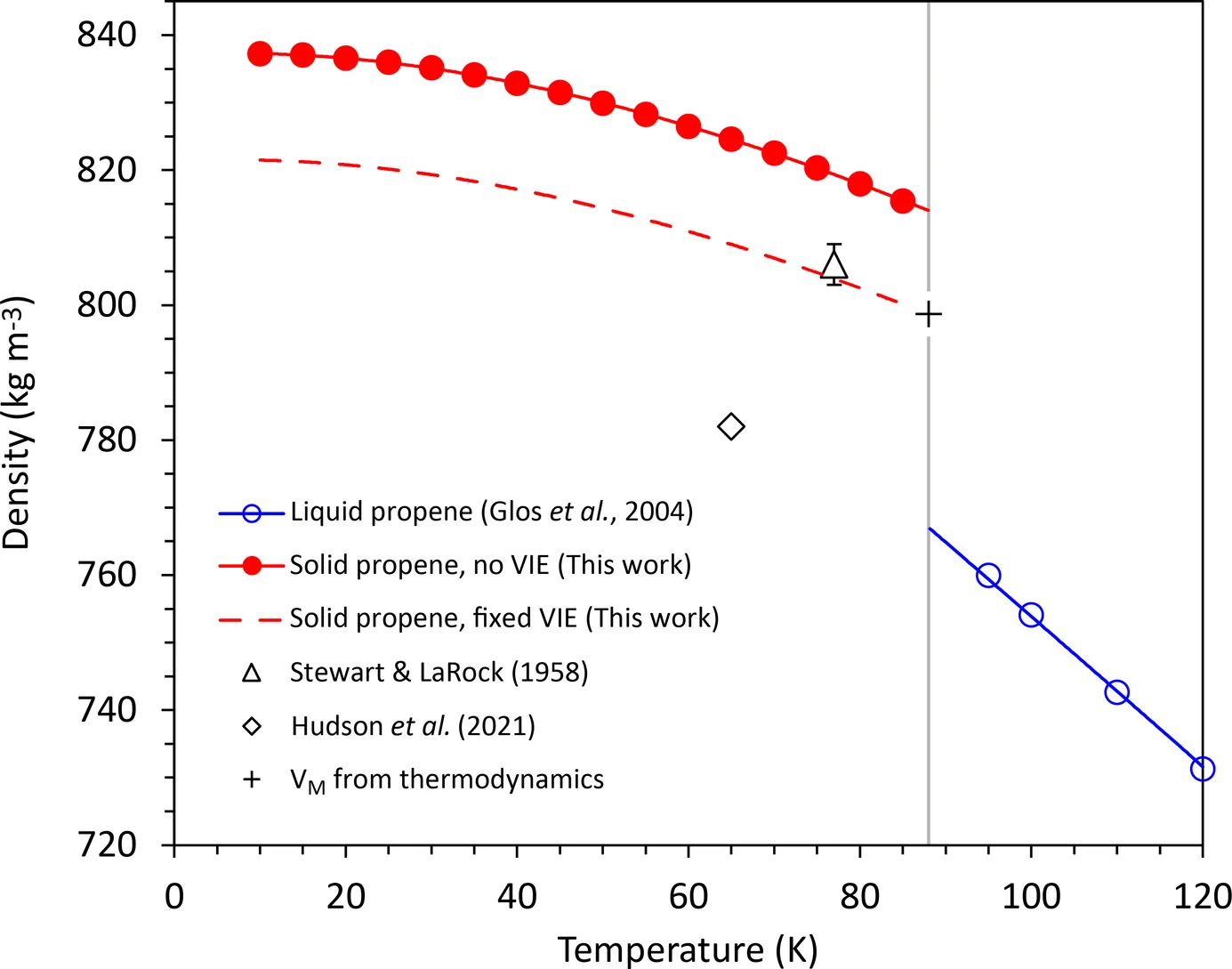
Temperature dependence of the density in ordinary propene.

Likewise for propene, we derive Δ*V*_M_ = 5.1566 × 10^−5^ m^3^ kg^−1^ and ρ_M_(cryst) = 798.66 kg m^−3^, yielding a ‘normal’ volume isotope effect of 1.886%. Again, this is relatively large, but not unprecedented. Increasing our C_3_D_6_ molar volumes by 1.886% gives the ‘corrected’ density curve shown in Fig. 11 ▸. The available density data for solid propene are very few and far between, with only two known values (Stewart & La Rock, 1958 ▸; Hudson *et al.*, 2021 ▸). The value of 782 kg m^−3^ at 65 K from Hudson *et al.* (2021 ▸) is clearly anomalously low; indeed, it appears to be lower than the density of the liquid phase extrapolated to this temperature. However, the older value from Stewart & La Rock (1958 ▸) agrees well with our corrected density curve. Since those authors found the density of ethane at 77 K within 1% of the value later obtained by X-ray diffraction (Klimenko *et al.*, 2008 ▸), we judge that their methodology produced accurate results and attribute their low propane density to issues with solidification that they documented in their paper. With so few literature data, we cannot further judge the accuracy of our corrected curve, but present it simply as the best possible estimate of the density of solid C_3_H_6_ for the time being. As above for propane, the density of propene can be computed via a cubic polynomial fit, ρ(*T*) = 9.8509 × 10^−6^ *T*^3^ − 4.4513 × 10^−3^ *T*^2^ + 5.7762 × 10^−2^ *T* + 821.34, where *T* is in kelvin and ρ(*T*) is in units of kg m^−3^.

### 4.6. INS spectroscopy of propane-H8/D8

**Figure 12:**
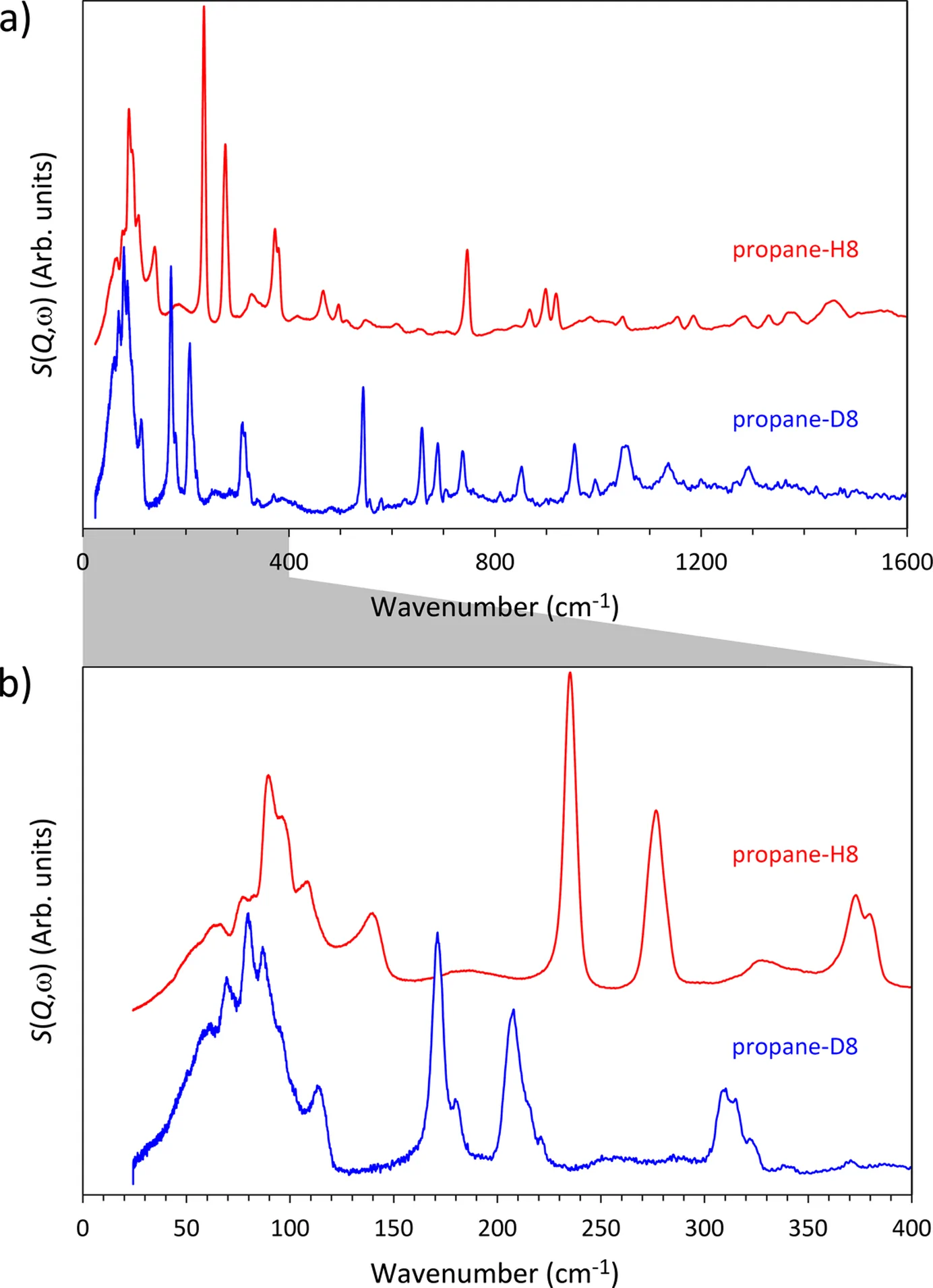
INS spectra of propane-H8 and propane-D8. Panel (*a*) shows the complete 0–1600 cm^−1^ range and panel (*b*) shows an expanded view of the external modes and the low-energy internal modes.

Fig. 12 ▸ shows the measured INS spectra of propane-H8 and propane-D8. The spectrum of propane-H8 matches that of Nelligan *et al.* (1987 ▸) but our data have a higher resolution and extend beyond 1000 cm^−1^. The spectrum of propane-D8 has not previously been reported. The deuterated spectrum shows the expected redshift due to isotopic substitution, but the features above 500 cm^−1^ are relatively more intense than in the H8 data, even though the overall signal-to-noise ratio is poorer. Since the neutron scattering cross section of deuterium is only 9% that of protium, then the same quantity of material in the beam leads to a lower overall intensity and a reduced signal-to-noise ratio. Conversely, the increased mass of C_3_D_8_*versus* C_3_H_6_ will result in a smaller Debye–Waller factor, and so the high-energy modes are less damped in the deuterated species, resulting in the greater observed intensity at higher wavenumbers.

The present work has confirmed the stable structure of propane in the solid state to be monoclinic, space group *P*2_1_/*n* (No. 14), *Z* = 4, with all of the molecules on sites of *C*_1_ symmetry. The highest possible symmetry of the propane molecule is *C*_2*v*_ and the correlation table (Table S8) shows that each mode in the free molecule correlates to four modes in the crystal (factor group splitting). This is apparent in Fig. 12 ▸ in the low-energy region, especially for propane-D8, where several of the modes exhibit clearly resolved splitting or distinct shoulders.

DFT calculations based on the primitive unit cell provide assignments for all of the modes. Fig. S15 shows the results of *CASTEP* calculations of the INS spectrum over the entire Brillouin zone for both propane-H8 and propane-D8. The agreement is essentially quantitative. The calculated dispersion curves for propane-H8 and propane-D8 are reported in Figs. S16 and S17, respectively, with a depiction of the Brillouin zone path in Fig. S18. Apart from the acoustic trans­lational modes – which go to zero at the Γ point, (0,0,0) – the modes are quite flat, and the factor group splitting is small in most cases. This is consistent with the sharp modes seen in the INS spectra. INS is sensitive to modes at all wavevectors across the Brillouin zone (a consequence of the neutron having significant mass) and the INS spectrum can be considered as being proportional to the projection of the dispersion curves onto the energy axis.

The lowest-frequency internal modes are the methyl group’s torsional motion at 235 and 277 cm^−1^ (H8) and 171 and 208 cm^−1^ (D8), below which are the translations and librations. Inspection of the mode animations allows these to be assigned: the librations are the higher-energy features at 90 (80), 108 (93) and 140 (114) cm^−1^ (D8 values in brackets) and the translations are found in the 0–84 (75) cm^−1^ region.

**Figure 13:**
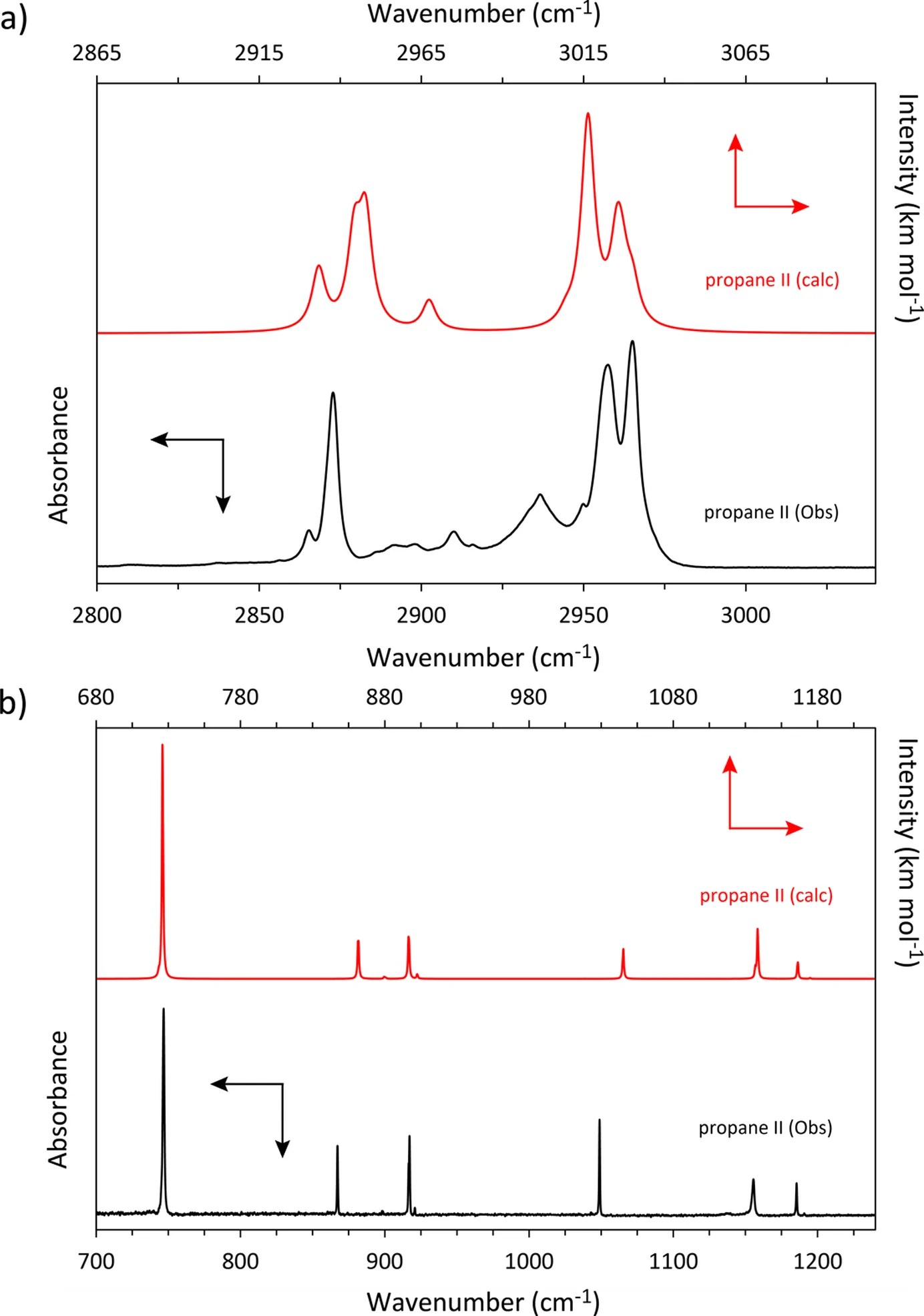
Comparison of the calculated (this work) and observed (Hudson *et al.*, 2021 ▸) IR spectra of propane-H8 in phase II. (*a*) The C—H stretch region and (*b*) the fingerprint region. Arrows indicate the pertinent horizontal and vertical axes from which to read for each displayed set of data. Note that the wavenumbers of the computed spectra are offset slightly from those of the observed data to provide visual congruity.

Fig. 13 ▸ compares the calculated IR spectrum in the C—H stretch and fingerprint regions with that measured experimentally (Hudson *et al.*, 2021 ▸) for the stable β-phase (phase II) of propane at 60 K. The agreement is excellent, confirming their assignment.

### 4.7. INS spectroscopy of propene-H6/D6

**Figure 14:**
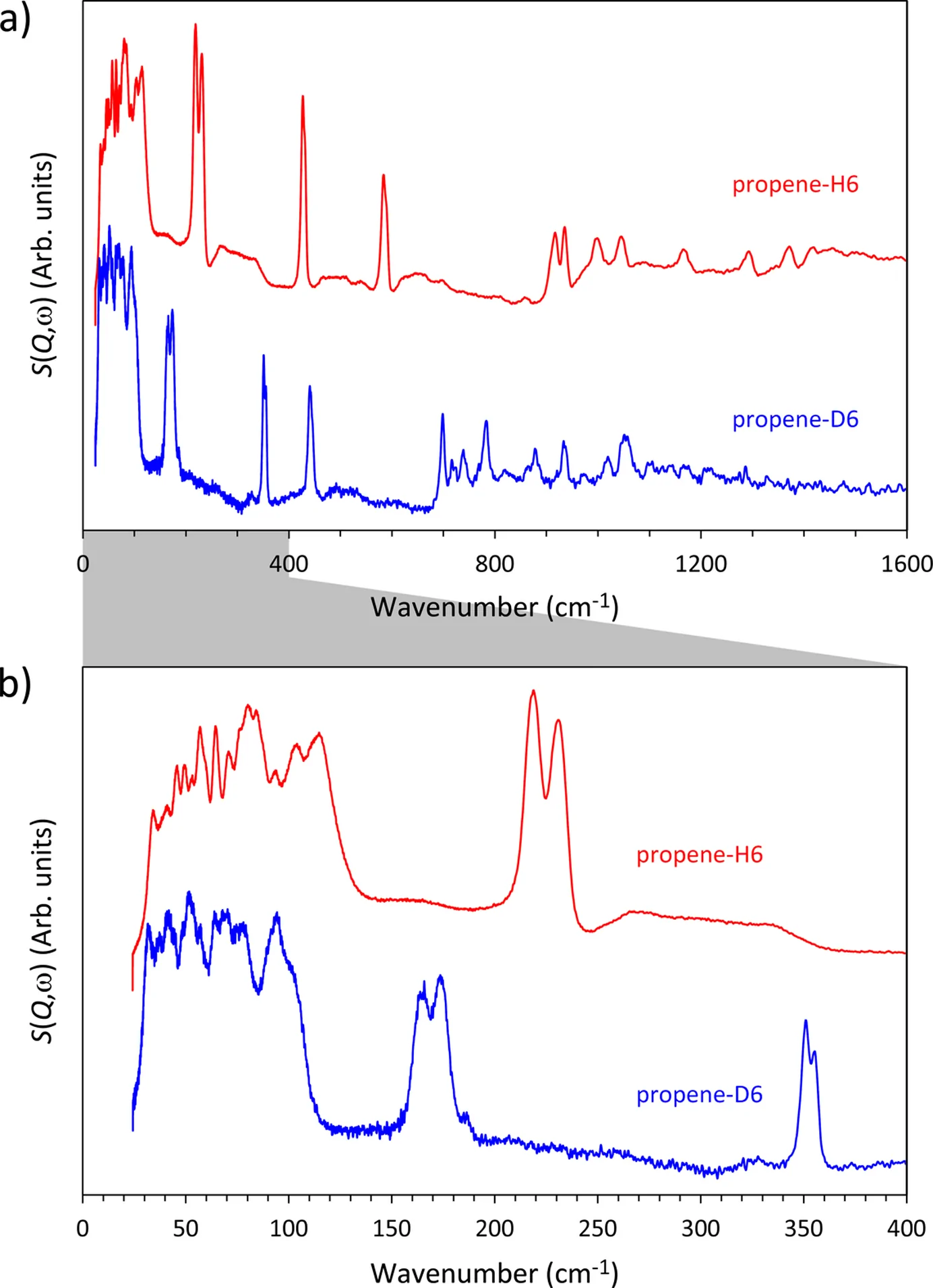
INS spectra of propene-H6 and propene-D6. Panel (*a*) shows the 0–1600 cm^−1^ range and panel (*b*) shows an expanded view of the external modes and the low-energy internal modes.

Fig. 14 ▸ shows the INS spectra of propene-H6 and propene-D6. The spectrum of propene-H6 matches that of Lennon *et al.* (2000 ▸) but with somewhat better resolution from the modern upgraded TOSCA instrument with respect to TXFA. The spectrum of propene-D6 has not previously been reported. The deuterated spectrum shows the expected redshift due to isotopic substitution and, as with propane-H8/D8, the features above 500 cm^−1^ are relatively more intense in the D6 data than the H6 data, for the same reasons as outlined above.

The present work has established the stable phase of propene in the solid state to be orthorhombic, space group *P*2_1_2_1_2_1_ (No. 19), *Z* = 8, with all of the molecules on sites of *C*_1_ symmetry. The highest possible symmetry of the propene molecule is *C*_*s*_ and the correlation table (Table S9) shows that each mode in the free molecule correlates to four modes in the crystal (factor group splitting). This is particularly apparent in Fig. 14 ▸(*b*) for the methyl torsion, where two well resolved bands are seen at 218 and 231 cm^−1^ in the H6 isotopomer and at 163 and 173 cm^−1^ in the D6 species. As noted previously by Lennon *et al.* (2000 ▸), there is only one methyl torsion in propene and it is non-degenerate, so the split band clearly indicates that there is more than one molecule in the primitive cell.

**Figure 15:**
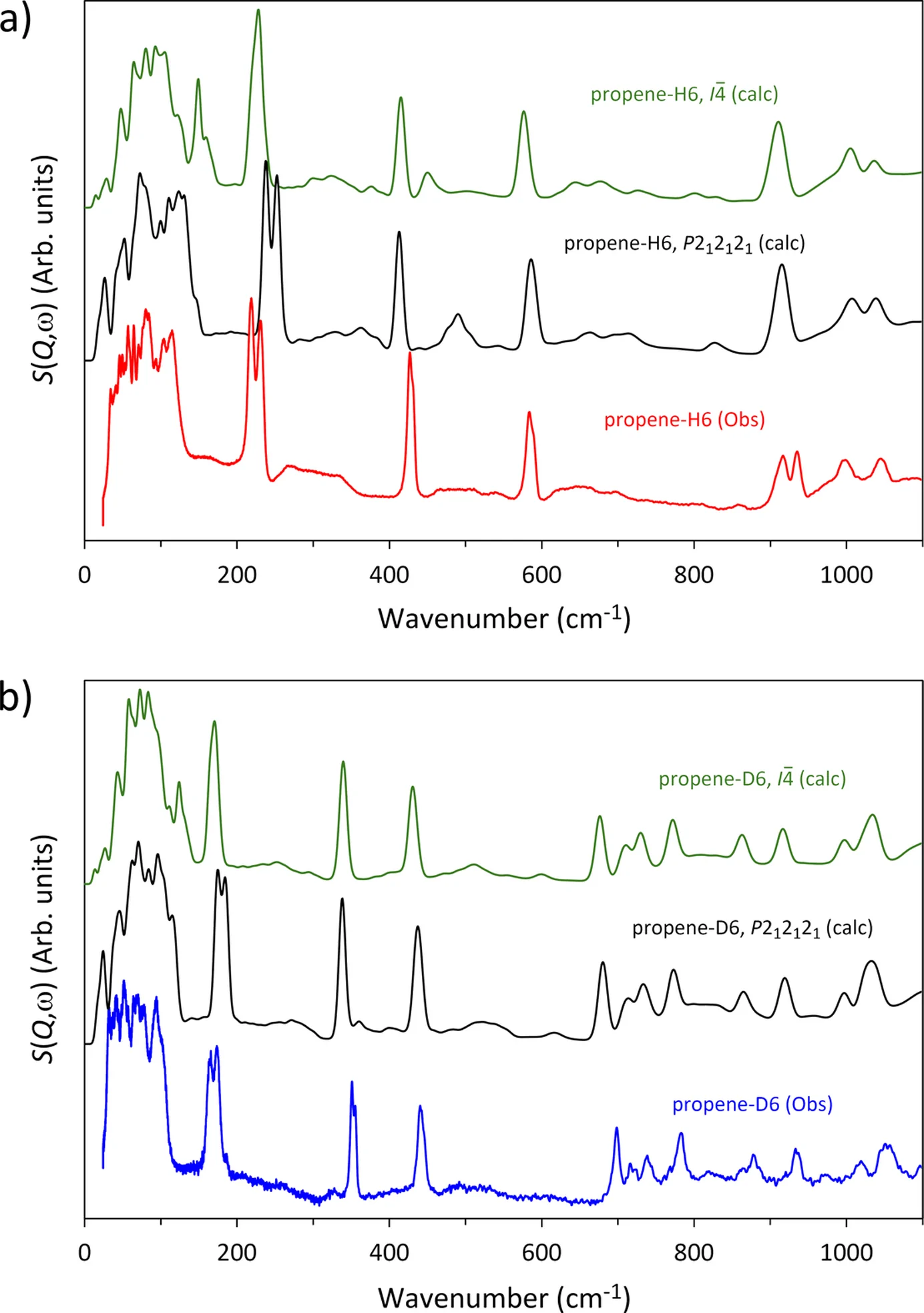
Comparison of the INS spectra measured on TOSCA with those calculated from first principles with *CASTEP* and *AbINS* for (*a*) propene-H6 and (*b*) propene-D6 in both the *P*2_1_2_1_2_1_ and 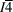 phases.

Hudson *et al.* (2021 ▸) identified a metastable phase of propene that transforms irreversibly to the stable phase at ∼70 K. It is tempting to identify this metastable phase with the 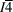 phase that we deduced earlier was observed in the previous work on propene-D6 by Lennon *et al.* (2000 ▸). The correlation table (Table S10) shows that each mode in the free molecule correlates to three modes in the crystal. Comparison of the calculated INS spectra of the *P*2_1_2_1_2_1_ and 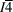 phases (Fig. 15 ▸) shows unambiguously that we have obtained the former in the present work. There are several notable features. Above 500 cm^−1^ the calculated spectra of the two phases are essentially identical and match extremely well with the experimental spectrum, apart from the splitting of the mode at 920 cm^−1^ in the propene-H6 spectrum (extended-range spectra of the H6 species are shown in Fig. S19). Below 500 cm^−1^, however, the calculated spectra of the two phases are distinctly different. In particular, the methyl torsion is manifested as one broadened band rather than two clear peaks, and one of the librations about (approximately) the C—C=C axis has moved to higher energy in the 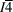 phase. We note that, at 0 K, the calculations show the two phases to be isoenergetic (within the accuracy of DFT) and both exhibit all real modes across the entire Brillouin zone (Figs. S20 and S21).

**Figure 16:**
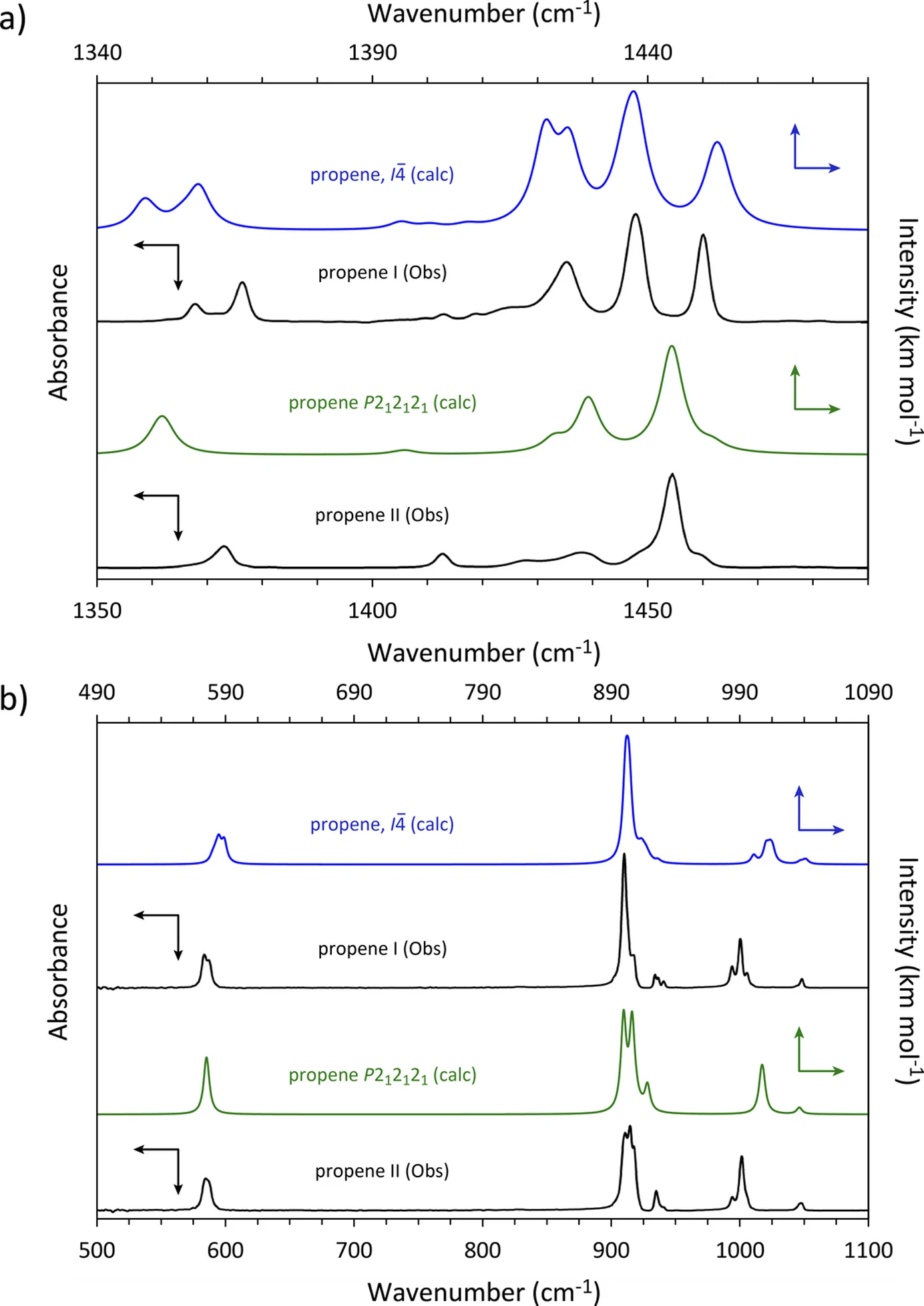
Comparison of calculated (this work) and experimental (Hudson *et al.*, 2021 ▸) IR spectra of propene-H6, illustrating good agreement between the spectrum of the 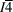 phase and propene-I, and between the *P*2_1_2_1_2_1_ phase and propene-II. Arrows indicate the pertinent horizontal and vertical axes from which to read for each displayed set of data. Note that the wavenumbers of the computed spectra are offset slightly from those of the observed data to provide visual congruity.

Fig. 16 ▸ compares the experimental IR spectra of Hudson *et al.* (2021 ▸) with those calculated for the *P*2_1_2_1_2_1_ and 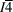 phases. It is apparent that there is a very good match for the two sets of spectra, strongly supporting the assignment of Hudson *et al.*’s metastable phase I to the 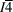 phase we derive from the data of Lennon *et al.* (2000 ▸), and their stable phase II with the *P*2_1_2_1_2_1_ structure that we measured in the present work.

## 5. Conclusions

We have reported the first refinement of the structure of crystalline propane using neutrons, affording a higher-precision structure determination than existed hitherto, and providing a highly accurate and precise determination of the solid’s thermal expansion. After correcting for an estimated volume difference between the deuterated and protiated isotopologues, we also provide a parameterization of the density of natural solid propane that agrees well with the sparse literature data and offers a straightforward means of computing the buoyancy of solid propane haze particles in Titan’s liquid hydro­carbon lakes and rivers, for example. Our spectroscopic measurements and simulations show that the monoclinic phase we observed corresponds with the stable phase II (or β-propane) of Hudson *et al.* (2021 ▸). The structure of propane-I, or α-propane, remains unknown.

Whilst this is not the first crystallographic study of propene with neutrons, it is the first to determine the structure correctly, finding a solution in space group *P*2_1_2_1_2_1_. The earlier study of deuterated propene by Lennon *et al.* (2000 ▸) reported an incorrect monoclinic indexing of the unit cell, and we have determined that their data are more consistent with a body-centred tetragonal unit cell of dimensions very similar to our *P*2_1_2_1_2_1_ phase, with a predicted structure having essentially identical molecular packing. This higher-symmetry form of propene appears likely to be a disordered metastable phase produced by devitrification of an amorphous vapour-deposited precursor.

Our phonon calculations show good agreement between the simulated IR spectrum of the *P*2_1_2_1_2_1_ phase and the stable phase II of propene (or β-propene) reported by Hudson *et al.* (2021 ▸), and between the simulated IR spectrum of the 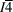 phase and the metastable phase I of propene (or α-propene). This demonstrates the merits of revisiting older diffraction data using contemporary computational methods, where indexing or structure solutions are in doubt, or where the experiment was deemed unsuccessful or only partially successful. In this instance we were able to determine the structure and properties of the metastable phase of propene with a high degree of confidence from existing datasets without ourselves making any further measurements.

As we did for propane, we obtained a highly accurate and precise determination of solid propene’s thermal expansion. Again, after correcting for the estimated volume difference between the deuterated and protiated isotopologues, we provide a parameterization of the density of natural solid propene as a function of temperature, although the literature data with which to compare our estimate are even more sparse than for propane.

## Supplementary Material

Crystal structure: contains datablock(s) PropaneD8at10K, PropeneD6at10K, PropeneD6at80K. DOI: 10.1107/S2052252526007797/fc5089sup1.cif

Supplementary figures and tables. DOI: 10.1107/S2052252526007797/fc5089sup2.pdf

Archived raw data from the neutron powder diffraction measurements: https://doi.org/10.5286/ISIS.E.RB2220007

Archived raw data from the inelastic neutron scattering measurements: https://doi.org/10.5286/ISIS.E.RB2220024

CCDC references: 2584085, 2584086, 2584087

## Footnotes

1 Boese *et al.* (1999 ▸) incorrectly gave the density at 30 K based on their lattice parameters as 743 kg m^−3^, and this has been corrected here.
