# Supplementary material for: Structure and spectroscopy of propane and propene in the solid state from neutron powder diffraction and inelastic neutron scattering
Source: IUCrJ. 2026 Sep 11;13(Pt 6):689–704. doi: 10.1107/S2052252526007797 (PMC13763904; doi:10.1107/S2052252526007797)
Supplement: Supplementary file 1 [file m-13-00689-sup2.pdf]

IUCrJ

Volume 13 (2026)

Supporting information for article:

Structure and spectroscopy of propane and propene in the solid state from neutron powder diffraction and inelastic neutron scattering

A. Dominic Fortes, Christopher M. Howard and Stewart F. Parker

Table S1

Comparison of unit-cell indexing and densities of propene obtained in various studies to date.

	Primitive monoclinic indexing		Body-centred tetragonal indexing		
Temperature	10 K[1]	65 K[1]	10 K[2]	65 K[2]	65 K[3]
a (Å)	7.2318(11)	7.2764(11)	11.1411(9)	11.1979(10)	–
b (Å)	4.9295(2)	4.9577(2)	11.1411(9)	11.1979(10)	–
c (Å)	6.4809(13)	6.5086(13)	5.5061(5)	5.5313(5)	–
β (°)	109.965(5)	109.601(5)	90.0	90.0	–
V (Å^3)	217.154	221.919	683.44	693.59	–
Z	2	2	8	8	–
ρ (kg m^{-3})	643.6†	629.8†	818.0†	806.0†	782

[1]Lennon *et al*. (2000), results from profile refinement.

[2]This work, our raw indexing of the data collected by Lennon *et al*. on HRPD in 1998, obtained using DICVOL06, figures of merit: 10 K, M(10) = 123.2, F(10) = 100.8 (0.0032, 31); 65 K, M(10) = 95.7, F(10) = 80.3 (0.0040, 31)

[3]Hudson *et al*. (2021); density measured using a quartz-crystal microbalance in an ultrahigh vacuum (~10^{-10} torr).

†Computed on the basis that C_3H_6 and C_3D_6 have identical molar volumes (see discussion in the main text, section 4.5, concerning volume isotope effects).

Table S2

Refined lattice parameters of propane-D8 as a function of temperature

T (K)	*a* (Å)	*b* (Å)	*c* (Å)	β (°)	V (Å^3)
Cooling series					
50	4.13351(3)	12.57259(3)	6.94421(3)	91.475(1)	360.764(2)
45	4.13012(3)	12.56825(5)	6.93954(4)	91.479(1)	360.100(3)
40	4.12701(3)	12.56446(5)	6.93553(4)	91.483(1)	359.512(3)
35	4.12450(3)	12.56061(5)	6.93212(5)	91.486(1)	359.006(3)
30	4.12240(3)	12.55778(5)	6.92945(5)	91.486(1)	358.604(3)
25	4.12106(3)	12.55562(5)	6.92761(5)	91.489(1)	358.330(3)
20	4.12023(3)	12.55417(5)	6.92650(5)	91.487(1)	358.160(3)
15	4.11975(3)	12.55334(5)	6.92604(5)	91.488(1)	358.070(3)
10.25	4.11965(2)	12.55313(3)	6.92571(3)	91.4880(5)	358.039(2)
Warming series					
12.5	4.11973(3)	12.55322(5)	6.92575(5)	91.488(1)	358.051(3)
17.5	4.12009(3)	12.55377(5)	6.92606(5)	91.486(1)	358.113(3)
22.5	4.12060(3)	12.55497(5)	6.92713(5)	91.488(1)	358.247(3)
27.5	4.12178(4)	12.55679(5)	6.92870(5)	91.488(1)	358.483(3)
32.5	4.12343(4)	12.55944(5)	6.93097(5)	91.485(1)	358.821(3)
37.5	4.12578(4)	12.56274(5)	6.93408(5)	91.485(1)	359.280(3)
42.5	4.12865(3)	12.56641(5)	6.93783(4)	91.481(1)	359.830(3)
47.5	4.13200(3)	12.57058(4)	6.94209(4)	91.479(1)	360.463(3)
55	4.13717(3)	12.57758(4)	6.94940(4)	91.472(1)	361.497(3)
60	4.14122(3)	12.58264(4)	6.95505(4)	91.468(1)	362.291(2)
65	4.14575(3)	12.58795(4)	6.96082(3)	91.464(1)	363.143(2)
70	4.15030(3)	12.59354(4)	6.96726(3)	91.458(1)	364.040(2)
75	4.15531(3)	12.59910(4)	6.97388(4)	91.452(1)	364.987(2)
80	4.16119(3)	12.60470(3)	6.98050(3)	91.444(1)	366.015(2)

Table S3

Refined lattice parameters of propene-D6 as a function of temperature

T (K)	*a* (Å)	*b* (Å)	*c* (Å)	V ($Å^3$)
Cooling series				
80	10.88588(5)	10.81653(4)	5.80422(2)	683.432(3)
70	10.85861(8)	10.79113(6)	5.80030(3)	679.660(5)
60	10.83420(8)	10.76778(7)	5.79815(3)	676.414(5)
50	10.81318(9)	10.74696(8)	5.79656(4)	673.611(6)
40	10.79546(8)	10.72859(7)	5.79546(3)	671.239(5)
30	10.78145(7)	10.71458(6)	5.79470(3)	669.397(5)
20	10.77213(8)	10.70550(7)	5.79442(3)	668.218(5)
10	10.76806(5)	10.70162(4)	5.79442(2)	667.724(3)
Warming series				
15	10.76910(7)	10.70288(7)	5.79454(3)	667.880(5)
25	10.77595(7)	10.70926(6)	5.79465(3)	668.716(5)
35	10.78732(8)	10.72076(7)	5.79549(3)	670.239(4)
45	10.80308(9)	10.73639(8)	5.79677(3)	672.345(6)
55	10.82208(8)	10.75589(7)	5.79843(3)	674.943(5)
65	10.84400(8)	10.77723(6)	5.80104(3)	677.957(5)
75	10.86995(7)	10.80212(5)	5.80375(2)	681.467(4)
85	10.90433(6)	10.83106(5)	5.80471(2)	685.569(4)

Table S4

Comparison of unit-cell dimensions and intramolecular bond lengths and angles in β-propane between various experimental and computational studies.

	This work, calculated athermal DFT	This work, observed, 10.25 K	Boese *et al*. (1999), 30 K
Unit-cell			
a (Å)	4.046010	4.11967(3)	4.1480(14)
b (Å)	12.290903	12.55244(4)	12.612(5)
c (Å)	6.787742	6.92536(4)	6.977(3)
β (°)	90.89715	91.4858(6)	91.28(3)
V (Å^3)	337.506988	358.003(3)	364.9(2)
Bond lengths (Å)			
C1–C2	1.5273	1.518(3)	1.531(1)
C2–C3	1.5273	1.534(2)	1.530(1)
C1–D11	1.0996	1.083(4)	0.965(15)
C1–D12	1.0998	1.104(3)	0.963(13)
C1–D13	1.0977	1.084(2)	0.984(15)
C2–D21	1.1027	1.091(4)	0.985(13)
C2–D22	1.1013	1.115(3)	0.969(15)
C3–D31	1.1001	1.084(4)	0.950(15)
C3–D32	1.0998	1.090(3)	0.974(14)
C3–D33	1.0980	1.091(3)	0.990(15)
Bond angles (°)			
C1–C2–C3	113.070	112.5(1)	112.14(7)
D11–C1–D12	107.246	106.7(3)	107.3(12)
D12–C1–D13	107.600	106.8(2)	106.6(11)
D11–C1–D13	107.461	108.3(3)	108.5(11)
D21–C2–D22	105.421	105.9(3)	107.3(11)
D31–C3–D32	107.200	107.2(3)	107.1(12)
D32–C3–D33	107.465	107.9(2)	107.1(12)

D31–C3–D33	107.696	108.5(3)	108.3(12)
Torsion angles (°)			
D13–C1–C2–C3	−176.465	−176.7(2)	−176.4(9)
D33–C3–C2–C1	177.529	178.0(2)	177.7(9)

Table S5

Comparison of experimentally- and computationally derived unit-cell dimensions and intramolecular bond lengths and angles in β-propene.

	This work, calculated athermal DFT	This work, observed, 10 K	This work, observed, 80 K
Unit-cell			
a (Å)	10.488594	10.76910(7)	10.88588(5)
b (Å)	10.561316	10.70288(7)	10.81653(4)
c (Å)	5.741236	5.79454(3)	5.80422(2)
V (Å^3)	635.975955	667.880(5)	683.432(3)
Bond lengths (Å)			
C1–C2	1.3369	1.338(4)	1.319(5)
C2–C3	1.4929	1.495(4)	1.479(5)
C1–D11	1.0894	1.085(4)	1.075(6)
C1–D12	1.0919	1.083(4)	1.062(6)
C2–D21	1.0952	1.079(4)	1.074(6)
C3–D31	1.1016	1.083(4)	1.082(6)
C3–D32	1.1018	1.090(5)	1.083(6)
C3–D33	1.0977	1.111(4)	1.086(6)
C4–C5	1.3368	1.329(4)	1.332(5)
C5–C6	1.4937	1.495(3)	1.496(5)
C4–D41	1.0908	1.093(4)	1.087(6)
C4–D42	1.0912	1.103(4)	1.080(6)
C5–D51	1.0945	1.088(4)	1.064(5)
C6–D61	1.1009	1.090(4)	1.090(6)
C6–D62	1.1028	1.099(4)	1.068(6)
C6–D63	1.0979	1.090(4)	1.072(6)
Bond angles (°)			
C1–C2–C3	125.190	124.7(2)	125.0(3)

D11–C1–D12	116.654	116.2(3)	115.2(5)
D21–C2–C1	118.987	118.6(3)	118.9(4)
D31–C3–D32	106.247	108.1(3)	106.2(5)
D32–C3–D33	108.615	107.7(3)	106.6(5)
D31–C3–D33	108.347	108.1(3)	107.5(5)
C4–C5–C6	125.209	125.5(2)	125.1(3)
D41–C4–D42	117.109	117.2(3)	117.7(5)
D51–C5–C4	119.103	117.4(3)	118.7(4)
D61–C6–D62	106.167	107.0(3)	107.2(4)
D62–C6–D63	108.693	107.9(3)	111.1(5)
D61–C6–D63	108.211	109.3(3)	106.8(5)
Torsion angles (°)			
D33–C3–C2–C1	1.465	0.0(4)	1.2(6)
D63–C6–C5–C4	−2.720	−1.3(4)	−3.7(6)

Table S6

Parameters obtained from fitting Debye-type models to the lattice parameters of propane-D8. The last four rows of the table report values derived from the parameters in the preceding rows.

	Cell volume	*a*-axis	*a*sinβ
θ_D (K)	166(3)	163(6)	163(6)
V_0 ($cm^3\ mol^{-1}$)	53.901(1)	10.5251(6)	10.5145(6)
Q ($J\ cm^{-3}$)	4.5(2)x10^5	3.6(2)x10^5	3.6(2)x10^5
b	3.1(8)	5(1)	5(1)
Debye cut-off (cm^{-1})	116(2)	114(4)	114(4)
V_0 (Å^3)	358.019(4)	69.909(2)	69.839(2)
K_0/γ (GPa)	8.3(3)	34(2)	34(2)
K'_0	7(2)	10(2)	10(2)

	b-axis	*c*-axis
θ_D (K)	150(1)	184(1)
V_0 ($cm^3\ mol^{-1}$)	297.805(4)	50.012(1)
Q ($J\ cm^{-3}$)	8.24(5)x10^5	3.55(3)x10^5
b	0*	0*
Debye cut-off (cm^{-1})	104(1)	128(9)
V_0 (Å^3)	1978.07(1)	332.188(4)
K_0/γ (GPa)	2.77(2)	7.10(5)
K'_0	1*	1*

*Parameter value fixed

Table S7

Parameters obtained from fitting Debye-type models to the lattice parameters of propene-D6. The last four rows of the table report values derived from the parameters in the preceding rows.

	Cell volume	*a*-axis	*b*-axis
θ_D (K)	111(1)	102(8)	114(8)
V_0 (cm^3 mol^{-1})	50.2622(8)	93.98(1)	92.26(1)
Q (J cm^{-3})	5.66(7)x10^5	4.4(4)x10^5	3.7(3)x10^5
b	12.4(4)	10(2)	6(2)
Debye cut-off (cm^{-1})	77.1(9)	71(6)	79(5)
V_0 (Å^3)	667.698(3)	1248.44(5)	1225.56(4)
K_0/γ (GPa)	11.3(1)	4.7(4)	4.1(3)
K'_0	22.9(8)	22(4)	13(3)

	c-axis (warming)	*c*-axis (cooling)	
θ_D (K)	237(8)	237*	
V_0 (cm^3 mol^{-1})	14.6455(5)	14.6453(6)	
Q (J cm^{-3})	9.3(4)x10^5	1.63(9)x10^6	
b	0*	116(17)	
Debye cut-off (cm^{-1})	164(6)	164*	
V_0 (Å^3)	194.555(2)	194.553(2)	
K_0/γ (GPa)	63(3)	111(6)	
K'_0	1*	232(35)	

*Parameter value fixed

Table S8

Correlation table for propane phase II, C_3H_8, (space group $P2_1/n$, no. 14).

Free molecule (C_{2v}) Rep.[2]		Site[1] (C_1)	Crystal Factor group (C_{2h}, $Z = 4$) Rep.
External	Internal		
$A_1 + B_1 + B_2$ (trans)		3 A	3 A_u + 3 A_g + 3 B_u + 3 B_u
$A_2 + B_1 + B_2$ (lib)		3 A	3 A_u + 3 A_g + 3 B_u + 3 B_u
	9 A_1	9 A	9 A_u + 9 A_g + 9 B_u + 9 B_u
	5 A_2	5 A	5 A_u + 5 A_g + 5 B_u + 5 B_u
	7 B_1	7 A	7 A_u + 7 A_g + 7 B_u + 7 B_u
	6 B_2	6 A	6 A_u + 6 A_g + 6 B_u + 6 B_u

[1] Symmetry of the site occupied by the molecule in the crystal.

[2] Rep. = irreducible representation of the point group, trans = translation, lib = libration.

Table S9

Correlation table for propene phase II, C_3H_6, (space group $P2_12_12_1$, no. 19).

Free molecule (C_s) Rep. [2]		Site [1] (C_1)	Crystal Factor group (D_2, $Z = 8$) Rep.
External	Internal		
$2\,A' + A''$ (trans)		$3\,A$	$6\,A + 6\,B_1 + 6\,B_2 + 6\,B_3$
$A' + 2\,A''$ (lib)		$3\,A$	$6\,A + 6\,B_1 + 6\,B_2 + 6\,B_3$
	$14\,A'$	$14\,A$	$28\,A + 28\,B_1 + 28\,B_2 + 28\,B_3$
	$7\,A''$	$7\,A$	$14\,A + 14\,B_1 + 14\,B_2 + 14\,B_3$

[1] Symmetry of the site occupied by the molecule in the crystal.

[2] Rep. = irreducible representation of the point group, trans = translation, lib = libration.

Table S10

Correlation table for propene phase I, C_3H_6, (space group $I\bar{4}$, no. 82).

Free molecule (C_s) Rep. [2]		Site [1] (C_1)	Crystal Factor group (S_4, $Z = 4$) Rep.
External	Internal		
$2\,A' + A''$ (trans)		$3\,A$	$3\,A + 3\,B + 3\,E$
$A' + 2\,A''$ (lib)		$3\,A$	$3\,A + 3\,B + 3\,E$
	$14\,A'$	$14\,A$	$14\,A + 14\,B + 14\,E$
	$7\,A''$	$7\,A$	$7\,A + 7\,B + 7\,E$

[1] Symmetry of the site occupied by the molecule in the crystal.

[2] Rep. = irreducible representation of the point group, trans = translation, lib = libration.

Figure S1
Neutron powder diffraction data collected at 10 K from propene-D6 on HRPD in October 1998. Tick marks are from the indexing of Lennon *et al*. ($P2$, $P2_1$, Pm in red at the bottom, Pc in black, middle) and our own analysis ($I4$, blue uppermost tick marks).

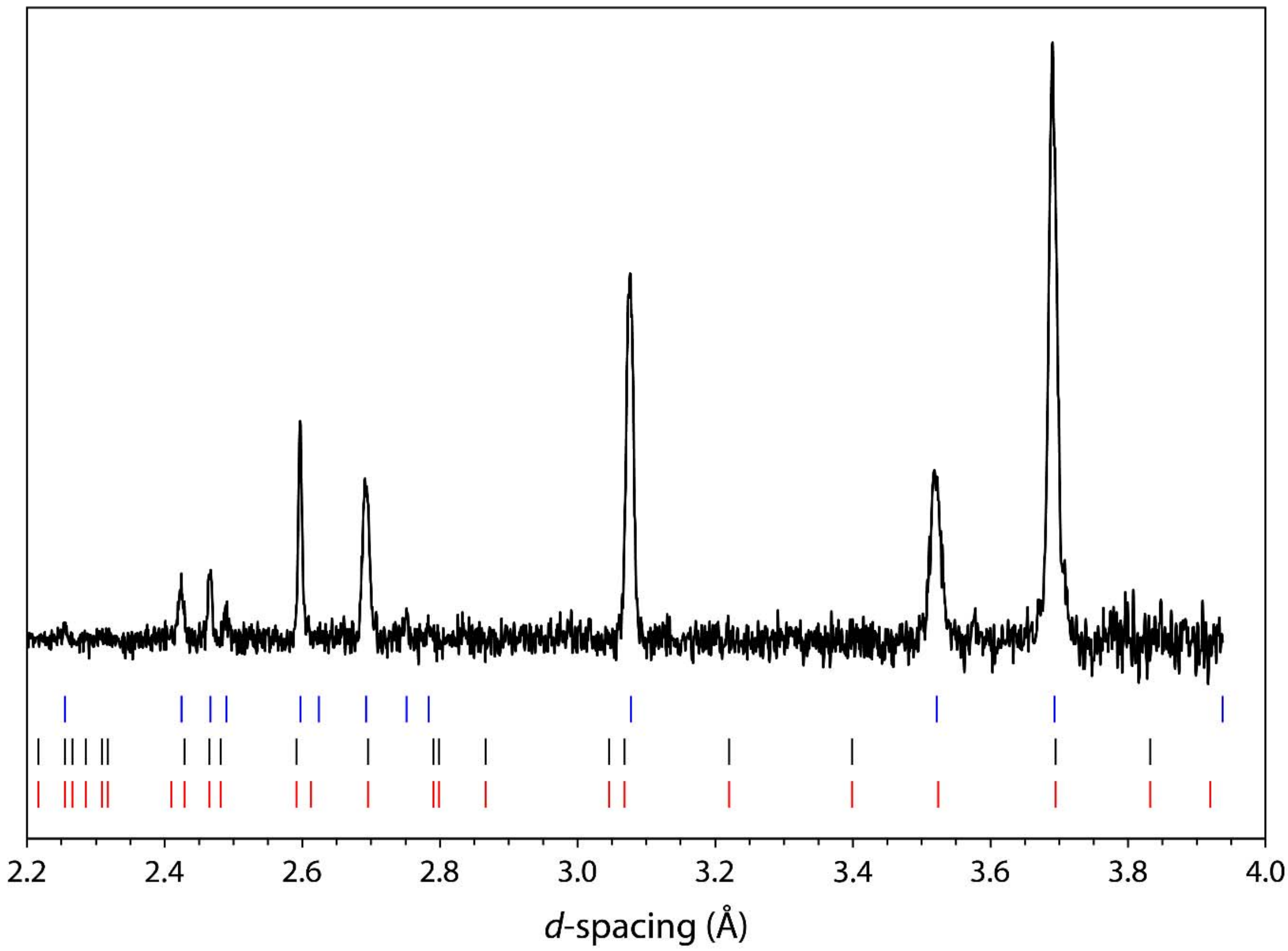

Figure S2

Temperature dependence of the lattice parameters of propane-D8, with filled symbols denoting measurements made on cooling, open symbols indicating measurements made on heating, and solid lines reporting the Debye model fits described in the main text (see Table S6 for fit parameters).

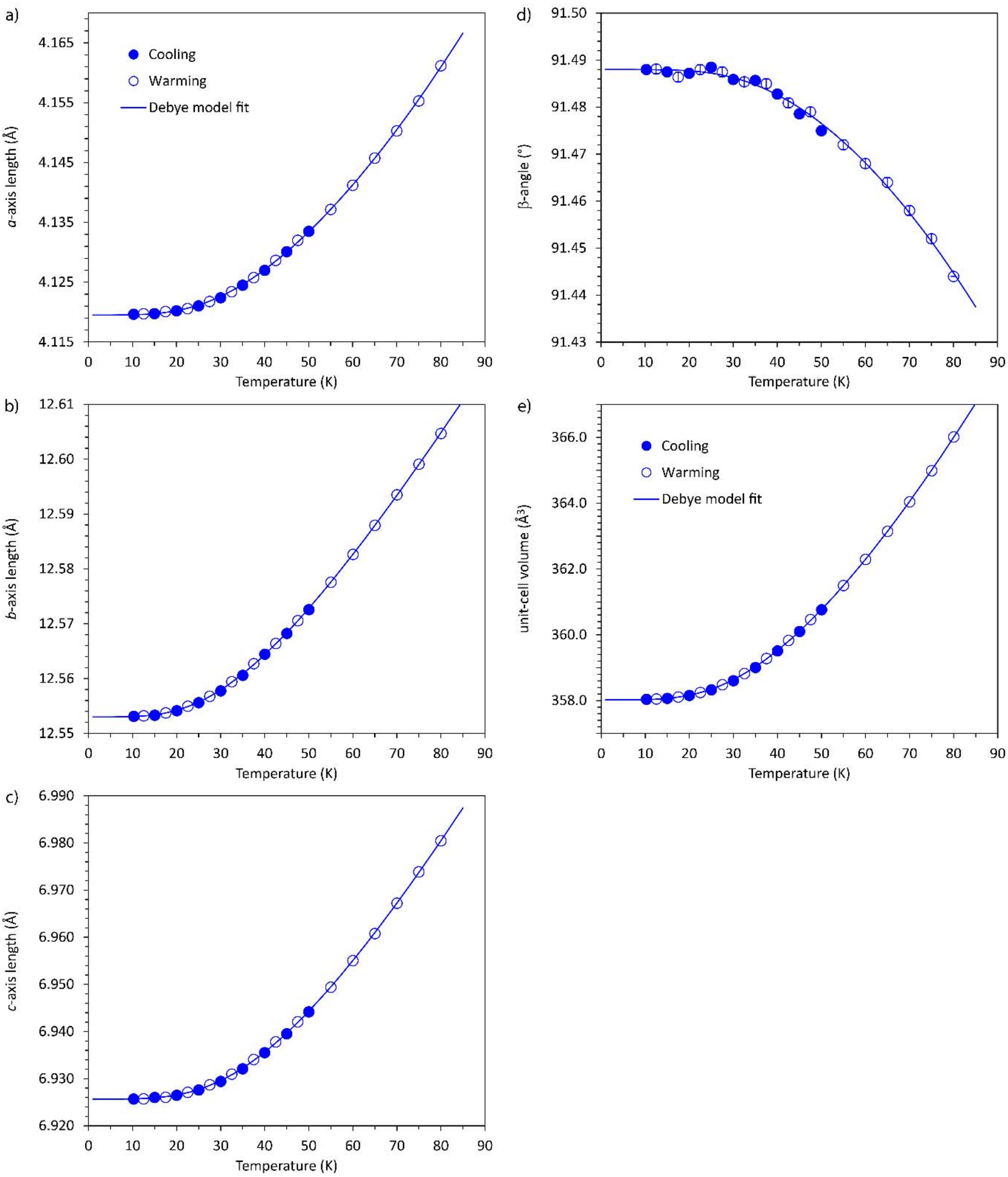

Figure S3

Rietveld plot of the 10 K neutron powder diffraction data collected from propane-D8, with the data shown as filled red circles, the fitted model as a solid green line, the difference between the model and the observations as a purple line at the bottom, and the positions of the Bragg peaks denoted with vertical black tick marks beneath the diffraction pattern.

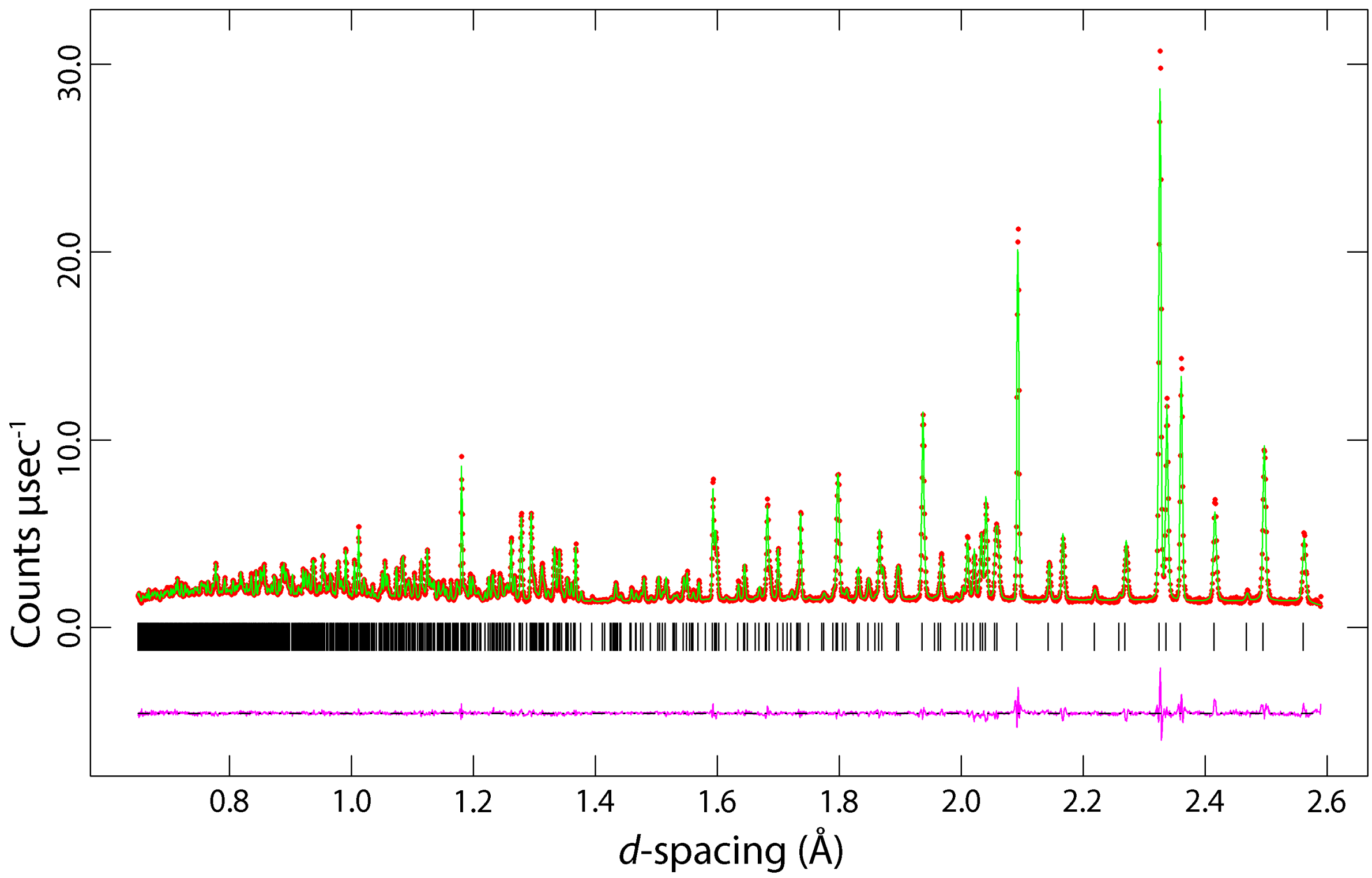

Figure S4

Temperature dependence of the lattice parameters of propene-D6, with filled symbols denoting measurements made on cooling, open symbols indicating measurements made on heating, and solid lines reporting the Debye model fits described in the main text (see Table S7 for fit parameters).

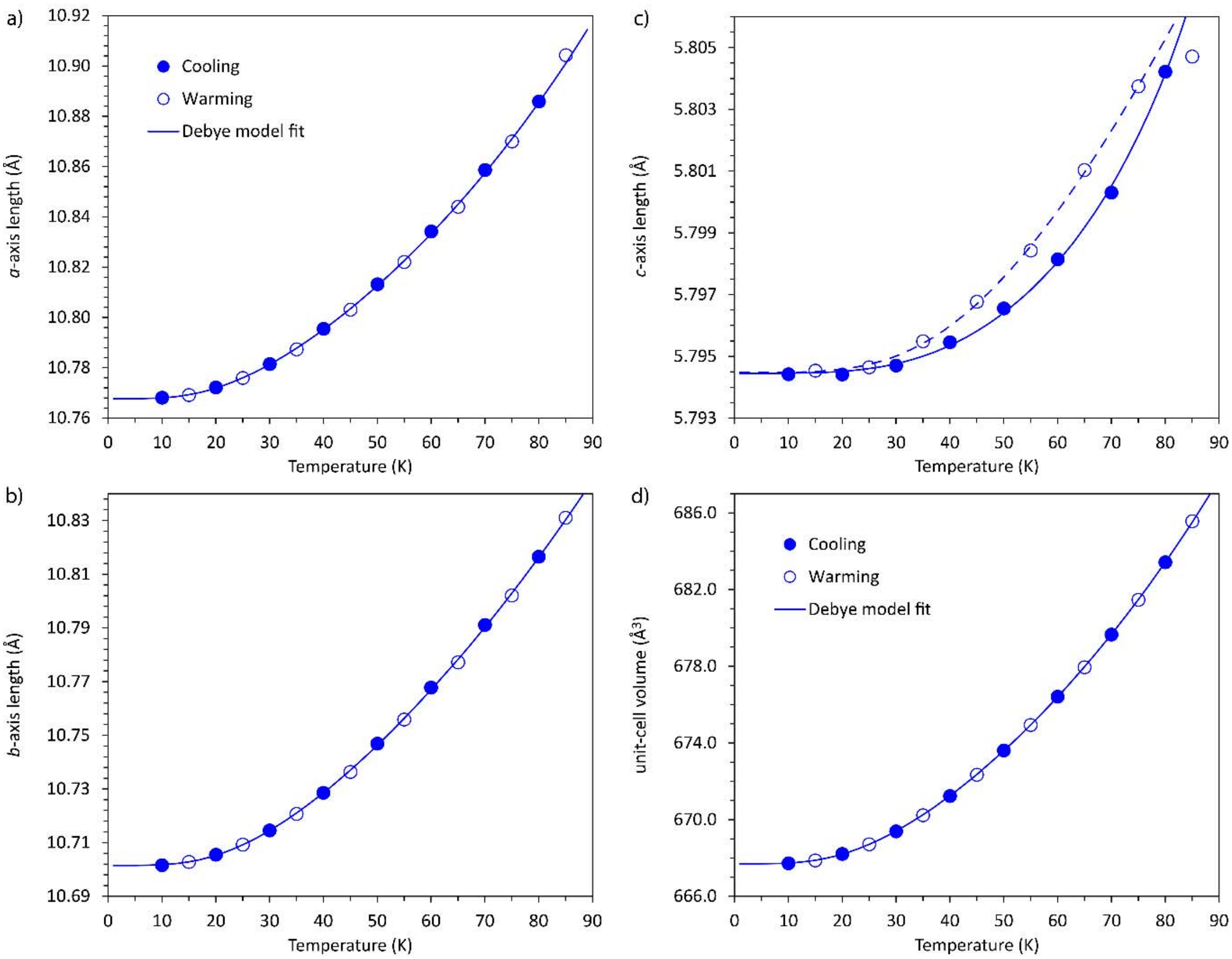

Figure S5

Rietveld plot of the 10 K neutron powder diffraction data collected from propene-D6.

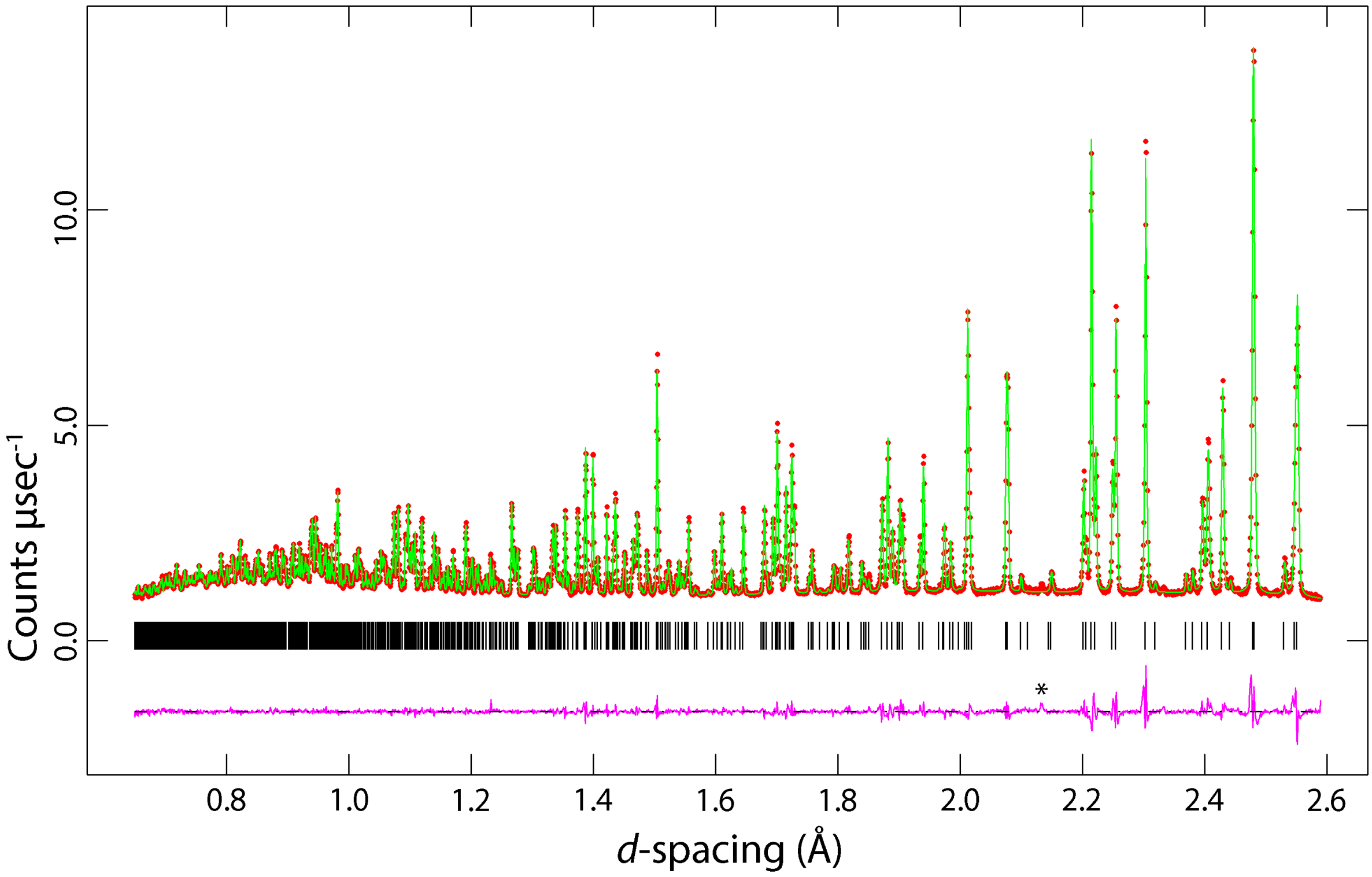

Figure S6

Rietveld plot of the 80 K neutron powder diffraction data collected from propene-D6.

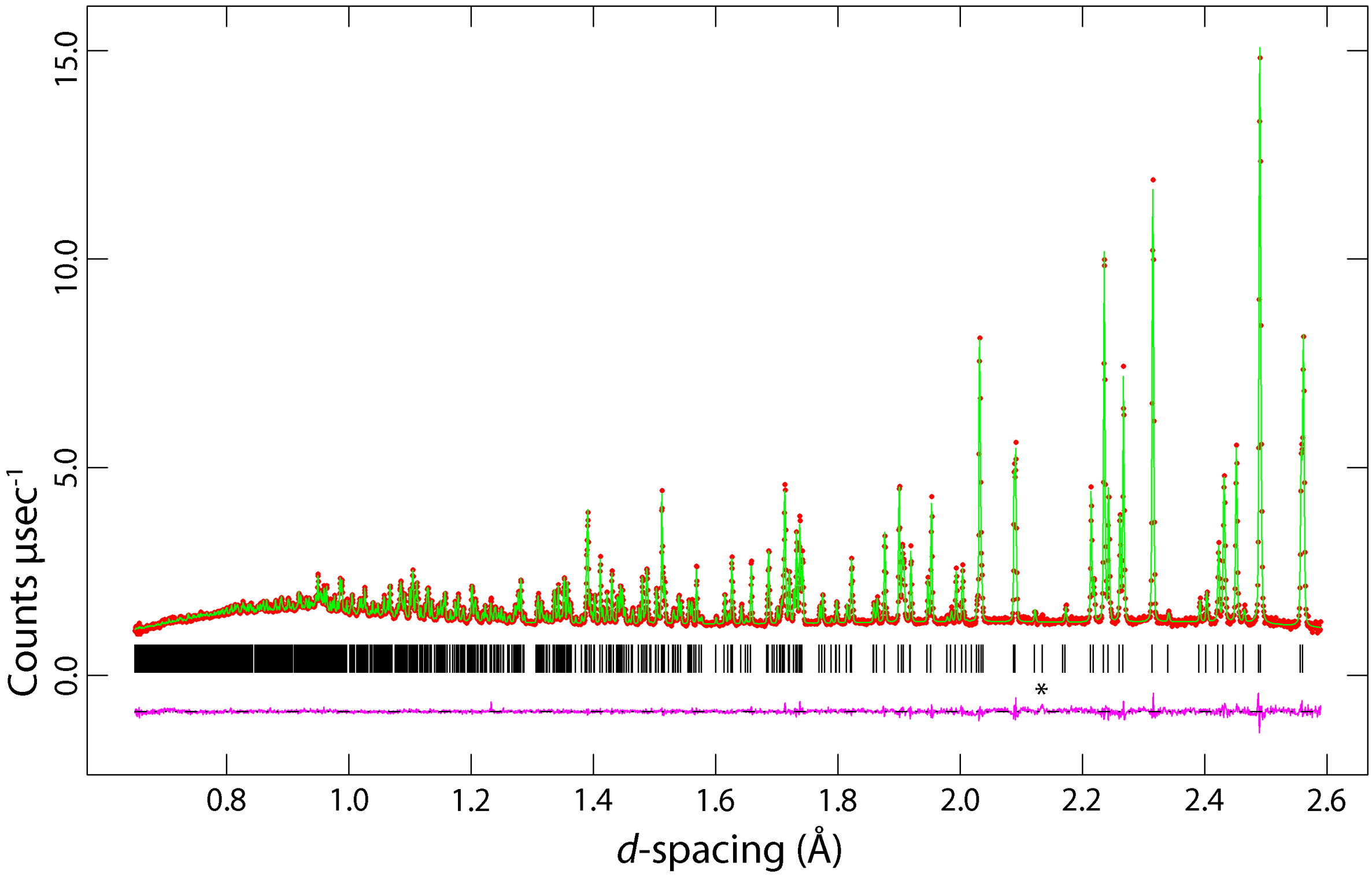

Figure S7

Fingerprint plot derived from the Hirschfeld surface of the propane molecule, computed using the 10 K structure obtained in this work. The plot reports distances between the Hirschfeld surface and nuclei inside the surface (d*i*) and outside the surface (d*e*), colour coded according to the proportion of the surface area involved in any particular pair-wise interaction. In this instance, 100 % of the contacts are H···H contacts.

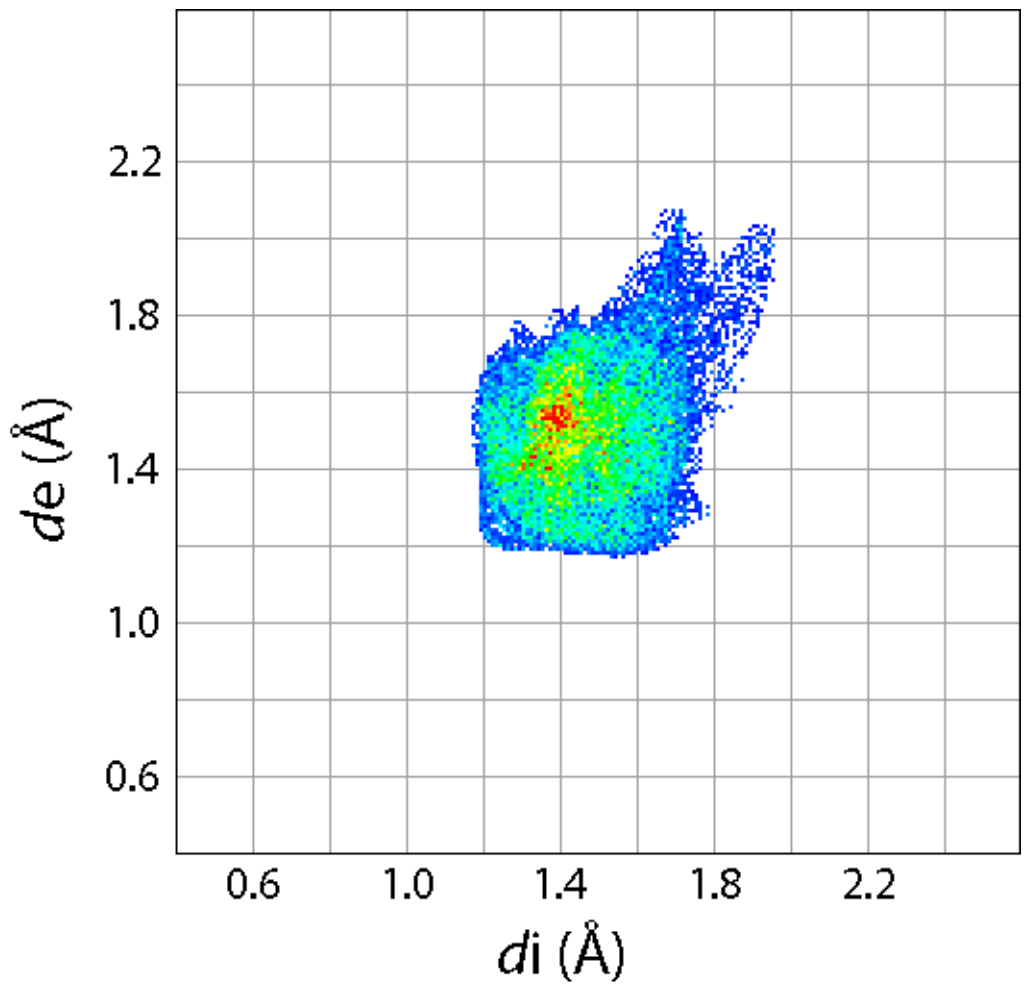

Figure S8

Fingerprint plots derived from the Hirschfeld surface of the propene molecule, computed using the 10 K structure obtained in this work. In contrast to propane, the contacts can be distinguished into two categories: (a) ~ 88 % of the contacts are H···H contacts; (b) the remaining 12 % are H···C contacts, and more specifically the C–H···π interactions described in the main text.

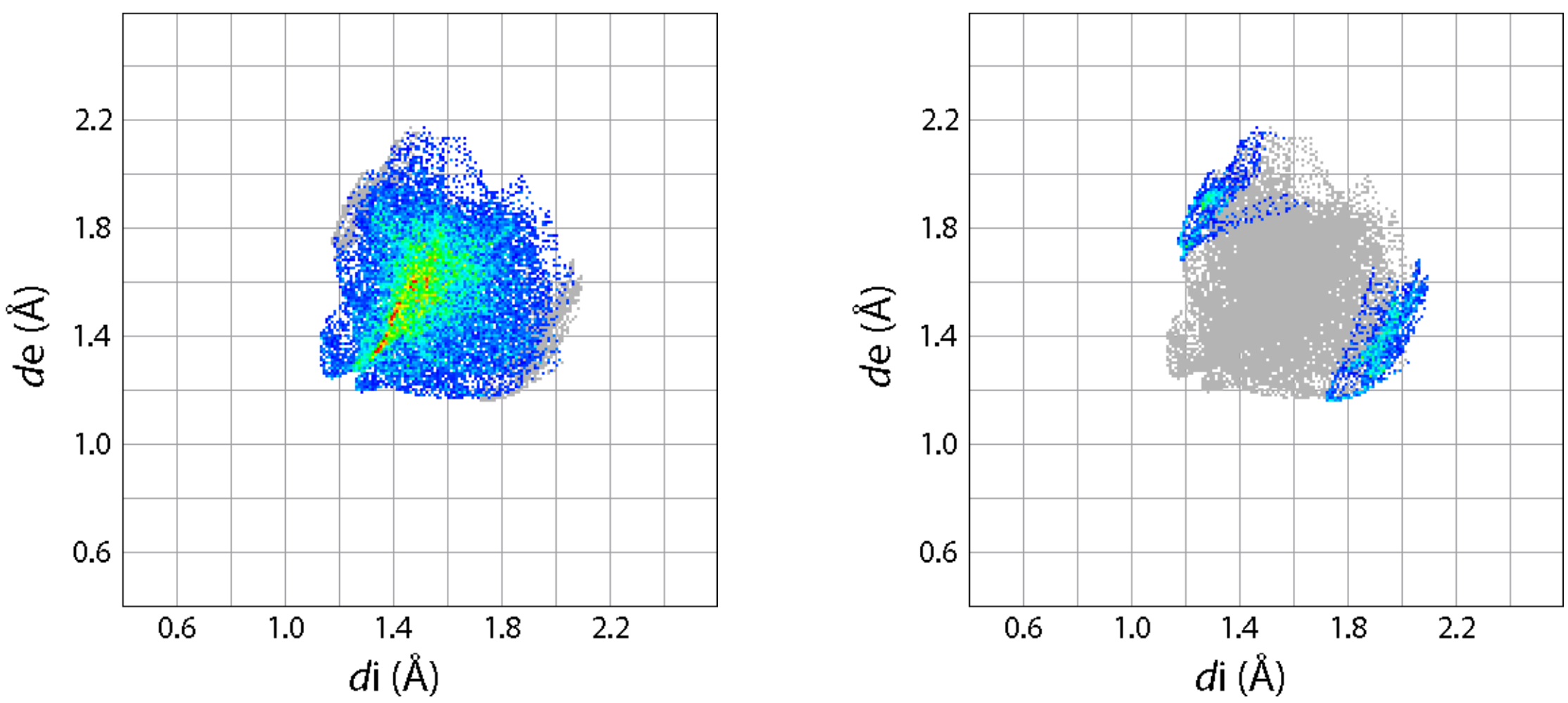

Figure S9

Plot of total energy against density for the 638 candidate structures produced by the Polymorph module in Materials Studio following searches in the eight possible body-centred tetragonal space groups consistent with the diffraction data of Lennon *et al*. (2000). The solution with the lowest energy (and highest density) is compared with the experimental structure in Figure S10 (following geometry optimisation in CASTEP).

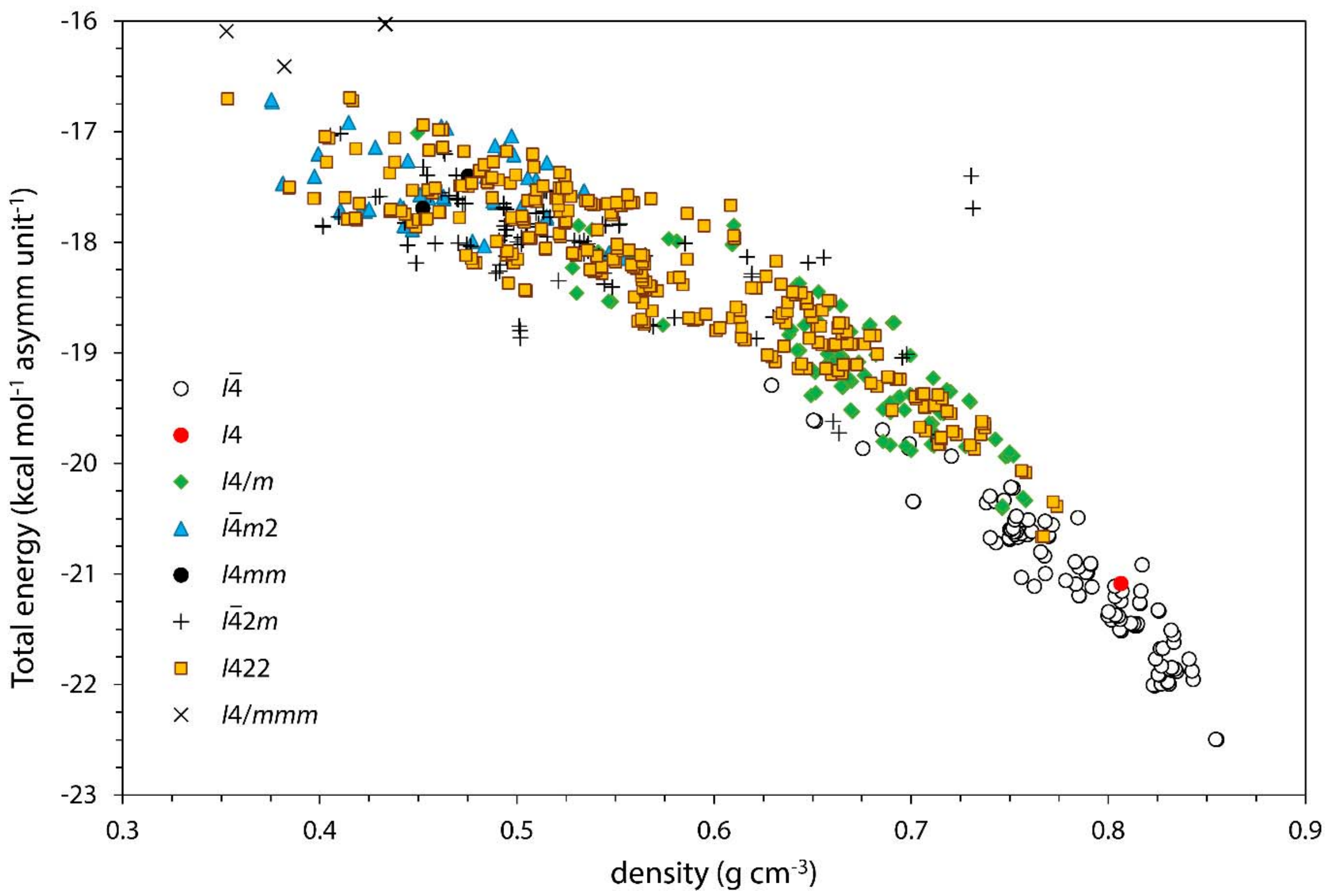

Figure S10

Overlay of the experimental $P2_12_12_1$ structure of propene (red) and the predicted $I\bar{4}$ structure (blue), focusing on the structural motifs highlighted in Figure 3 of the main text. The single most significant difference is a rotation of the P2 molecule around its long axis.

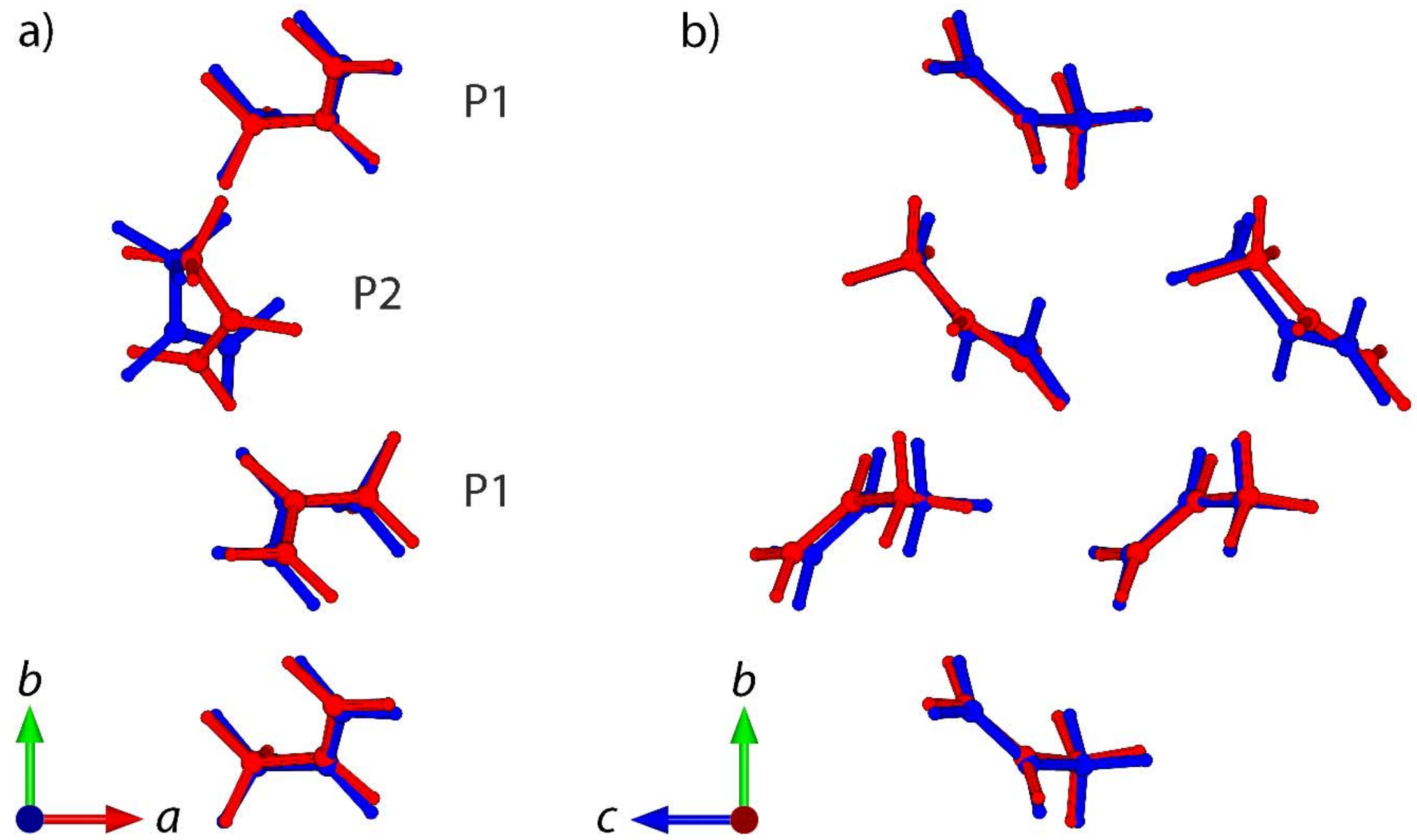

Figure S11

The same view as illustrated in Figure S10 following a rotation of the P2 molecules by 127 degrees about an axis between the centres of C6 and D41.

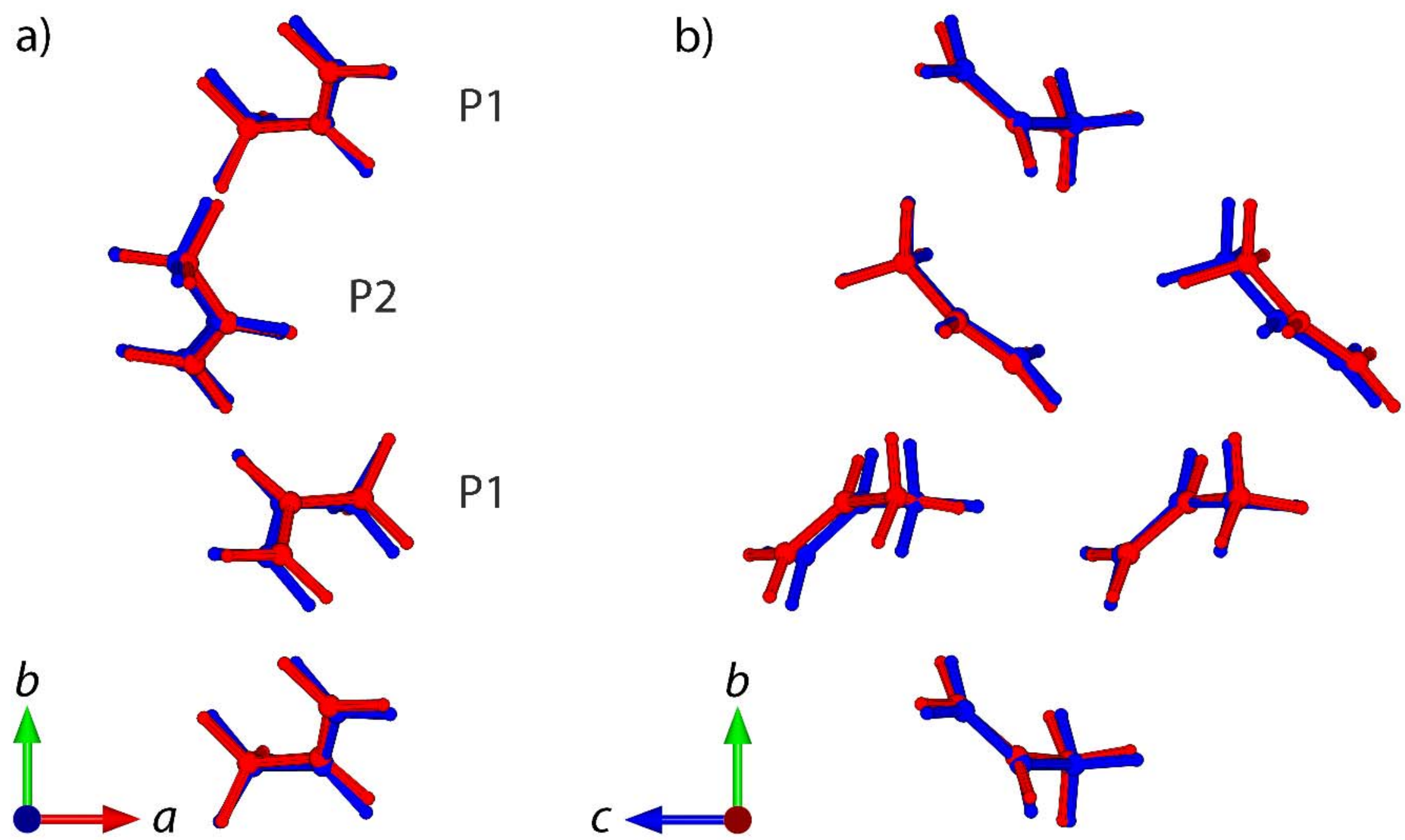

Figure S12

Comparison of the propene-D6 diffraction pattern observed on HRPD in 1998 (black, bottom) with the simulated diffraction pattern of the $I\bar{4}$ structure (red, top).

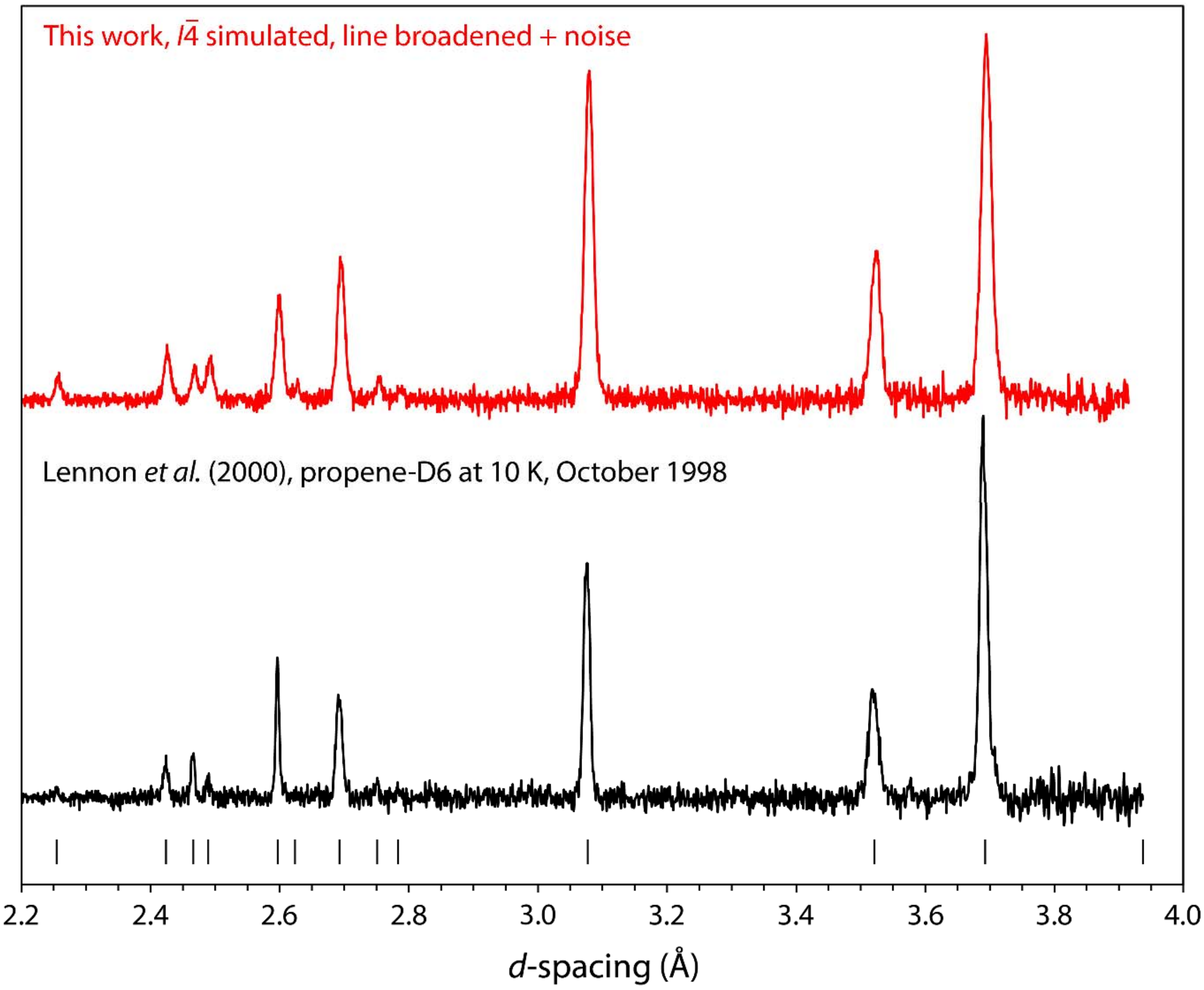

Figure S13

(a) Simulated neutron powder diffraction patterns of propene in the $I\bar{4}$ structure with different degrees of H/D substitution, scaled to display the strongest peak (211 at 3.694 Å *d*-spacing) with the same intensity), showing the large variations in Bragg peak relative intensities resulting from even modest degrees of exchange. Compare with Figure S12 above. (b) Expanded view of the simulated 310 peak showing the systematic decrease in intensity with increasing protium content.

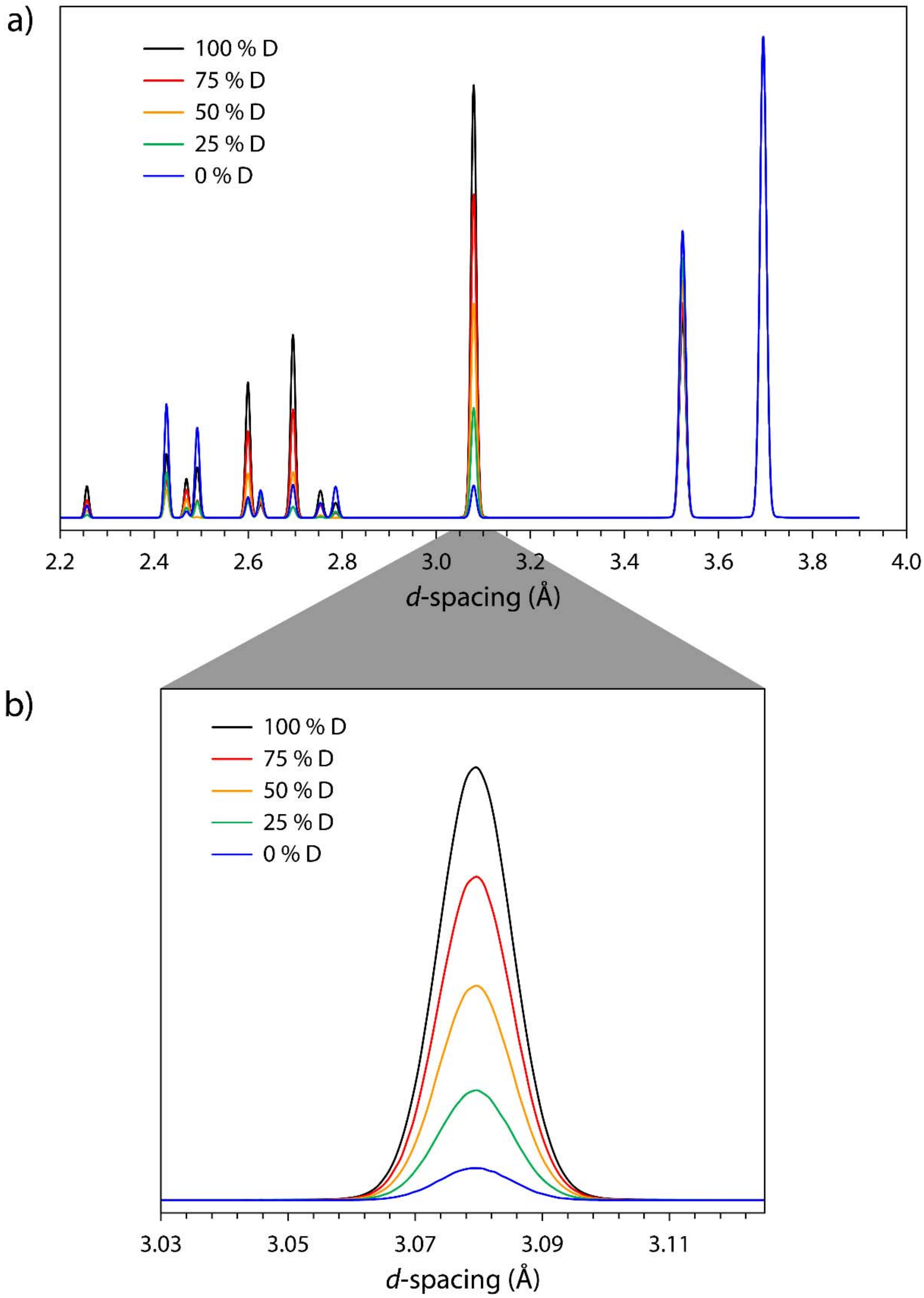

Figure S14

Simulated neutron powder diffraction patterns of propene in the $I\bar{4}$ structure with complete H/D substitution on different parts of the molecule, scaled to display the strongest peak (211 at 3.694 Å *d*-spacing) with the same intensity).

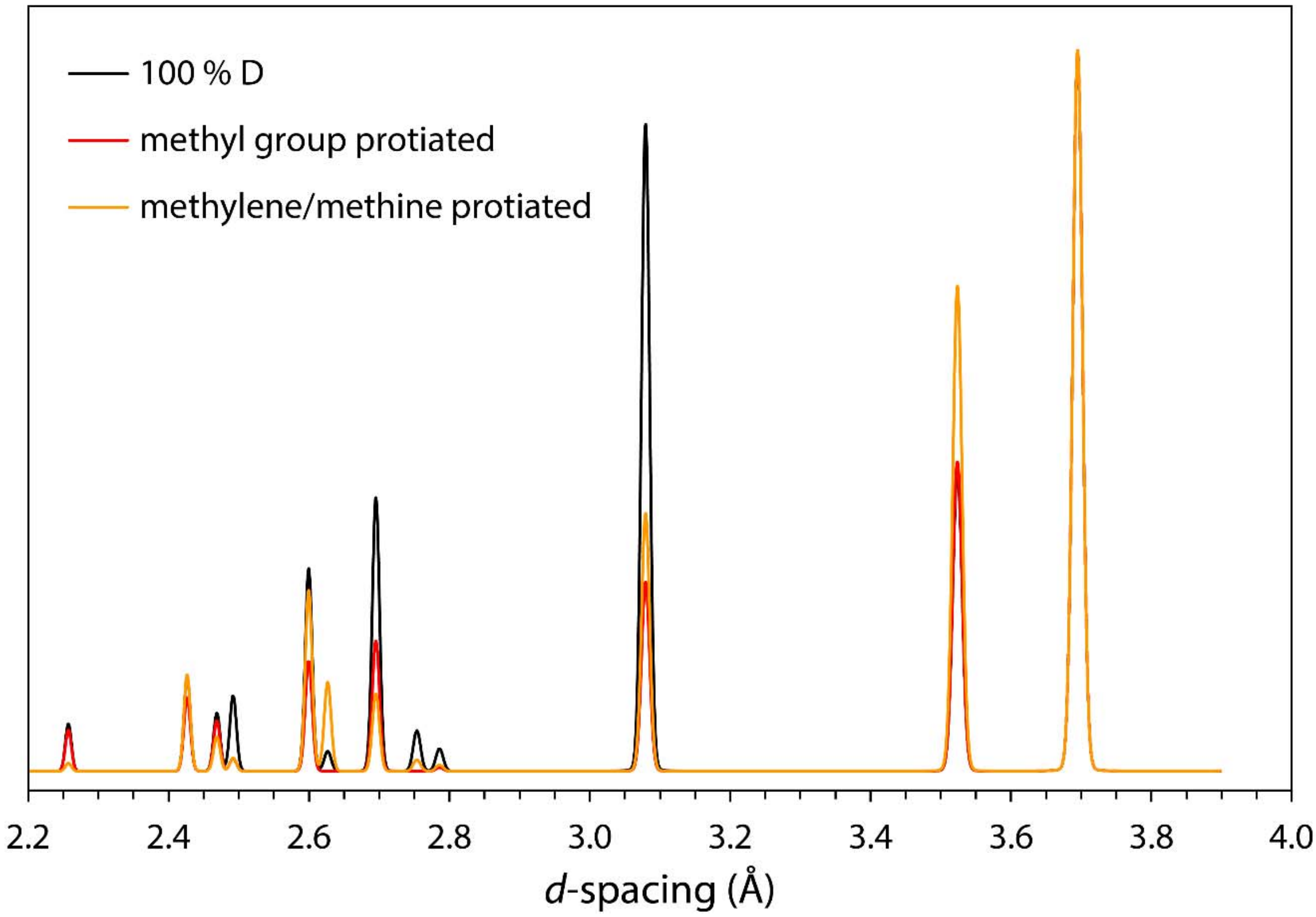

Figure S15

Comparison of the INS spectra measured on TOSCA with those calculated from first principles with CASTEP and AbINS for propane-H8 (a) and propane-D8 (b).

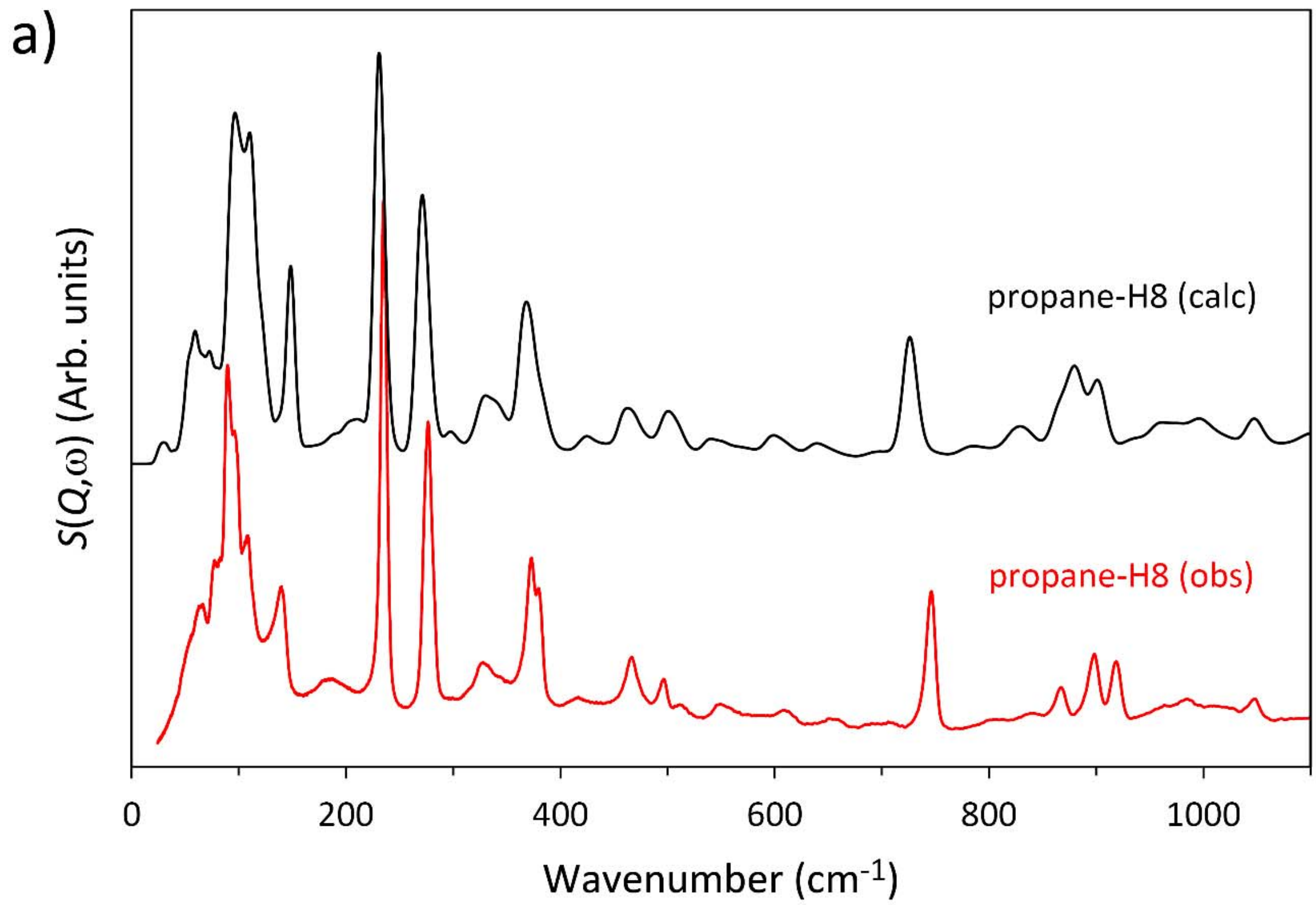

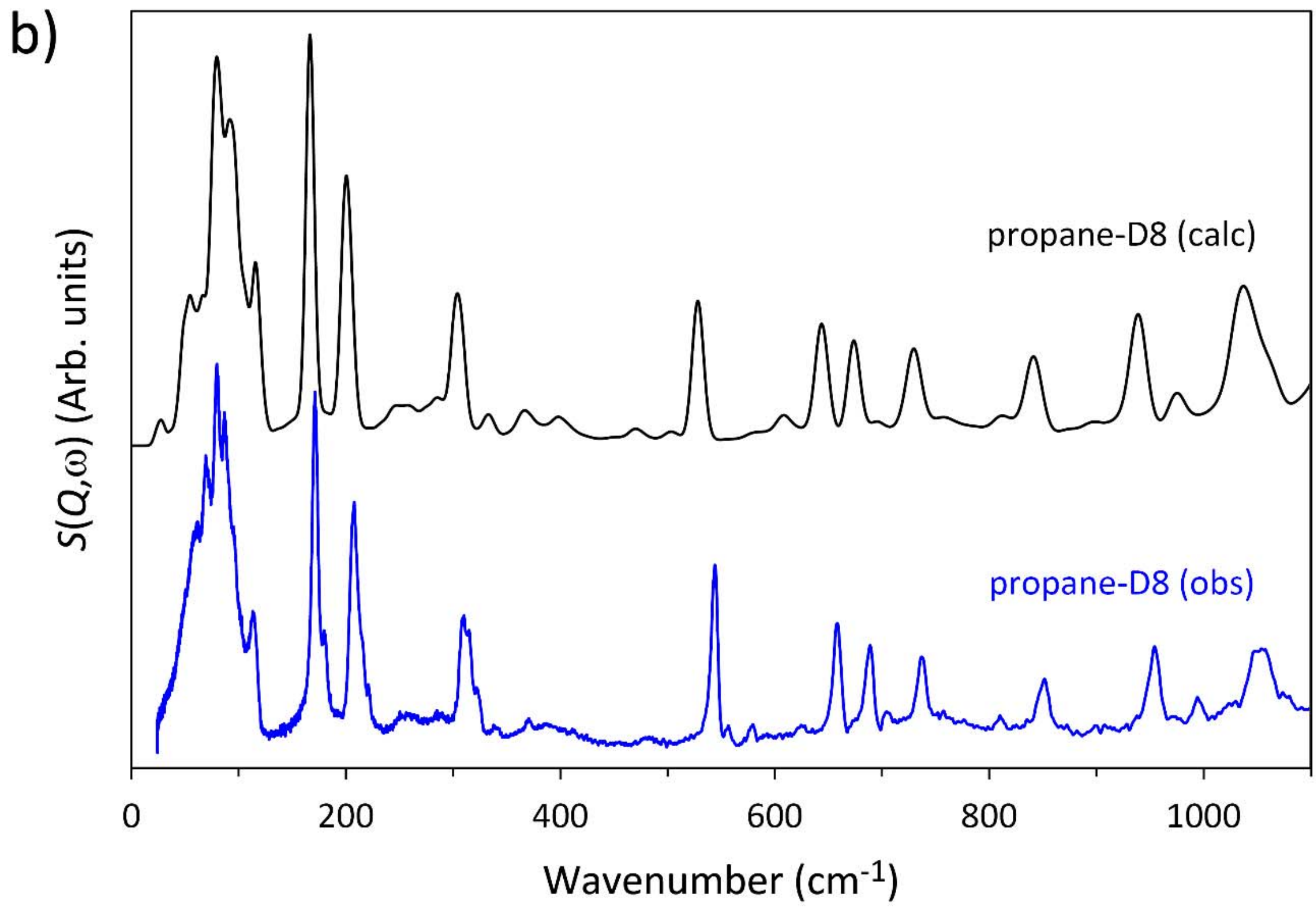

Figure S16

Calculated phonon dispersion curves of propane-H8. Note the breaks and changes in scale of the vertical axis. The path through reciprocal space indicated on the horizontal axis is depicted in Suppl. Fig. S18.

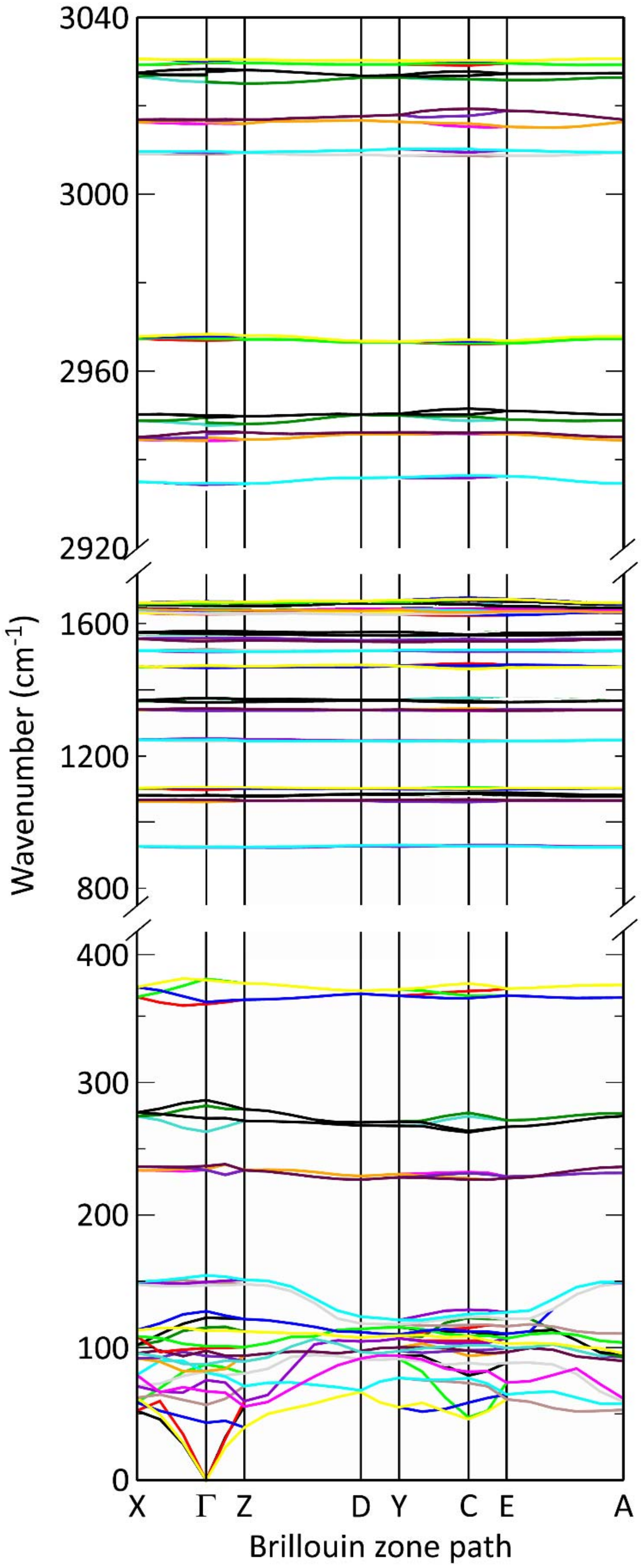

Figure S17

Calculated phonon dispersion curves of propane-D8. Note the breaks and changes in scale of the vertical axis. The path through reciprocal space indicated on the horizontal axis is depicted in Suppl. Fig. S18.

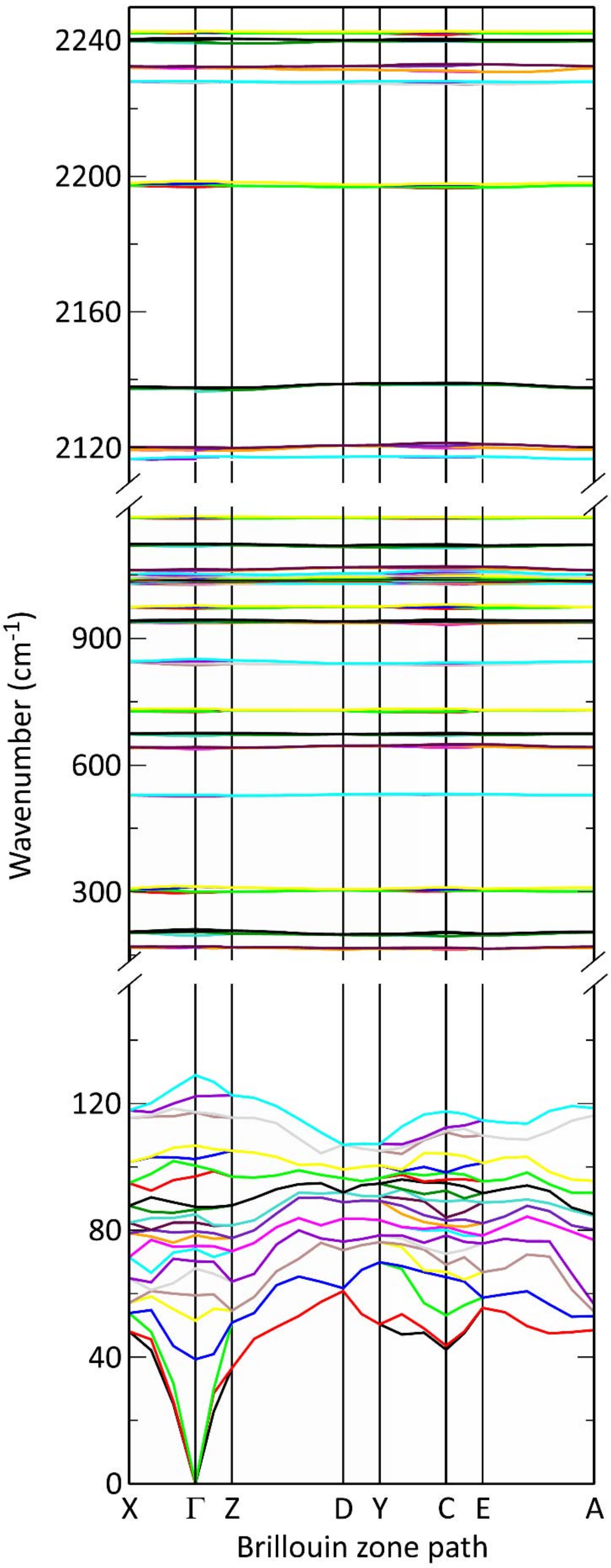

Figure S18

The first Brillouin zone of monoclinic β-propane with the high symmetry points labelled and the path between each point followed in the phonon dispersion curves (Figures S8 and S9) marked in red. The labelled points are at Γ (0,0,0), X (0,0,½), Z (0,½,0), D (½,½,0), Y (½,0,0), C (½,0,½), E (½,½,½), and A (0,½,½).

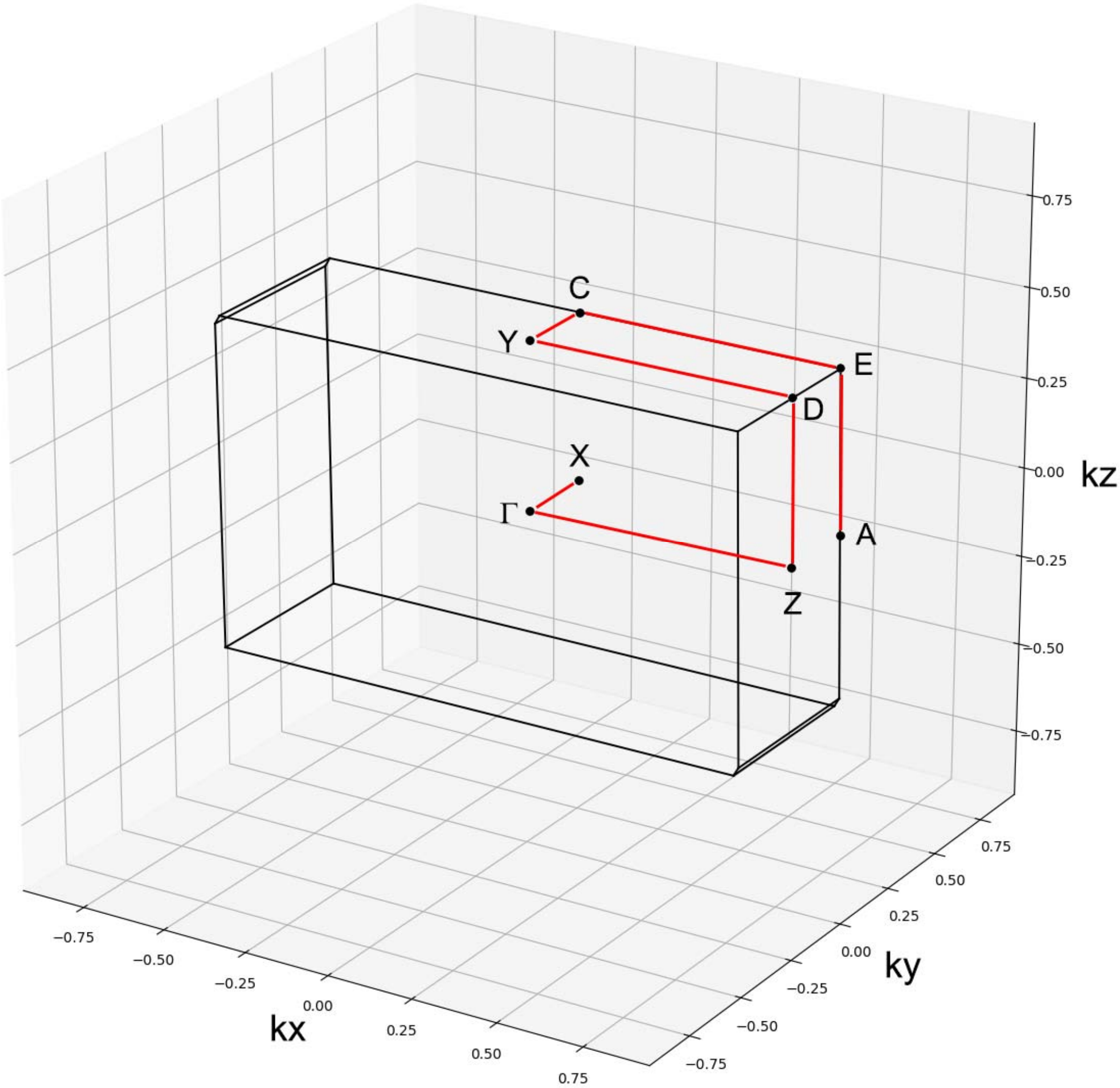

Figure S19

Extended range comparison of observed, (a) and calculated in the $P2_12_12_1$ phase (b) and in the $I\bar{4}$ phase (c) INS spectra of propene-H6.

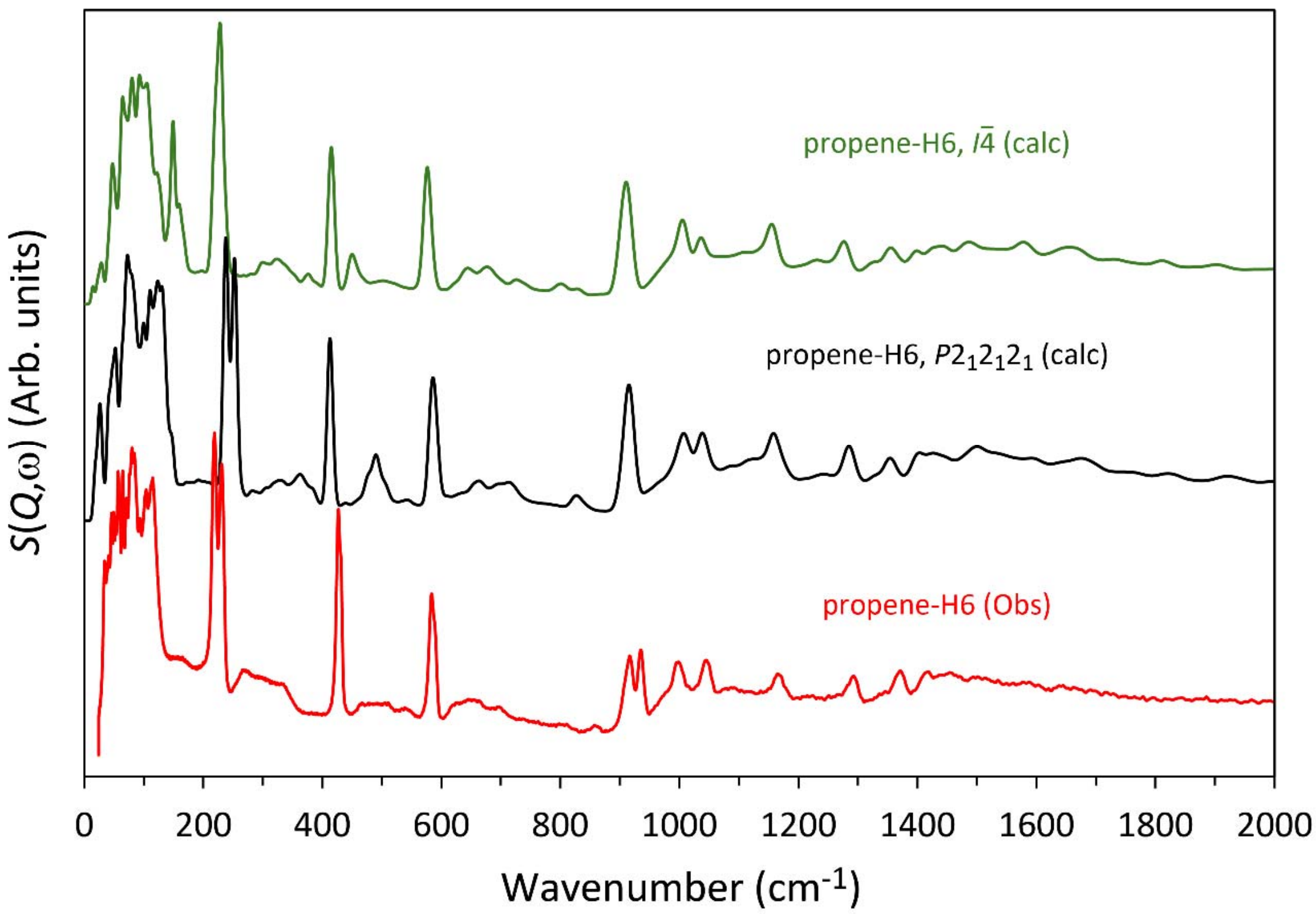

Figure S20

Calculated phonon dispersion curves of propene-H6 in the $P2_12_12_1$ phase (a) and in the $I\bar{4}$ phase (b). Note the breaks and changes in scale of the vertical axis. The respective paths through reciprocal space indicated on the horizontal axes are depicted in Suppl. Fig. S21.

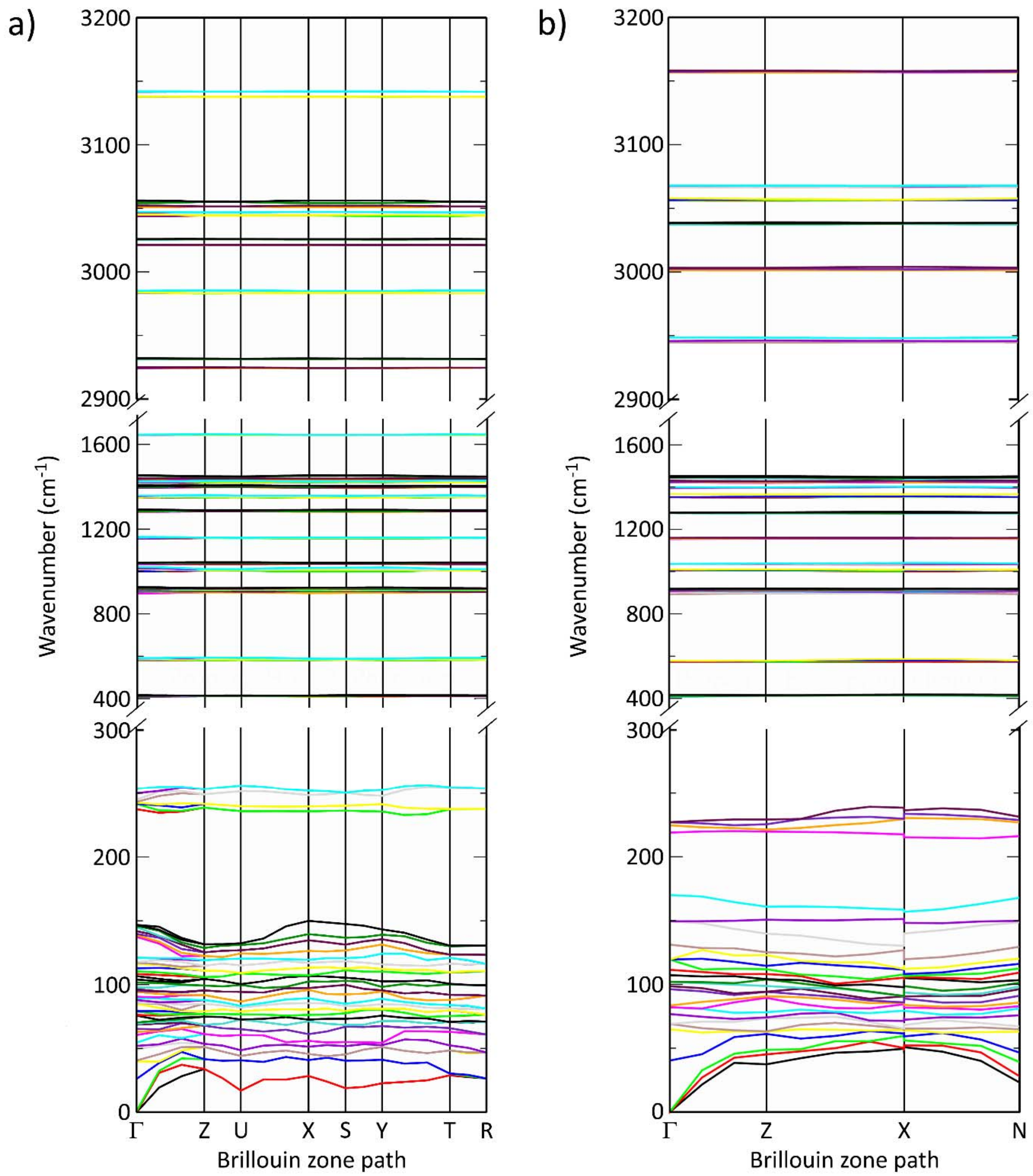

Figure S21

The first Brillouin zones of primitive orthorhombic propene (a) and body-centred tetragonal propene (b), with the high symmetry points labelled and the paths between each point followed in the phonon dispersion curves marked in red. The high symmetry points in the orthorhombic BZ are Γ (0,0,0), Z (0,0,½), U (½,0,½), X (½,0,0), S (½,½,0), Y (0,½,0), T (0,½,½), and R (½,½,½). The high symmetry points in the body-centred tetragonal BZ are Γ (0,0,0), X (½,0,0), Z (½,½,−½), and N (0,½,0).

a)

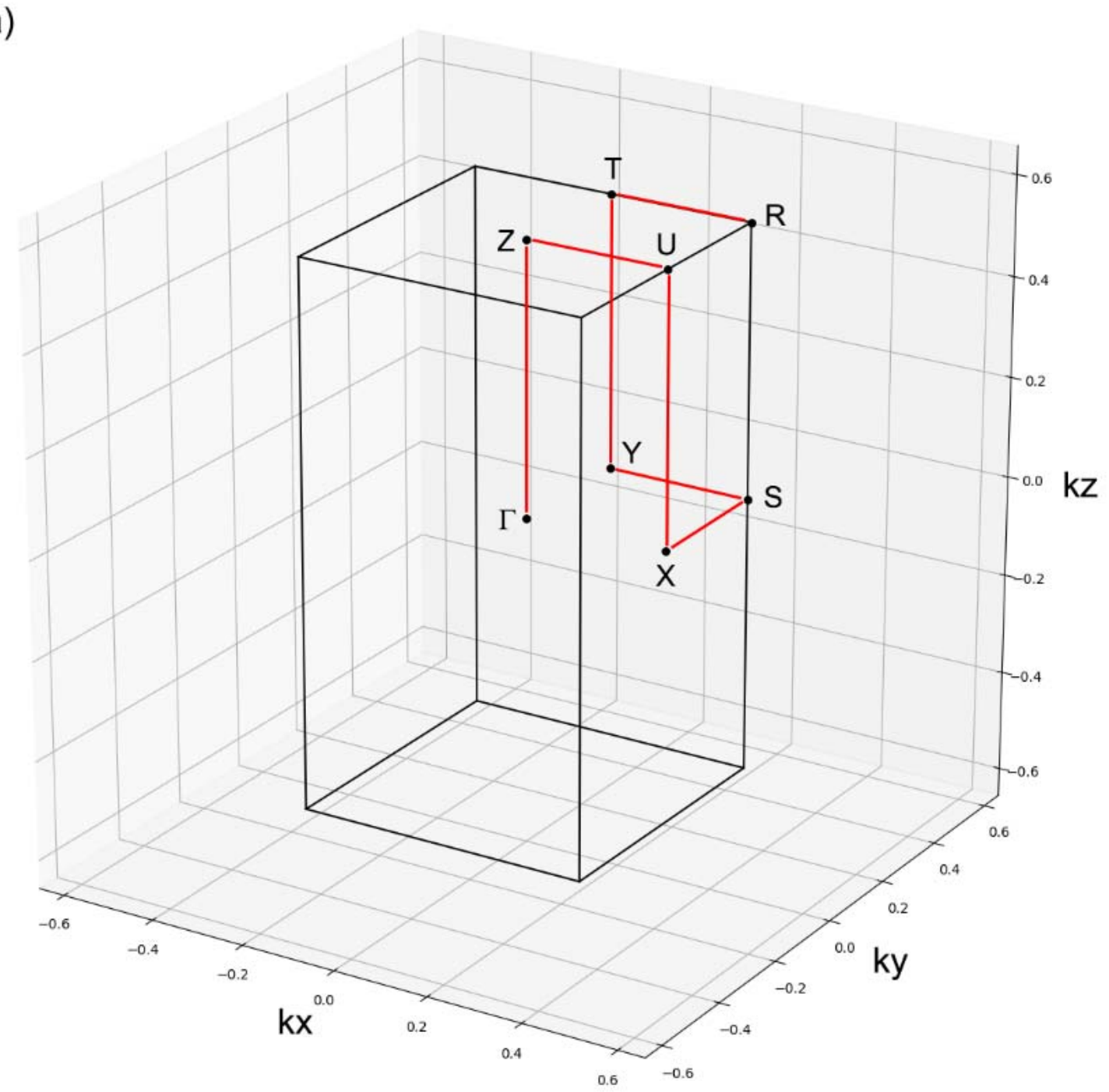

b)

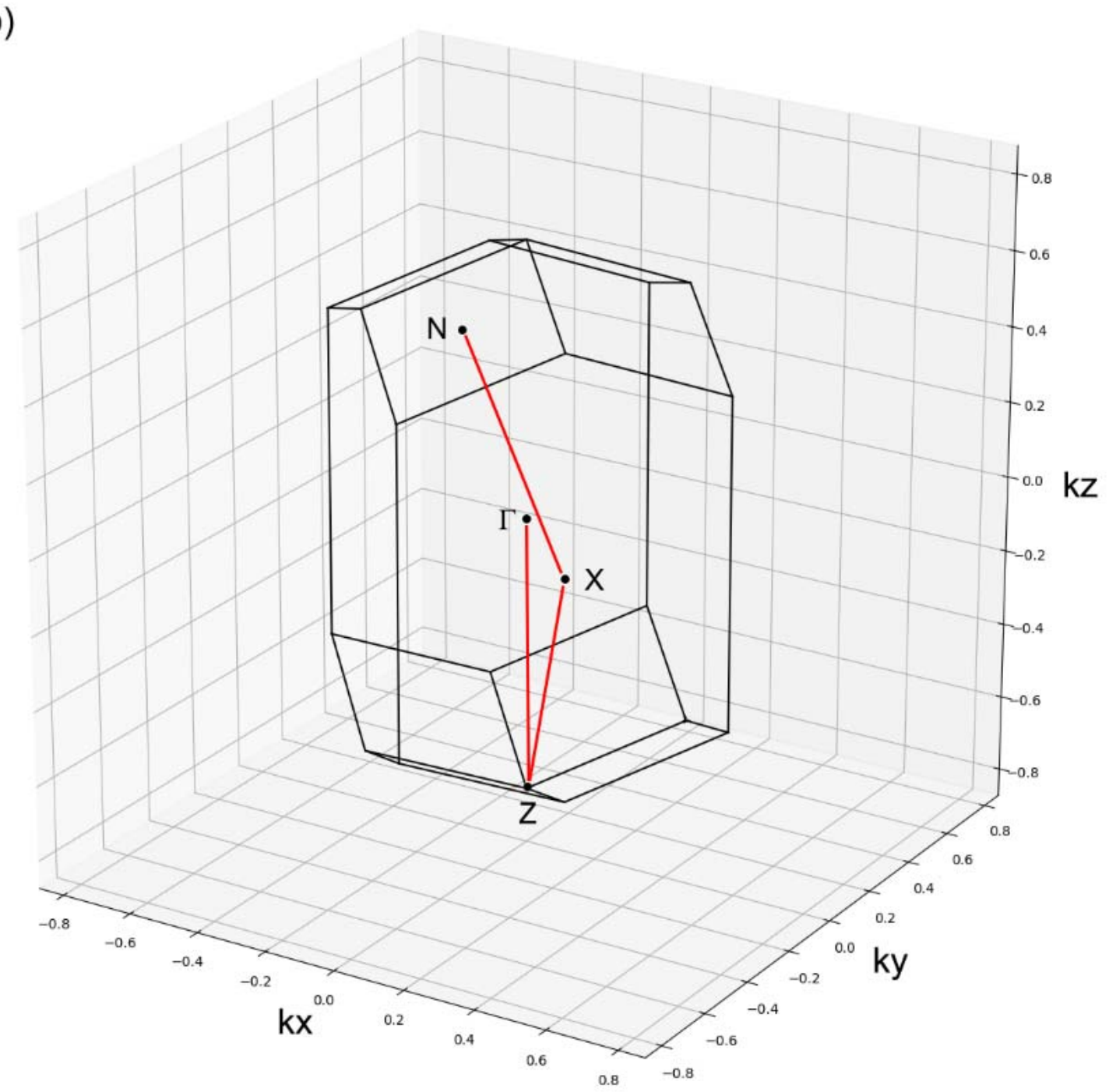

CIF models

Model 1. CASTEP relaxed geometry of the propane-d8 crystal.

```
data_propane
_symmetry_space_group_name_H-M    'P21/N'
_symmetry_Int_Tables_number       14
_symmetry_cell_setting            monoclinic
loop_
_symmetry_equiv_pos_as_xyz
  x,y,z
  -x+1/2,y+1/2,-z+1/2
  -x,-y,-z
  x+1/2,-y+1/2,z+1/2
_cell_length_a                    4.0460
_cell_length_b                    12.2909
_cell_length_c                    6.7877
_cell_angle_alpha                 90.0000
_cell_angle_beta                  90.8972
_cell_angle_gamma                 90.0000
loop_
_atom_site_label
_atom_site_type_symbol
_atom_site_fract_x
_atom_site_fract_y
_atom_site_fract_z
_atom_site_U_iso_or_equiv
_atom_site_adp_type
_atom_site_occupancy
C1    C    0.22988   0.94717   0.22563   0.010  Uiso   1.00
D11   H   -0.03776   0.94680   0.19488   0.025  Uiso   1.00
D12   H    0.35277   0.92171   0.08979   0.025  Uiso   1.00
D13   H    0.30270   1.03164   0.25588   0.025  Uiso   1.00
C2    C    0.32067   0.87189   0.39712   0.010  Uiso   1.00
D21   H    0.58610   0.88012   0.43238   0.025  Uiso   1.00
D22   H    0.19185   0.89844   0.53077   0.025  Uiso   1.00
C3    C    0.24202   0.75254   0.35487   0.010  Uiso   1.00
D31   H   -0.02415   0.73971   0.32867   0.025  Uiso   1.00
D32   H    0.31923   0.69967   0.47760   0.025  Uiso   1.00
D33   H    0.37019   0.72429   0.22264   0.025  Uiso   1.00
```

Model 2. CASTEP relaxed geometry of the propene-d6 crystal.

```
data_propene
_symmetry_space_group_name_H-M    'P212121'
_symmetry_Int_Tables_number       19
_symmetry_cell_setting            orthorhombic
loop_
_symmetry_equiv_pos_as_xyz
  x,y,z
  -x+1/2,-y,z+1/2
  -x,y+1/2,-z+1/2
  x+1/2,-y+1/2,-z
_cell_length_a                    10.4886
_cell_length_b                    10.5613
_cell_length_c                    5.7412
_cell_angle_alpha                 90.0000
_cell_angle_beta                  90.0000
_cell_angle_gamma                 90.0000
loop_
_atom_site_label
_atom_site_type_symbol
_atom_site_fract_x
_atom_site_fract_y
_atom_site_fract_z
_atom_site_U_iso_or_equiv
_atom_site_adp_type
_atom_site_occupancy
C1    C    0.28986   0.12201   0.80282   0.010  Uiso   1.00
D11   H    0.37699   0.12002   0.69960   0.025  Uiso   1.00
D12   H    0.21514   0.05519   0.75352   0.025  Uiso   1.00
C2    C    0.27570   0.20224   0.98107   0.010  Uiso   1.00
D21   H    0.35375   0.26711   1.02373   0.025  Uiso   1.00
C3    C    0.16017   0.21157   1.13200   0.010  Uiso   1.00
D31   H    0.11776   0.30682   1.12135   0.025  Uiso   1.00
D32   H    0.18508   0.19664   1.31640   0.025  Uiso   1.00
D33   H    0.08725   0.14208   1.08232   0.025  Uiso   1.00
C4    C    0.57208  -0.07952   0.02703   0.010  Uiso   1.00
D41   H    0.62426  -0.14852  -0.07737   0.025  Uiso   1.00
D42   H    0.47129  -0.06622  -0.01323   0.025  Uiso   1.00
C5    C    0.62858  -0.01401   0.19745   0.010  Uiso   1.00
D51   H    0.72992  -0.02975   0.23248   0.025  Uiso   1.00
C6    C    0.56419   0.08105   0.35000   0.010  Uiso   1.00
D61   H    0.61271   0.17337   0.34161   0.025  Uiso   1.00
D62   H    0.56805   0.05116   0.53391   0.025  Uiso   1.00
D63   H    0.46403   0.09496   0.30065   0.025  Uiso   1.00
```

Model 3. CASTEP relaxed geometry of the candidate tetragonal propene crystal.

```
data_propene_Ibar4
_symmetry_space_group_name_H-M    'I-4'
_symmetry_Int_Tables_number       82
_symmetry_cell_setting            tetragonal
loop_
_symmetry_equiv_pos_as_xyz
  x,y,z
  -x,-y,z
  y,-x,-z
  -y,x,-z
  x+1/2,y+1/2,z+1/2
  -x+1/2,-y+1/2,z+1/2
  y+1/2,-x+1/2,-z+1/2
  -y+1/2,x+1/2,-z+1/2
_cell_length_a                    10.8145
_cell_length_b                    10.8145
_cell_length_c                    5.3822
_cell_angle_alpha                 90.0000
_cell_angle_beta                  90.0000
_cell_angle_gamma                 90.0000
loop_
_atom_site_label
_atom_site_type_symbol
_atom_site_fract_x
_atom_site_fract_y
_atom_site_fract_z
_atom_site_U_iso_or_equiv
_atom_site_adp_type
_atom_site_occupancy
C1    C    0.12904   0.69600  -0.08570   0.010  Uiso   1.00
H11   H    0.12540   0.61269  -0.19964   0.025  Uiso   1.00
H12   H    0.20477   0.75995  -0.12411   0.025  Uiso   1.00
C2    C    0.04635   0.71780   0.09379   0.010  Uiso   1.00
H21   H   -0.02750   0.65029   0.12604   0.025  Uiso   1.00
C3    C    0.04565   0.82780   0.26142   0.010  Uiso   1.00
H31   H   -0.04191   0.87908   0.24601   0.025  Uiso   1.00
H32   H    0.05275   0.79909   0.45736   0.025  Uiso   1.00
H33   H    0.12203   0.89148   0.22068   0.025  Uiso   1.00
```
